# The nucleocapsid architecture and structural atlas of the prototype baculovirus define the hallmarks of a new viral realm

**DOI:** 10.1126/sciadv.ado2631

**Published:** 2024-12-18

**Authors:** Bronte A. Johnstone, Joshua M. Hardy, Jungmin Ha, Anamarija Butkovic, Paulina Koszalka, Cathy Accurso, Hariprasad Venugopal, Alex de Marco, Mart Krupovic, Fasséli Coulibaly

**Affiliations:** ^1^ Infection Program, Biomedicine Discovery Institute, Monash University, Clayton, Victoria, Australia.; ^2^ Department of Biochemistry and Molecular Biology, Monash University, Clayton, Victoria, Australia.; ^3^ Walter and Eliza Hall Institute of Medical Research, Parkville, Victoria, Australia.; ^4^ Department of Medical Biology, The University of Melbourne, Parkville, Victoria, Australia.; ^5^ Institut Pasteur, Université Paris Cité, CNRS UMR6047, Archaeal Virology Unit, 75015, Paris, France.; ^6^ Ramaciotti Centre for Cryo-Electron Microscopy, Monash University, Clayton, Victoria, Australia.

## Abstract

Baculovirus is the most studied insect virus owing to a broad ecological distribution and ease of engineering for biotechnological applications. However, its structure and evolutionary place in the virosphere remain enigmatic. Using cryo–electron microscopy, we show that the nucleocapsid forms a covalently cross-linked helical tube protecting a highly compacted 134-kilobase pair DNA genome. The ends of the tube are sealed by the base and cap substructures, which share a 126-subunit hub but differ in components that promote actin tail–mediated propulsion and nuclear entry of the nucleocapsid, respectively. Unexpectedly, sensitive searches for hidden evolutionary links show that the morphogenetic machinery and conserved oral infectivity factors originated within the lineage of baculo-like viruses (class *Naldaviricetes*). The unique viral architecture and structural atlas of hallmark proteins firmly place these viruses into a separate new realm, the highest taxonomy rank, and provide a structural framework to expand their use as sustainable bioinsecticides and biomedical tools.

## Teaser

A structural atlas of a beneficial insect virus defines a new virus realm that diversified in the Cambrian.

## INTRODUCTION

Large metagenomic surveys have qualitatively and quantitatively expanded our knowledge of the extraordinary diversity of viruses (*1*, *2*). The prevalence of viruses in all ecosystems means that they largely outnumber cellular organisms including in our own body (*3*, *4*). Besides their ability to cause disease, it has thus become clear that viruses play essential roles in the environment (*5*–*7*) and can have a beneficial impact on human activities (*8*). Baculoviruses are a prime example of the multiple ways in which viruses can be harnessed to contribute to agriculture, biotechnology, and biomedicine (*9*–*11*).

Ever since their discovery, baculoviruses have been studied as bioinsecticides for the sustainable control of invasive insects (*9*, *12*). In turn, these applications have stimulated research on the molecular biology of baculoviruses, enabling a broad range of new biotechnological tools owing to the ease of manipulation of these large DNA viruses. Today, baculoviruses are widely known for their use as vectors for heterologous production of recombinant proteins, underpinning the synergy between scientific research and biotechnological developments. The baculovirus expression system has been used for the development of human vaccines, personalized cancer therapeutics, and the first gene therapy vectors approved for clinical use (*11*).

Despite the extensive exploitation of baculoviruses for various biotechnological applications, the place of baculoviruses and related “baculo-like” viruses of the class *Naldaviricetes* in the virosphere and their provenance remain unresolved (*3*). Baculo-like viruses represent the only group of eukaryotic double-stranded DNA (dsDNA) viruses not assigned to any of the six currently recognized virus realms (*13*), the highest official rank in virus taxonomy (*14*). All other eukaryotic viruses with large dsDNA genomes fall into one of the two realms: *Varidnaviria*, which includes the giant mimivirus, poxviruses, and many more, and *Duplodnaviria*, which comprises tailed bacteriophages and herpesviruses, defined by their distinct morphogenetic modules traceable to prokaryotic virus ancestors (*15*). On the basis of the overlapping gene content, it has been suggested that baculoviruses could be distantly related to varidnaviruses (*16*). However, this connection remains tenuous due to lack of sequence evidence for homology of baculovirus core genes and no structural information on the capsid proteins of baculoviruses.

Baculoviruses differ from other viruses in several respects. Baculoviruses have a large, circular dsDNA genome of 80 to 180 kilo–base pairs replicated in the nucleus of infected cells. They are rare examples of animal DNA viruses with helical nucleocapsids, conferring a large packaging capacity for foreign DNA. Like some other insect viruses, baculoviruses produce two distinct infectious forms. “Classical” secreted particles, called budded virions or BVs, are produced by budding of the nucleocapsid at the plasma membrane (*17*). In addition, occlusion-derived virions or ODVs are produced during late stages of infection, whereby nucleus-retained nucleocapsids are encased with a de novo–assembled lipid envelope and are embedded into micrometer-sized protein crystals called occlusion bodies or polyhedra (*18*, *19*). These ultrastable crystals contain up to several hundreds of virions, allowing long-term persistence of the virus in the environment (*19*).

The nucleocapsid orchestrates key steps in the infection cycle including the packaging of the genome, membrane envelopment, actin tail–driven trafficking within the cells, and breach of the nuclear pore upon genome delivery (*17*, *20*). To understand the provenance and biology of baculo-like viruses, we have determined the structure of the nucleocapsid in the intact BV of the prototypical baculovirus, *Autographa californica* multiple nucleopolyhedrovirus (AcMNPV). We find that the BV nucleocapsid is a cross-linked tube with a unique molecular organization, and discuss key differences with recent structures of baculovirus nucleocapsids (*21*, *22*). In addition, based on a comprehensive structural atlas of the 38 conserved baculovirus proteins, we identify hallmarks in the viral architecture and a set of conserved proteins involved in assembly and virulence that support the independent origin of baculo-like viruses compared to viruses from existing viral realms.

## RESULTS AND DISCUSSION

### The baculovirus nucleocapsid is a helical tube sealed by two multiprotein structures, the viral base and cap

**Fig. 1.**
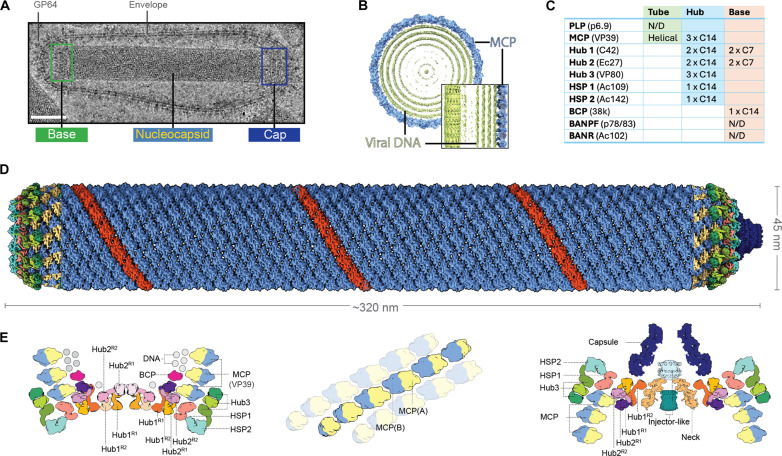
The baculovirus nucleocapsid has a unique helical organization sealed by complex base and cap structures. (**A**) Representative image of a purified AcMNPV BV imaged under cryogenic conditions. Scale bar, 50 nm. (**B**) Clipped, top view of the helical reconstruction of the nucleocapsid with the MCP VP39 represented in blue and the internal DNA in green. Inset: Clipped side view of the nucleocapsid tube. (**C**) Composition of the base and tube including the symmetry and stoichiometry determined by cryo-EM. N/D, not determined. (**D**) Cryo-EM reconstruction of the entire nucleocapsid, assembled from individual reconstructions of the base, tube, and cap. Base (left) and cap (right) colored according to individual subunits. The nucleocapsid tube is colored in blue with an individual strand represented in red. (**E**) Molecular organization of the base (left), tube (middle), and cap (right) as a schematic representation. (Middle) An individual MCP strand of the nucleocapsid helical tube is highlighted with adjacent strands shown in a semitransparent representation.

As expected from a previous work (*23*), the BV particles of AcMNPV imaged by cryo–electron microscopy (cryo-EM) present an outer lipid membrane loosely associated to an inner tubular nucleocapsid that contains the genomic DNA (Fig. 1A). The viral membrane is studded with protein spikes and separated from the nucleocapsid by a tegument layer. Single-particle analysis of these cryo-EM images produced three-dimensional (3D) reconstructions of the helical and base components of sufficient resolution to allow the unambiguous identification of most proteins forming the tube and base of the nucleocapsid (figs. S1 to S4 and tables S1 and S2). Models produced by artificial intelligence–based structure prediction (*24*) were iteratively placed in the electron density map by manual model building and secondary structure matching, and their structures were refined at resolutions of 3.1 and 4.7 Å for the tube and base, respectively (figs. S2 and S3).

The baculovirus nucleocapsid forms a tubular container accommodating the genomic DNA in an internal cavity (Fig. 1B). This architecture is uncommon in virus structures and, for DNA viruses, is only described in archaeal helical viruses (*25*, *26*), the mimivirus genomic fibers (*27*), and white spot syndrome virus, an orphan crustacean-infecting virus distantly related to baculoviruses (*28*, *29*). The two ends of the baculovirus particles, although difficult to distinguish visually from each other in cryo-EM images, were readily classified by image processing (figs. S3 and S4). The base resembles a crown with triangular spikes pointing out of a flat base. Weak electron density is present between the 14 spikes at the center of the base, which suggests the presence of additional protein components heterogeneous in structure or composition (fig. S5). The cap resolved at a resolution of 5.1 Å has a distinct conical shape composed of three components: a distal capsule, an injector-like structure (ILS), and a connecting neck (Fig. 1, A, C, and D).

The main tube of the AcMNPV nucleocapsid is made of 14 helical strands of dimers of the major capsid protein (MCP; also called VP39 or AC89), organized with a C14 cyclic symmetry, as determined by subtomogram averaging and helical reconstruction (figs. S1 and S2 and table S1) and recently reported by others (*21*, *22*). Unexpectedly, the genomic DNA is organized as tightly packed, concentric layers. The DNA strands fill up most of the inner volume of the tube with an interstrand spacing of 27 Å comparable to the 26-Å spacing in the capsids of herpes simplex virus 1 (HSV1), with a similar packing density [0.32 versus 0.36 base pairs (bp)/nm^3^] (Fig. 1B). Thus, compaction of the genome in baculovirus nucleocapsids is similar to the levels observed in highly pressurized capsids of herpesviruses that use packaging motors to achieve pressures up to 18 atm (*30*, *31*). However, no such packaging motor has been identified for baculovirus. It is thus possible that baculovirus achieves the compaction of its genome without active pressurization.

### The main body of the nucleocapsid is fully cross-linked by disulfide bonds

**Fig. 2.**
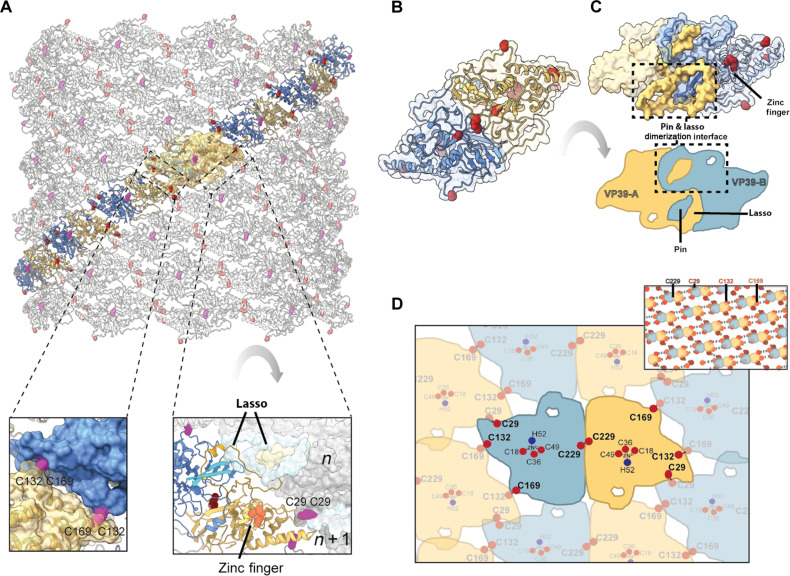
The baculovirus nucleocapsid is a cross-linked helical tube stabilized by intermolecular cross-links and a tight pin/lasso interaction. (**A**) Arrangement of the MCP subunits in the helical tube seen from the outside of the particle. A single, helical strand is highlighted by coloring of the dimer subunits in yellow and blue, with adjacent strands shown in gray. A single asymmetric unit is shown overlaid with the cryo-EM reconstruction as a semitransparent yellow surface. Cysteines potentially involved in intradimer and interdimer disulfide bonds are shown as pink and red spheres, respectively. Bottom left inset: Zoomed-in view of the intrastrand disulfide bond between the adjacent Cys^169^ and Cys^132^ residues. Bottom right inset: Zoomed-in view of the interstrand interactions viewed from the inside of the virion, including the putative Cys^29^-Cys^29^ disulfide bond and associated zinc finger motif, and the β sheet interaction of two adjacent lassos. (**B**) MCP dimer formed by two subunits shown in cartoon and semitransparent surface representations as in (A). (**C**) (Top) MCP dimer, rotated 180° around the *y* axis with respect to (B). The interdimer pin-lasso interaction is shown by darker yellow and blue coloring with respect to the rest of the molecule (black dashed box). (Bottom) Schematic representation of the dimer and pin-lasso interaction. (**D**) Schematic of the disulfide network between adjacent MCP dimers. Top right inset: Extended representation of the disulfide network. The dimer is represented by two circles at the center of mass of each subunit. Disulfide bonds are represented by dashed lines with Cys^29^, Cys^132^, and Cys^169^ shown as maroon, red, and orange spheres, respectively.

The 25-Å–thin wall of the tube presents large gaps in the nucleocapsid surface, which contrast with the tightly sealed surface of most viral capsids (fig. S6A). Thus, baculoviruses face a challenge of packing and stably maintaining a highly compacted genome within a thin shell of loosely associated MCP dimers. Bacteriophages and herpesviruses have evolved two strategies to overcome such a challenge. The most common strategy is capsid stabilization by cementing proteins at weak connecting points between capsomers. Less frequently, the capsids are cross-linked by an intricate chain mail of the MCP, as in the isopeptide bond-stabilized HK97 particles (*32*). The baculovirus adopts a stabilization strategy analogous to the HK97 chain mail, which is based on an extensive network of intermolecular disulfide bonds that covalently cross-link the entire body of the nucleocapsid (Fig. 2A).

The building blocks of the tube are flat, diamond-shaped dimers formed by two triangular MCP subunits (Fig. 2, A to C). The fold of the MCP is novel and has no identifiable homolog in the Protein Data Bank (PDB) and AlphaFold databases of predicted structures (table S3). The N terminus of the MCP contains a CCCH zinc finger motif that does not match known motifs due to an unusual extension in the “knuckle” turn of the zinc finger containing the first two cysteines (Fig. 2A, bottom right inset, and fig. S7A). This extension has a structural role as it projects away from the main body of the MCP to form a potential interdimer disulfide bond between two Cys^29^ residues from neighboring MCP strands in the nucleocapsid (Fig. 2A and figs. S6I and S7A). Zinc fingers are commonly found in nucleic acid–binding proteins but rarely in viral (nucleo)capsid proteins. The only characterized examples are presented by the NC protein of reverse-transcribing viruses (*33*) and sigma 3 of reovirus (*34*). However, the retroviral NC does not assemble into a regular structure (*33*) and sigma 3 interacts with RNA as a dimer in infected cells but not within the capsids. In the baculovirus, the zinc finger motif is oriented toward the lumen of the nucleocapsid tube and contacts inner tubular structures assigned to the viral DNA (Fig. 1A and fig. S7). The zinc finger motif is followed in sequence by an extended β-hairpin (the “pin,” MCP_58-82_), completing the flat face of the MCP that forms the inner surface of the tube (Fig. 2A).

The MCP face exposed to the outside of the tube is composed of a helical domain and a four-stranded β sheet. Decorating this compact fold is a long loop (the “lasso,” MCP_225-296_), which makes up most of the intradimer interface and extends to the inner side of the MCP nucleocapsid (Fig. 2C and fig. S6F). The dimer is further stabilized by two notable features: an intermolecular disulfide bond (Cys^229^-Cys^229^) and a complementary interaction between the pin and the lasso of the two subunits that interlocks the dimer (Fig. 2, C and D, and fig. S6, F and G).

Unexpectedly, each strand is held together through a small interdimer interface burying only 660 Å^2^. However, this interaction forms a highly complementary lock-and-key interaction stabilized by an interdimer disulfide bond between residues Cys^132^ and Cys^169^ (Fig. 2A, left inset, and fig. S6H). Similarly, interfaces between the nucleocapsid strands are relatively small but stabilized by a potential interstrand disulfide bond. The lasso forms an antiparallel β sheet interaction with the lasso from the next strand (Fig. 2A, right inset), whereas the zinc finger extensions from two neighboring subunits project toward another, burying only 483 Å^2^ but potentially locked-in by a disulfide bond between Cys^29^ residues (Fig. 2A, right inset). We experimentally confirmed the presence of disulfide bonds in the AcMNPV capsid (fig. S8). The intrastrand cysteines are conserved across the viral family, whereas the interstrand cysteine pair is specific of alphabaculoviruses. Thus, the entire tube is a covalently interlinked container, which is expected to be extremely stable under oxidizing conditions.

The assembly of the nucleocapsid takes place in the nucleus, which presents a reducing environment in uninfected cells. In baculovirus-infected cells, however, 1 of the 38 core genes conserved in all baculovirus genomes codes for a viral sulfhydryl oxidase (BSOX; known as AC92 or P33) that catalyzes the formation of disulfide bonds in the nucleus of infected cells (*35*). BSOX may work in concert with AC81, a proposed disulfide bond isomerase (*36*). Although BSOX is essential for baculovirus replication, the only BSOX target identified so far is protein PIF-5, which is important for oral infectivity but otherwise dispensable for replication in cell culture (*37*). The key role of inter-MCP disulfide bonds for the nucleocapsid integrity suggests that the MCP is a major target of BSOX. This would explain why BSOX knockout viruses produce aberrant, noninfectious particles, but the molecular mechanism for this BSOX role remains to be elucidated (*35*, *38*).

### MCP quasi-equivalent interactions allow docking of the cap and base at each end of the nucleocapsid

In our cryo-EM reconstructions focused on either end of the nucleocapsid, the last three MCP rings curve inward to accommodate the base and cap components (Fig. 1, C and D, and fig. S9, A and B). The induced curvature in the otherwise straight tube is achieved by local conformational changes particularly in the lasso and the loop containing the interlocking residue Cys^169^ (fig. S9D), affecting the inter- and intrastrand interfaces, respectively. Thus, the MCP dimers form quasi-equivalent contacts in a manner analogous to icosahedral particles that follow the Caspar and Klug principles (*39*, *40*). The thin shell formed by the MCP is reminiscent of the spindle-shaped archaeal viruses, which are the only other known examples of quasi-equivalent helical virions (*25*). This results in three rings of decreasing diameters tapering the nucleocapsid tube to fit the base and cap (fig. S9B).

### A 126-subunit complex forms a hub for additional components in both the base and cap

At each end of the tube, the last two rings of the nucleocapsid tube are encased into a collar formed by the “hub,” a large complex of 126 subunits that functions as a docking platform for other components specific to the base or the cap (Fig. 1C and fig. S9). The hub forms a ring with a 14-fold rotational symmetry (C14) consisting of five proteins that have various names in the literature for historical reasons. Here, we rename them Hub1 (ODV/BV-C42 or AC101), Hub2 (ODV-EC27 or AC144), Hub3 (VP80 or AC104), HSP1 (hub spike protein 1, AC109), and HSP2 (hub spike protein 2, AC142) according to their functions.

As in the ODV nucleocapsid (*22*), the core of the BV hub is a ring of 14 heterotetramers, each composed of two dimers of the Hub1-Hub2 complex, i.e., 14 x [Hub1-Hub2]_2_ (fig. S9, E and F). Hub1 and Hub2 form an intricate complex, with the compact Hub2 at its core and a series of helices in the C terminus of Hub1 (D3_239-350_) wrapping around most of Hub2. In turn, this Hub1-Hub2 complex assembles into a heterotetramer through two separate interfaces. The first interface is formed by homotypic interactions between the Hub1_D2_ domains at the base of the hub. The second one involves the “handle” of one of the Hub2, an extended N-terminal helix, which reaches out to the other Hub1_D3_/Hub2 core. This dimer is highly asymmetrical due to a completely different positioning of the Hub1_D3_/Hub2 core and local differences in the interdomain Hub1 linker (fig. S9F).

The hub ring is built around two main interfaces between the [Hub1-Hub2]_2_ heterotetramers. First, a domain swap of a projecting helix in both Hub2 subunits connects two dimers. Second, Hub1_D2_ domains from two neighboring [Hub1-Hub2]_2_ heterotetramers pack together, positioning their respective cysteine Cys^174^ residues within the disulfide bond distance (fig. S9E). This residue is highly conserved, supporting the biological significance of this interaction. Although these features were not described for the ODV nucleocapsid (*22*), an equivalent electron density is present in the deposited cryo-EM reconstruction (fig. S10, A and B).

Hub3 (VP80) is the only hub component that is not strictly conserved across all baculoviruses (*41*). As in ODVs, Hub3 is located on the outside of both ends of the tube (fig. S9, D and G, and Supplementary Text), which is compatible with a known role in nuclear egress, hijacking actin-myosin complexes (*42*) and a structural role as an attachment point for the “spike” of the hub (described below).

The crown shape of the hub results from the presence of 14 distal spikes attached to the hub (fig. S9H). The spike is a heterodimer of HSP1 and HSP2 (fig. S9I), which may be stabilized by an intermolecular disulfide bond between conserved cysteines Cys^325^ in HSP1 and Cys^174^ in HSP2. This disulfide bond was not reported in the ODV nucleocapsid structure due to a register shift in this region, which may result from modeling errors or biological differences between the BV and ODV infectious forms (fig. S10, C and D). The spikes are not required for the correct assembly of the nucleocapsid (*43*), but mutant viruses without functional HSP1 or HSP2 lack infectivity (*43*–*46*). The location of the spike at the tip of the nucleocapsid proximal to the viral membrane in the virion supports its role in membrane envelopment of the nucleocapsid (*43*). Unenveloped nucleocapsids lack the machinery required for exit from the nucleus to form BVs (*22*), which would explain why cell-to-cell spread is abolished in HSP1 and HSP2 knockouts.

The last lasso of each of the 14 terminal MCPs at both ends of the tube flips out from the pin to provide an anchor for the hub collar (fig. S9, C and D). Thus, the pin is a likely candidate for a switch between the curved organization of MCPs in the hub at each end and the straight MCP tube in the rest of the nucleocapsid. Although it was not modeled in the ODV nucleocapsid, the electron density map is very similar in this area, suggesting that this switch functions in the same way in both infectious forms (fig. S10, E and F).

### The base is decorated by components involved in assembly and trafficking

**Fig. 3.**
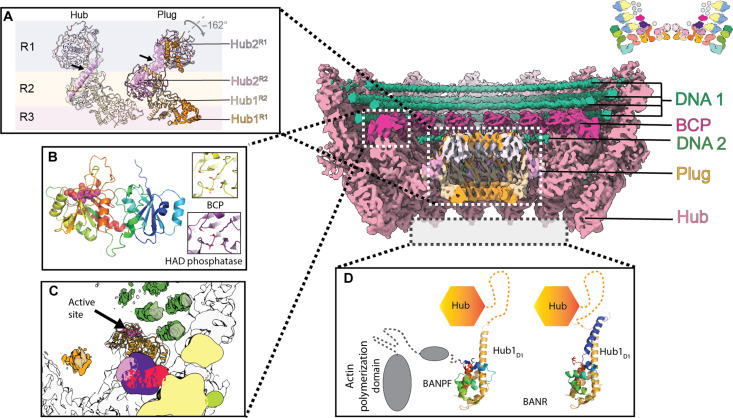
The base comprises components required for genome packaging and actin-tail polymerization. (Main panels) Clipped, side views of the C14 reconstruction of the base colored according to region: hub (pink), DNA (green), baculovirus CTD phosphatase (BCP or 38K; magenta), and plug (orange). (Top right) Schematic of individual subunits that make up the base substructure. (**A**) Cartoon representation of the [Hub1/Hub2]_2_ dimer in the hub (left, transparent ribbon) and the plug (right, opaque ribbon). Hub1 and Hub2 are colored in orange and pink, respectively, with the extended Hub2 handle connecting the R1 and R2 rings highlighted in surface representation to reveal a kinked conformation in the plug for Hub2^R1^ (black arrow). The resulting 162° rotation of the Hub1_D3_/Hub2^R1^ core results in a compact C7 complex plugging the particle base. (**B**) Cartoon representation of BCP highlighting its two-domain organization. There is no homolog of the N-terminal domain, but the CTD is homologous to eukaryotic HAD phosphatases. The putative active site is highly conserved (magenta spheres). A comparison of the active sites of BCP and the Fcp1 phosphatase (PDB ID: 3EF0) is shown in the inset. The six main residues involved in catalysis in HAD phosphatases (Asp^140^-Asp^142^, Ser^177^, Asp^275^-Asp^276^, and Lys^251^) are shown as sticks. They are conserved in sequence and conformation. (**C**) Representation of BCP in the context of the base. The color scheme is the same as the central panel with the BCP active site highlighted in magenta. (**D**) Schematic representation of the hub (orange hexagon) with AlphaFold2 models of the Hub1_D1_ domain, BANPF domain, and BANR protein. Modeling predicts the formation of Hub1_D1_-BANPF and Hub1_D1_-BANR dimers but not a heterotrimer.

In the base, but not the cap, the hub accommodates a large chamber-like structure that forms a “plug” (Fig. 3, bottom middle). The plug protrudes on the inside of the tube, suggesting a possible role in organizing the genomic material. The plug is encircled by a smaller ring assigned to DNA (base DNA or bDNA) that is reminiscent of the “anchor” DNA found around herpesvirus portal complexes (*47*). Like the core of the hub, the plug is composed of Hub1-Hub2 complexes (Fig. 3A). However, here, the complexes assemble as a tighter ring with C7 cyclic symmetry that fits within the C14 hub (fig. S11A). We identified two discrete conformations of the plug, which may be due to the C14/C7 symmetry mismatch (fig. S11; cf. Supplementary Text). Relative to the Hub2 orientation in the hub, the inner Hub2 subunit rotates by 162° toward the central axis of the particle due to a sharp kink in the handle of the other Hub2 subunit. This conformational shift effectively closes the chamber-like structure, sealing the basal end of the nucleocapsid (Fig. 3A). Neighboring components (BCP and bDNA) would clash with the hub-like conformation of the Hub1-Hub2 heterodimer, which may explain why Hub1-Hub2 adopts a closed conformation in the plug.

The inner side of the hub is decorated by 14 copies of the baculovirus C-terminal domain (CTD) phosphatase (BCP; also known as 38K or AC98) protein, which was not known to be a structural component of the nucleocapsid before an independent structure of the ODV (Fig. 3, B and C) (*22*). Sequence and structural analyses identify BCP as a member of the haloacid dehydrogenase (HAD)-like superfamily of phosphatases (table S3) (*48*). The phosphatase domain of BCP is highly conserved, with the key active site residues characteristic of cellular HAD phosphatases being preserved (Fig. 3B). BCP is positioned on the inner side of the base, with a fully accessible active site facing the genomic DNA (Fig. 3C). Phosphatase activity of BCP mediates dephosphorylation of the baculovirus protamine-like protein (PLP; also known as p6.9 or AC100), which is essential for nucleocapsid assembly (*48*). This posttranslational modification restores the polycationic nature of PLP, which can then neutralize the negative charges of the viral DNA backbone. This neutralization allows a high level of compaction as seen in a cellular context for the protamine-mediated condensation of the sperm DNA. Thus, this organization provides an elegant mechanism for the in situ processing of PLP by BCP within assembled particles, presumably during the initial phase of genome packaging.

Two proposed components of the base are missing from our structure: the baculovirus actin nucleation promoting factor (BANPF; previously known as p78/83 or AC9) and the baculovirus actin nucleation regulating protein (BANR; previously known as AC102) (*49*, *50*). Unexpectedly, modeling and a structure-based sequence alignment reveal that BANR and the N terminus of BANPF have homologous folds, suggesting that they likely emerged following a gene duplication event (Fig. 3D). BANPF contains an actin-binding WH2 (WASP-homology 2) domain and is predicted to be homologous to the human neural Wiskott-Aldrich syndrome protein (N-WASP), a regulator of actin polymerization (table S4). BANPF is known to promote the formation of propelling actin comets by stimulation of the Arp2/3 complex (*20*). Consistent with the biochemical data (*49*), our modeling confidently predicts that both BANPF and BANR can form complexes with Hub1_D1_ and appear to be competing for the same binding site in Hub1 (fig. S12). This interaction places these proteins into the diffuse electron density that projects outside of the base (Fig. 3 and fig. S5), which is compatible with their function in actin-tail polymerization to propel the nucleocapsid within the cell during entry and egress. When accessible, Hub1_D1_ acts as a degron, which induces the K48-linked ubiquitination of Hub1 and its degradation, unless it is in complex with BANR (*49*). Thus, BANR allows the stabilization and nuclear import of Hub1. Given the overlapping binding sites of BANPF and BANR on Hub1_D1_, it is possible that BANPF protein displaces BANR in the base upon assembly and/or maturation of the virion, allowing spatiotemporal regulation of the BANPF actin-tail polymerization activity.

### The cap forms an elaborate structure resembling a viral DNA portal

**Fig. 4.**
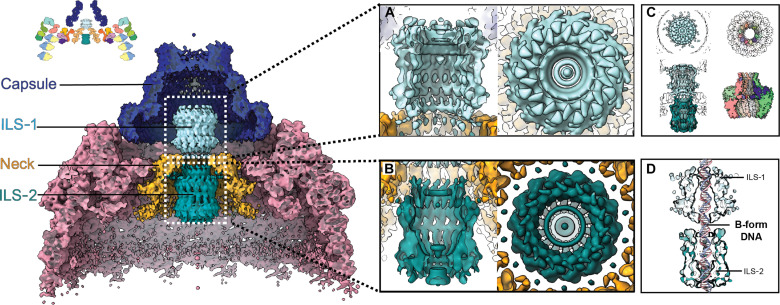
The nucleocapsid cap is composed of an outer capsule and an inner ILS. (Main panels) Clipped, side views of the C14 reconstruction of the cap colored according to region: hub (pink), DNA (green), neck (yellow), injector-like structures ILS-1 and ILS-2 (light blue and teal, respectively), and capsule (dark blue). (Top left) Schematic of individual subunits that make up the base and cap substructures. (**A** and **B**) Zoomed view of the electron density map for ILS-1 and ILS-2, respectively, viewed as in the main panel but clipped (left) and from the top of the particle (right). (**C**) Orthogonal views of the electron density of the AcMNPV ILS (left) and the herpesvirus portal structure in surface representation (PDB ID: 6OD7; right). (**D**) Clipped view of the ILS (surface representation of the single-particle analysis cryo-EM reconstruction) reveals a diameter sufficient to accommodate B-form dsDNA (model).

At the opposite end of the particle, our cryo-EM reconstruction of the cap reveals a three-component module with a distal capsule, an ILS, and an adaptor neck (Fig. 4). At a resolution of 5.1 Å, the localized reconstruction presented a well-defined hub but did not allow model building or identification of the cap-specific components. The capsule that seals the nucleocapsid is a three-tier hollow structure that docks onto the hub spike through HSP2 at the most distal end of the nucleocapsid tube. The capsule is likely to seal the nucleocapsid upon completion of packaging similarly to stopper proteins that plug the portal of tailed bacteriophages, preventing DNA escape from mature viral head particles (*51*).

The capsule is positioned at the tip of the cap, which has been shown to direct the intranuclear envelopment of nucleocapsid during morphogenesis of ODVs and to dock onto the nuclear pore during entry. The top, middle, and bottom tiers have outer diameters of 11, 16, and 23.6 nm, respectively, which are smaller than the dimensions of the inner channel in the nuclear pore complex (64 nm). The maximum diameter of the nucleocapsid is very similar to the width of HIV-1 capsid (54 nm versus ~60 nm, respectively), which can pass through the NPC as intact particles (*52*).

The inner space of the capsule is largely hollow in our reconstruction, except for the ILS at its center. This tubular substructure is split into two 9-nm-long chambers (ILS-1 and ILS-2) connected to each other. The ILS is surrounded by a neck that connects it to the hub through the Hub1_D2_ component of the hub (Fig. 4). The neck of the cap occupies a similar position as the plug in the base. Thus, conceivably, the neck is also composed of a Hub1-Hub2 heterodimer but the electron density map does not allow conclusive identification of this component.

**Fig. 5.**
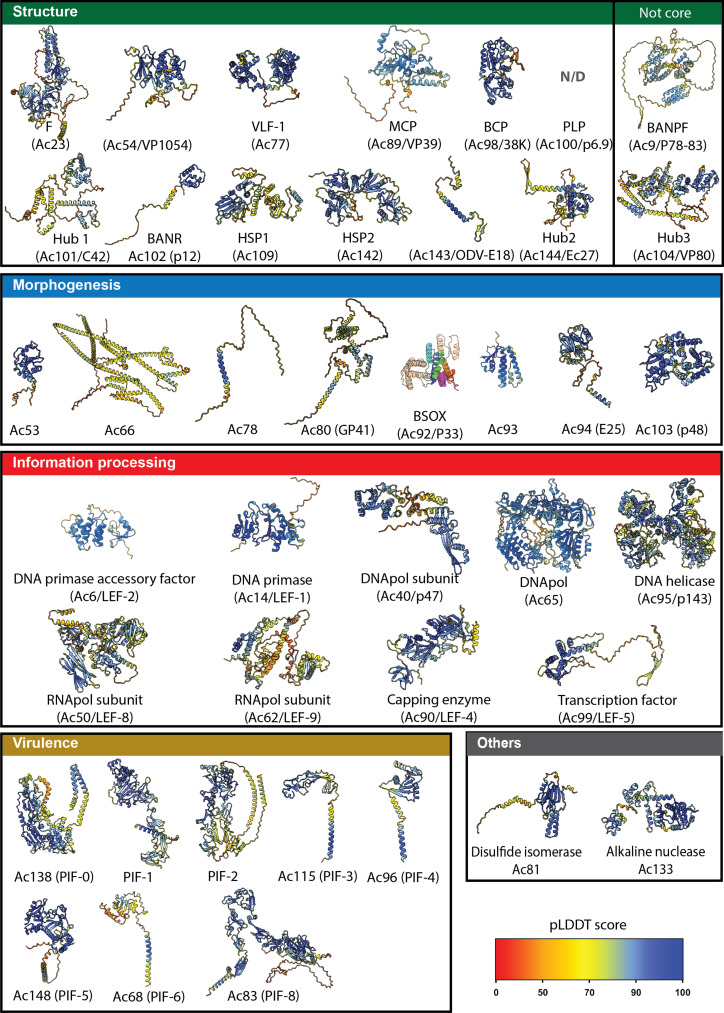
3D atlas of the three core modules of baculoviruses. AlphaFold models generated for the 38 conserved proteins of the core baculovirus genome, shown as cartoon representation and colored according to confidence as estimated by the predicted local distance difference test score (pLDDT). Regions with pLDDT scores of 70, 80, and 90 are considered as acceptable, confident, and highly confident, respectively. Models for Hub3 and BANPF are also included even if these proteins are not conserved across all baculoviruses because they are discussed in the main text. A crystal structure of P33 is included instead of an AlphaFold model because it was available prior to this study (PDB ID: 3P0K; colored as in Fig. 6 and fig. S13). The models are available for download in data S2.

The two ILS chambers are very similar in their ultrastructure but organized in a head-to-head orientation. Both ILS-1 and ILS-2 are composed of an elongated barrel, open where the two chambers come together and closed by a cork-like structure at the other end (Fig. 4, A and B). Given their high similarity, it seems likely that ILS-1 and ILS-2 are composed of the same proteins. Although cross-linking mass spectrometry has not allowed the identification of the components in the cap, plausible candidates include VLF-1, VP1054, and AC66, which are essential for morphogenesis. Only AC66 has a predicted structure compatible with the elongated electron density, but this model is of poor reliability (Fig. 5), and no conclusive fit was obtained.

Although the ILS and herpesvirus portals share similar chamber dimensions and constrictions, we do not infer that they have a common evolutionary origin or function (Fig. 4C). They are wide enough to accommodate dsDNA in its B-form for most part (Fig. 4D), but the passage is closed at the distal ends of each chamber in the mature BV nucleocapsid. In contrast to packaging systems well described in bacteriophages and herpesviruses, no motor protein has been identified in baculoviruses and the mature genome is a circular molecule that is topologically incompatible with the packaging machinery of tailed bacteriophages and herpesviruses.

### Baculovirus is the prototype of a proposed new realm

The place of baculo-like viruses in the global virosphere and their relationship to other DNA viruses have remained elusive for decades. In official taxonomy, baculo-like viruses are classified into the class *Naldaviricetes* (*13*, *53*), which is not assigned to either *Varidnaviria* or *Duplodnaviria* realms of viruses with large dsDNA genomes. Unofficially, however, it has been suggested that baculo-like viruses could have evolved from either of these vast realms. The two realms are defined based on unrelated morphogenetic modules centered around the double jelly roll (DJR)-fold and HK97-fold MCPs, respectively, coupled with distinct genome packaging adenosine triphosphatases (ATPases) (*3*). These viruses adopt a diversity of architectures, but none of them presents a helical nucleocapsid comparable to the one of baculoviruses.

**Fig. 6.**
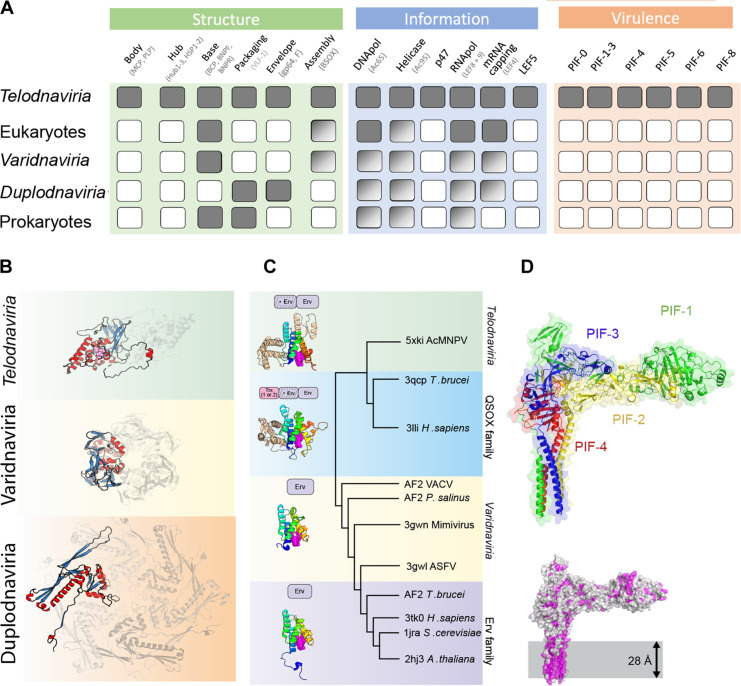
Baculovirus hallmark genes define a proposed new realm. (**A**) List of hallmark gene products in baculovirus mapped to their closest homologs identified by sequence or structural similarity as described in table S4 and data S1. For each functional subcomponent of the conserved core modules, a solid square indicates evidence for homology. Gradient squares indicate similarity without a clear homology relationship. (**B**) The VP39-fold, DJR-fold, and HK97-fold MCPs, the signatures of the *Telodnaviria*, *Varidnaviria* (e.g., giant mimivirus and poxviruses), and *Duplodnaviria* (e.g., tailed bacteriophages and herpesviruses) realms, are depicted on green, yellow, and orange backgrounds, respectively. α-Helices and β-strands are colored in red and blue, respectively. Side chains of the zinc finger are shown as magenta spheres. The MCPs are shown in the context of typical oligomers constituent of the infectious particles (viewed from the inside of the capsid). (**C**) Structure-based relationships between cellular and viral sulfhydryl oxidases represented as a cluster dendrogram constructed based on the pairwise *z*-score comparisons calculated using DALI. (Left) Domain organizations for proteins in the four highlighted groups of sulfhydryl oxidases and representative structures (PDB IDs: 3tk0, 3gwn, 3lli, and 3pk0) and their cartoon representation with the Erv domain colored as a rainbow and unique domains in the baculovirus and QSOX proteins in light brown. The FAD molecule is shown as magenta spheres. (**D**) (Top) Cartoon representations of the AlphaFold model of the putative PIF-1/PIF-2/PIF-3/PIF-4 complex (green, yellow, blue, and red, respectively). (Bottom) Surface representation highlighting hydrophobic regions (magenta). The N-terminal α helices of the four PIFs are likely to be embedded in the viral membrane (gray box).

Here, we show that the baculovirus MCP has a novel fold and intricate molecular organization in the infectious particle. Although Jia *et al.* (*22*) proposed that the AcMNPV MCP is similar to the MCP of herpes-like viruses, we find no support for this conjecture at either the tertiary or quaternary structural level. Notably, the MCPs from the two other realms of viruses with dsDNA genomes lack the signature structural elements of the baculovirus MCP, including the N-terminal zinc finger, cross-linking cysteines, and the lasso/pin components (Supplementary Text and fig. S13). Furthermore, baculo-like viruses do not encode homologs of either genome packaging ATPase (terminase) or portal protein, two quintessential proteins readily identifiable in herpesviruses and HK97-like phages. Similarly, baculoviruses lack the FtsK-HerA superfamily genome packaging ATPase of poxviruses and related giant viruses, and their MCP bears no resemblance to the DJR MCPs (Fig. 6, A and B).

To further assess the relationships between baculoviruses and other known DNA viruses, we expanded the evolutionary analysis and structural modeling to the core baculovirus genome, which includes 38 genes. The corresponding proteins can be divided into three main groups: the morphogenetic, information processing, and virulence modules. We used sensitive hidden Markov model (HMM)–based analysis to search against various profile HMM databases, including a comprehensive database of large and giant virus proteins (*54*). To investigate more distant evolutionary relationships undetectable by sequence analysis only, we also generated a structural atlas of the core proteins (Fig. 5, figs. S14 and S15, and data S2) and used it for structure-based homology searches against the databases of known experimental and predicted structures (Fig. 6A, table S3, and data S1).

The core proteins constituting the morphogenetic module can be further divided into those directly involved in the structure of the nucleocapsid or envelope (14 proteins) and proteins aiding virion assembly (8 proteins). Sequence and structure searches did not identify any viral homologs outside of the *Naldaviricetes* for the structural proteins forming the viral tube (MCP and PLP) and the hub (Fig. 6A and table S4). Notably, three minor nucleocapsid components are homologous to cellular proteins or nonstructural viral proteins and appear to have been co-opted to provide catalytic activities required for the cellular trafficking (BANPF) or assembly (BCP and VLF-1) of the nucleocapsid. In particular, as mentioned above, BANPF contains a domain homologous to the N-WASP actin nucleation promoting factor broadly distributed in eukaryotic organisms, but this domain is uniquely fused to a nucleocapsid-anchoring N-terminal domain homologous to BANR. The CTD of BCP is homologous to cellular HAD phosphatases, whereas VLF-1, involved in baculovirus genome packaging, is a member of the tyrosine recombinase superfamily, both ubiquitous in cellular organisms and viruses (Fig. 4A, table S4, and data S1). Notably, the role of these proteins is unique to baculo-like viruses because neither HAD phosphatases nor tyrosine recombinases have been implicated in genome packaging or virion formation in other viruses.

Among the tegument and envelope components, only two proteins showed relatively close similarity to membrane fusion proteins widespread in eukaryotic enveloped RNA and DNA viruses. In particular, protein F has evolved from class I membrane fusion proteins and, in our searches, was most closely related to a homolog encoded by the human metapneumovirus (*Pneumoviridae*, negative-sense RNA virus). Although protein F mediates entry of BVs for most baculoviruses, in group I baculoviruses (AcMNPV-like viruses), it functions as a pathogenicity factor rather than a fusogen (*55*). In these viruses, the membrane fusion function of BVs is performed by the GP64 protein, which is a homolog of the class III fusogen encoded by Thogoto virus (*Orthomyxoviridae*, negative-sense RNA virus), consistent with previous observations (*56*). These results underscore the frequent horizontal shuffling of the fusion protein genes between evolutionarily unrelated viruses (*57*).

Last, among the virion assembly factors, only the Erv-like flavin adenine dinucleotide (FAD)-linked sulfhydryl oxidase BSOX involved in disulfide bond formation has homologs in other eukaryotic viruses, namely, in large and giant DNA viruses of the phylum *Nucleocytoviricota* (realm *Varidnaviria*). It has been suggested that this shared character signifies a deeper relationship between baculoviruses and varidnaviruses, in particular, poxviruses (*16*, *58*). However, in profile-profile comparisons, the match between the sulfhydryl oxidase of baculoviruses and poxviruses is limited to a 40–amino acid-long region encompassing the active site (data S1). Sulfhydryl oxidases encoded by poxviruses, exemplified by the E10R protein of vaccinia virus (*59*), fold as a single domain closely similar to that of the cellular Erv family enzymes. By contrast, sulfhydryl oxidases encoded by baculoviruses and other baculo-like viruses, such as nudiviruses, contain an inactivated Erv-like domain, ΨErv, in addition to the catalytically active Erv domain (*60*, *61*). This domain organization is reminiscent of the cellular Quiescin family sulfhydryl oxidases (QSOX), which, in addition to the ΨErv and Erv domains, contain one or two N-terminal thioredoxin-like domains (Fig. 6C and fig. S13). All-against-all structural comparison of the enzymes from diverse viruses and cellular organisms showed that sulfhydryl oxidases of baculoviruses and varidnaviruses cluster with the QSOX and Erv family enzymes, respectively, but not with one another (Fig. 6C, fig. S14, and table S3). This analysis strongly suggests that sulfhydryl oxidases in the two groups of viruses were acquired independently.

Thus, none of the components of the baculovirus morphogenetic module have orthologs in other DNA viruses, supporting an independent provenance of baculoviruses. This finding is further supported by a similar analysis of their virulence and information processing modules (Supplementary Text). The virulence module is characterized by a set of per os infectivity factors (PIFs) that are dispensable for the replication of BVs but essential for the oral infectivity of ODVs. PIF-0 to PIF-3 and PIF-5 are conserved in baculoviruses and all other viruses of the diverse *Naldaviricetes* class. Our analysis suggests that three of the nine PIF virulence factors, PIF-1, PIF-2, and PIF-3, are divergent paralogs (fig. S15A), which evolved through gene duplication, a common path for genome expansion among large DNA viruses (*62*, *63*). We found that a complex of these proteins and PIF-4 can be modeled as a compact membrane-anchored spike (Fig. 6D and fig. S15, B and C). In AcMNPV, the full complex is known to comprise all PIF-0 to PIF-9, except for PIF-5 (*64*). Accordingly, modeling supports a conserved interface between PIF-0 and the PIF-2/PIF-3 dimer providing a composite model for the PIF-0 to PIF-4 surface spike (fig. S15D). Although the full structure and stoichiometry of the complex will require experimental determination, the proposed model shows that the core components of the PIF spike are conserved across *Naldaviricetes* (fig. S15, E and F). We did not find significant similarity at the structural fold level between PIF components and proteins encoded by other viruses. Notably, however, the proposed organization of the spike on the membrane of ODVs is reminiscent of attachment and fusion proteins, which is compatible with the role of the PIF complex in invasion of the target host (*64*, *65*).

Whereas the virulence module is a clear hallmark of baculo-like viruses owing to its lack of counterpart in the virosphere, the information module has a distinct eukaryotic flavor, with highly divergent homologs readily identifiable in both eukaryotes and eukaryotic viruses with large DNA genomes. These proteins provide enzymatic functions required for the viral genome replication and efficient transcription in the late phase of the infection cycle. Although the evolutionary origin of the replication and transcription machineries of baculovirus is complex, as detailed in Supplementary Text, these machineries have structurally divergent components (LEF-4, LEF-5, LEF-8, LEF-9, and p47 for transcription; DNApol, helicase p143, LEF-1, and LEF-2 for replication) that clearly set them apart from those of the two other realms of large DNA viruses (Fig. 6A, table S3, and Supplementary Text).

In conclusion, the experimental and predicted structures of baculovirus core proteins define the main hallmarks of a distinct lineage of large rod-shaped or filamentous DNA viruses. These hallmarks do not connect baculovirus-like viruses to known realms within the virosphere as might have been anticipated by the presence of shared non-core genes with poxviruses and other large DNA viruses. Instead, most components are unique in their fold and function. Thus, the baculovirus emerges as a representative of a new realm characterized by an invasion complex of membrane-anchored PIFs, a morphogenetic sulfhydryl oxidase, and a helical nucleocapsid sealed by multiprotein base and cap substructures. Given the elongated morphology of infectious particles for known members of the proposed realm, we put forward the name “*Telodnaviria*,” derived from the Latin word “telum” for spear.

If baculo-like viruses have not evolved from other known groups of viruses, then how and when did they emerge? Given the hallmark status of PIF proteins, an enveloped ODV-like virus is likely to be ancestral. The F and GP64 spike proteins, homologous to class I and III fusion proteins, respectively, would have thus been acquired subsequently by horizontal transfer providing an alternate morphogenetic pathway based on BV-like particles. For the nucleocapsid, given the central role of the zinc finger domain of the MCP, we hypothesize that this unique capsid protein has evolved de novo in the context of baculo-like viruses from a simple DNA-binding zinc finger protein. The initial function of the precursor protein could be genome condensation, akin to the zinc finger nucleocapsid protein of retroviruses. The exaptation of cellular or nonstructural viral proteins for capsid formation appears to be the major route of de novo virogenesis, with multiple independent examples traceable throughout the history of the virosphere (*66*, *67*). Subsequent evolution has likely entailed further complexification of the nucleocapsid through recruitment of additional cellular and viral proteins, in particular, the VLF-1 recombinase, HAD phosphatase, and WASP family protein to enhance the efficiency of viral packaging and trafficking.

**Fig. 7.**
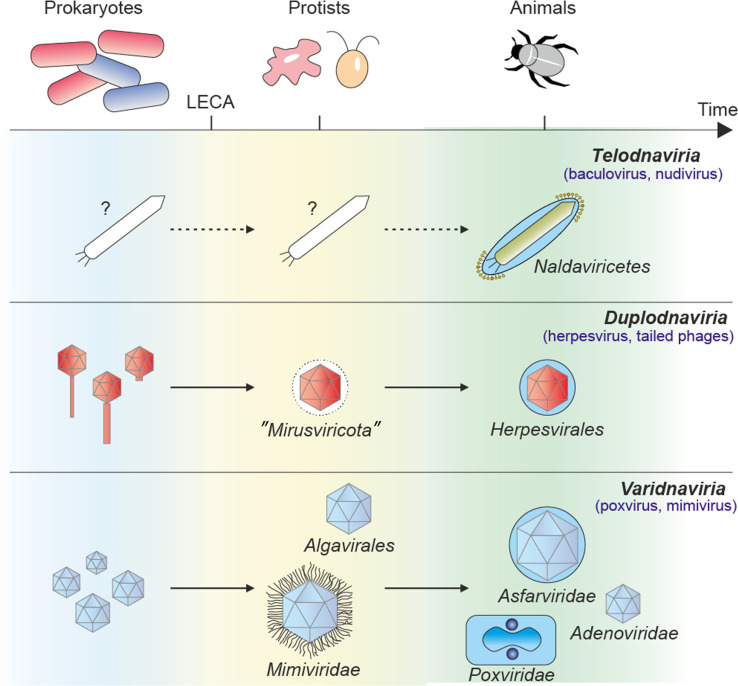
Emergence of viruses from the *Telodnaviria* realm. Schematic representation of the evolutionary timeline of dsDNA viruses approximately aligned with their cellular counterpart. Varidnaviruses and duplodnaviruses infect prokaryotes, protists, and animals. Some members have a lipid membrane schematized by a blue circle in the main diagram. No extant viral relatives of the proposed *Telodnaviria* realm have been described yet in unicellular organisms, indicating a recent radiation in animal hosts. LECA, last eukaryotic cellular ancestor.

The conservation of the PIF complex in viruses with highly diverse invertebrate hosts points to the emergence of baculo-like viruses concomitant with the radiation of arthropods during the Cambrian explosion, ~530 million years ago (Fig. 7). Because baculoviruses largely coevolved with their hosts (*68*), their emergence could predate the divergence of shrimps from insects. This timeline is roughly consistent with the estimated divergence of endogenized bracoviruses from their nudivirus ancestors ~100 million years ago (*69*, *70*).

The apparent absence of baculo-like viruses outside of the arthropod hosts is intriguing, distinguishing them from the other two realms of large DNA viruses that infect both prokaryotes and eukaryotes (Fig. 7). Two features highlight a unique ability of baculoviruses to hijack and remodel the nucleus, which would appear to restrict them to eukaryotic hosts: they promote the formation of intra- and intermolecular disulfide bonds in an environment that is reducing in noninfected cells, and they rely on remodeling of the inner membrane of the nucleus for the envelopment of ODVs. Whether prokaryote-infecting “telodnaviruses” are yet to be discovered or this proposed realm evolved directly by co-optation of cellular components of eukaryotic origin remains to be elucidated through further exploration of virus diversity across biomes.

## MATERIALS AND METHODS

### Amplification and purification of AcMNPV particles

Recombinant AcMNPV (BacToBac, Invitrogen) viruses containing the *Bombyx mori* cypovirus (BmCPV) polyhedrin gene, as previously described (*71*), were amplified in Sf9 cells (Invitrogen) in a suspension culture containing the Insect-XPRESS cell medium (Lonza) with 100 U penicillin/ml, streptomycin (100 μg/ml), and amphotericin (250 μg/ml) (from an antibiotic-antimycotic solution, Merck). The appearance of the BmCPV polyhedrin cytoplasmic crystals coincided with baculovirus amplification and gene expression (*71*), thus was a useful marker for following the infection. A 300-ml culture containing 10^6^ million/ml Sf9 cells was infected with a P4 stock of baculovirus using a 1:250 infection ratio. The cells were incubated until the viability dropped below 80% and most cells contained crystals.

The suspension of infected cells was centrifuged at 1000*g* for 10 min at 4°C to remove the cell debris. The supernatant was further centrifuged at 75,000*g* for 90 min at 4°C using an Optima L-90K Ultracentrifuge (SW41 rotor, Beckman Coulter). The resulting pellet was resuspended in 1 ml of a TE buffer [50 mM Tris and 0.5 mM EDTA (pH 8.7)]. A continuous sucrose gradient (15 to 60%, w/v) was prepared by placing 3 ml of 60% (w/v) sucrose into an ultraclear centrifuge tube (Beckman Coulter), followed by addition of 3 ml each of 45, 30, and 15% (w/v) sucrose into the centrifuge tube taking care not to disrupt the prior layer. The viral solution was loaded onto the sucrose gradient, and the gradient was centrifuged at 77,000*g* for 90 min at 4°C (SW32 rotor, Beckman Coulter). After centrifugation, the virus appears in a visible band at about 2 cm from the bottom of the centrifuge tube. The band was extracted from the gradient and resuspended in 12 ml of the TE buffer to dilute the sucrose. The virus was repelleted at 77,000*g* for 40 min at 4°C. The buffer was removed and the final pellet containing the purified AcMNPV was resuspended in 200 μl of TE. The resuspended viruses were dialyzed in a dialysis device (Tube-O-DIALYZER, Micro, G-Biosciences) against 200 ml of the TE buffer at 4°C overnight to remove any remaining sucrose. The purified virus was stored at 4°C and diluted in the TE buffer as necessary for negative stain and cryo-EM experiments.

### Tomography

A 2.5-μl aliquot of AcMNPV viruses was applied to a glow-discharged R2/2 holey carbon grid (Quantifoil Micro Tools GmbH, Germany) and plunge frozen in liquid ethane using a Vitrobot Mark IV (Thermo Fisher Scientific) with 1-s incubation time, blot time of 2.5 s, blot force of −10, and drain time of 1 s. For fiducial-based alignment, 10-nm colloidal gold was mixed with the sample right before blotting. A total of 37 tilt series were collected on the Titan Krios (×81,000 magnification, 300 keV, with energy filter) from ±60° in 3° increments in a dose-symmetric scheme (*72*), with five frames for each exposure (dose of 2.4 e^−^/Å^2^). Each tilt image contained several frames that were motion corrected and averaged using MotionCor2 (v.1.1.0) (*73*). The averaged images were then aligned in IMOD (v.4.8.56) (*74*) using the gold beads or fiducials as reference points. After alignment, the images were low-pass filtered to increase the signal-to-noise ratio and the tomogram was reconstructed using weighted backprojection (*75*) without contrast transfer function (CTF) correction. To improve contrast, the tomograms were scaled down in Fourier space by a factor of 2. Tomograms without intact viral capsids or with poor alignment were removed, leaving 23 tomograms for subtomogram averaging. A subtomogram with clear helical symmetry and connectivity was used to create a full helical model by copying and fitting the average in real space with adjacent averages in Chimera (v.1.3) (*76*) and then low-pass filtering the map.

### Subtomogram averaging

Coordinates for extraction of subtomograms were set by manually selecting points at either end of each capsid to define the central helical axis using ImageJ (v.1.5) (*77*) and establishing a cylinder of 250-Å radius to approximate the capsid dimensions. Subtomogram averaging was performed using Dynamo (v.1.1.208) (*78*) and Matlab (v.R2015B, The MathWorks Inc.). Boxes of 48 by 48 by 48 pixels were extracted at five-pixel increments along the cylindrical axis, sampling every 10° rotationally. The boxes were translated and rotated into the same orientation relative to the helix and averaged without alignment to generate an initial model for the first iteration. A soft round mask was applied to the reconstructions to minimize Fourier artifacts at the edges of the box. Two rounds of alignment were performed, each round consisting of three iterations using the same parameters (table S4). A total of 94 intact capsids and 7 empty capsids were extracted and averaged (fig. S1).

### Single-particle cryo-EM—Helical nucleocapsid

A 2.5-μl aliquot of AcMNPV viruses was applied to a glow-discharged R2/2 holey carbon grid (Quantifoil Micro Tools GmbH, Germany) and plunge frozen in liquid ethane using a Vitrobot Mark IV (Thermo Fisher Scientific) with zero incubation time, blot time of 2 s, blot force of −10, and drain time of 1 s. Grids were transferred under liquid nitrogen to a Titan Krios transmission electron microscope (FEI/Thermo Fisher Scientific) operated at 300 keV and set for parallel illumination. A total of 1847 movies were recorded using EPU 2 (FEI) on a K2 Summit direct electron detector (Gatan Inc., United States) in super-resolution mode with energy filtering at a calibrated magnification of ×105,000 (table S1).

### Helical symmetry determination

Helical symmetry determination was performed using RELION (v.2.1) (*79*) and SPRING (v.0.84.1470) (*80*). The movies were binned two times by Fourier cropping before motion correction and integrated with MotionCor2 (v.1.1.0) (*73*), giving a final pixel size of 1.34 Å. The CTF parameters of each image were determined using Gctf (v. 1.06) (*81*), and images with notable astigmatism or drift were removed. The remaining micrographs (1794) were used for particle picking and 3D reconstruction. Capsids were boxed manually using the EMAN2 e2helixboxer.py program (v.2.2) (*82*), which were subject to extraction and 2D classification in RELION. Fourier-Bessel indexing of the most popular 2D class in SPRING indicated a rise of 21.1 Å (Bessel order of zero), similar to the 23-Å rise estimated from the subtomogram average, although the cyclic symmetry was ambiguous from both methods, likely C13 or C14. Reconstruction tests in RELION using different cyclic symmetries and helical parameters confirmed a cyclic symmetry of C14 and identified that the rise and twist were double the initial estimate (~43.2 Å and −18.6°), due to the asymmetric unit consisting of a dimer of VP39. This refined map and associated particle coordinates were imported into cryoSPARC (v4.1.2) (*83*) for further processing.

### Helical reconstruction of the capsid

The movies were imported into cryoSPARC and binned two times by Fourier cropping, yielding a final pixel size of 1.34 Å, and subjected to motion correction and CTF estimation using patch motion correction and patch CTF estimation in cryoSPARC (v4.1.2) (fig. S2) (*83*). The refined coordinates from RELION were imported and used to generate a 2D template for automatic filament tracing, resulting in 77,356 particles. Using the 3D reference and helical parameters from RELION, helical refinement was performed in cryoSPARC. During helical refinement, helical parameters were optimized to a rise of 43.22 Å and twist of −18.56°. Refinement of the local defocus values for each particle and estimation of the magnification anisotropy resulted in a nominal resolution of 4.3 Å. Subsequent 3D classification identified one high-resolution class containing most particles (63,595/77,356), and refinement of this particle set resulted in the final resolution of 4.2 Å.

### Focused reconstruction of the capsid

All 77,356 particles from filament tracing were used to generate subparticles for localized reconstruction in cryoSPARC (fig. S2). Using symmetry expansion with a C14 symmetry and helical order of two resulted in 2,165,594 subparticles. To ensure sufficient signal for alignment, a soft mask was generated, which enclosed four neighboring dimers of VP39 in ChimeraX (v.1.6.1) (*84*, *85*). The subparticles were recentered to the mask center, re-extracted with a smaller box size, and locally refined, resulting in a 4.1-Å reconstruction. A 3D classification job with alignments fixed resulted in only two high-resolution classes containing approximately half of the subparticles (1,002,951/2,165,594). Refinement after this classification step resulted in improvement of the resolution to 3.4 Å, and subsequent refinement of per-particle defocus, magnification anisotropy, and beam tilt for each optic group resulted in a final resolution of 3.1 Å. Although C2 symmetry was present due to the VP39 dimerization, application of this symmetry did not improve the resolution of the capsid density and disrupted the density assigned to the genome.

### Single-particle cryo-EM—Base and cap

A 4-μl aliquot of purified AcMNPV viruses, dialyzed into 10 mM Hepes and 0.5 M potassium chloride (pH 7.4), was applied to a glow-discharged R2/2 holey carbon grid (Quantifoil Micro Tools GmbH, Germany) and plunge frozen in liquid ethane using a Vitrobot Mark IV (Thermo Fisher Scientific) with a blot time of 2 s, blot force of −10, and drain time of 1 s. Grids were transferred under liquid nitrogen to a Titan Krios transmission electron microscope (FEI/Thermo Fisher Scientific) operated at 300 keV and set for parallel illumination. A total of 11,439 movies were recorded using EPU 2 (FEI) on a K3 Summit direct electron detector (Gatan Inc., United States) with energy filtering at a calibrated magnification of ×64,000 and a defocus range of 0.8 to 1.4 μm (table S2).

### Reconstruction of the base and cap

The movies were imported into cryoSPARC and subjected to motion correction and CTF estimation using patch motion correction and patch CTF estimation in cryoSPARC (v4.1.2) (*83*). Poor images were removed, yielding 10,628 micrographs for downstream particle picking and 3D reconstruction. Particles were picked and extracted using Topaz (*86*) using a Topaz model trained from manually picked particles from 700 micrographs. Particles were subject to 2D classification and split into base and cap groupings for downstream processing (fig. S3).

For reconstruction of the base, 14,305 particles were used for ab initio modeling and the model refined using homogeneous refinement with C14 symmetry in cryoSPARC. To focus on the base components, particles were recentered and subject to signal subtraction using a mask created using the segmentation tool Segger (*87*) in UCSF Chimera v1.16 (*76*) to remove the signal from additional layers of the capsid body. Subtracted particles were refined using nonuniform refinement in cryoSPARC (*88*) and subject to heterogeneous refinement with two classes. The main class (9379 particles) was subject to CTF refinement before a further nonuniform refinement, followed by 3D classification without alignment to remove the small number of junk particles and remaining particles (9254) used for a final round of nonuniform refinement to yield a reconstruction at 5.0-Å resolution [Fourier Shell Correlaction (FSC) = 0.143].

To improve the resolution of the base, we also performed local refinement on a region containing three asymmetric units. The particle stack was symmetry expanded using C14 symmetry to yield 129,556 particles. Particles were recentered on the trimer and extracted with a smaller box, before density subtraction of the signal outside of the trimer mask and local refinement. Particles were subject to 3D classification with six classes, yielding two major classes (55,047 and 57,203 particles) that differ in the orientation of the plug and were split for further processing. Each class was subject to further focused refinement, CTF refinement, and 3D classification to remove any remaining poor particles, yielding two reconstructions at 4.8- and 4.7-Å resolution (FSC = 0.143).

For reconstruction of the Cap, 11,509 particles were used for ab initio modeling and the model refined using homogeneous refinement with C14 symmetry in cryoSPARC. Particles were subject to signal subtraction as per the base to remove the signal from additional layers of the capsid body. Subtracted particles were refined using nonuniform refinement in cryoSPARC (*88*) and subject to 3D classification without alignment to remove the small number of poor particles and remaining particles (10,905) used for heterogeneous refinement with three classes. Two classes (8557 particles) were kept for further nonuniform refinement with anisotropic magnification refinement to yield a reconstruction at 6.3-Å resolution (FSC = 0.143).

To improve resolution of the cap, we also performed local refinement on a region containing three asymmetric units. The particle stack was symmetry expanded using C14 symmetry to yield 119,798 particles. Particles were recentered on the trimer and extracted with a smaller box, before density subtraction of the signal outside of the trimer mask and local refinement. Particles were subject to 3D classification with six classes, yielding one major classes (53,750 particles) that was subject to further CTF refinement and focused refinement to yield a reconstruction at 5.0-Å resolution (FSC = 0.143).

### DNA packing calculations

To formally compare the packing densities of HSV1 and AcMNPV, we first used the formula π*r*^2^*h*, where *r* and *h* are the inner radius (21.5 nm) and height (311 nm) of the capsid, to calculate the inner volume of the AcMNPV capsid (*V*_capsid_) to be ~451,600 nm^3^. The packing density (ρ_pack_) can then be calculated using a previously derived formula, ρ_pack_ = 0.34 π *N*_bp_/*V*_capsid_ (*89*), where *N*_bp_ denotes the number of base pairs. The calculated ρ_pack_ for AcMNPV was found to be ~0.32 bp/nm^3^, which is comparable to the packing density of 0.36 bp/nm^3^ calculated for HSV1 (*90*).

### Modeling

#### Tube

A model of MCP/VP39, generated using AlphaFold2 (see below), was processed in PHENIX (v.1.20.1) (*91*) and rigid body docked into the map using ChimeraX (v1.5) (*84*, *85*). Modeling was performed in Coot (v.0.9.8.6) (*92*) and refined in real space with the phenix.real_space_refine program (*91*). The model comprises four copies of the VP39 dimer residues Val^15^ to Asn^320^, lacking loop Asp^27^ to Ser^34^, except for subunits where this loop sits in the middle of tetramer assembly and could be fully built (fig. S6). The last 30 residues are glycine/proline-rich and not visible in the reconstruction. This sequence is predicted to form a disordered extension extending outward from the nucleocapsid and is absent in many baculoviruses. The geometry and quality of the models were evaluated using a combination of MolProbity (*93*) and PHENIX.

#### Base

Components present in the base (Hub1/C42, Hub2/EC27, Hub3/VP80, HSP1/Ac109, HSP2/Ac142, and BCP/38K) were identified by manual comparison of the density map and AlphaFold2 models of candidate proteins predicted to be present. Models were rigid body docked in ChimeraX into the focused reconstruction of the base and subject to manual rebuilding in Coot (*92*) and ISOLDE (*94*) before refinement in PHENIX.

### Structure prediction and analysis

A structural atlas of models for all predicted structural proteins and core gene products was generated using the Google ColabFold notebook (*95*) implementation of AlphaFold2 (fig. S12) (*24*). To model the nudivirus MCP, a multiple sequence alignment derived from PSI-BLAST was provided and the baculovirus MCP structure was used as a template.

For model building, secondary structure elements were placed in the cryo-EM reconstructions of the base using Coot (*92*). The most likely components of our structural atlas were iteratively superimposed with these partial models by secondary structure matching, rigid body fitted, and manually rebuilt in the electron density map. All components were placed with unambiguous assignment given the quality of the fit with the experimental electron density map throughout most of the protein chains (fig. S15). The models were visualized with PyMOL and ChimeraX, interfaces analyzed with the PDBePISA server (*96*) and conserved surfaces identified with the Consurf server using default parameters (*97*).

## Supplementary Materials

### The PDF file includes:

Supplementary Text Figs. S1 to S15 Tables S1 to S4 Legends for data S1 and S2 References

## References

[1] M. Shi, X. D. Lin, J. H. Tian, L. J. Chen, X. Chen, C. X. Li, X. C. Qin, J. Li, J. P. Cao, J. S. Eden, J. Buchmann, W. Wang, J. Xu, E. C. Holmes, Y. Z. Zhang, Redefining the invertebrate RNA virosphere. Nature 540, 539–543 (2016). 27880757 10.1038/nature20167

[2] Y. I. Wolf, S. Silas, Y. Wang, S. Wu, M. Bocek, D. Kazlauskas, M. Krupovic, A. Fire, V. V. Dolja, E. V. Koonin, Doubling of the known set of RNA viruses by metagenomic analysis of an aquatic virome. Nat. Microbiol. 5, 1262–1270 (2020). 32690954 10.1038/s41564-020-0755-4 PMC7508674

[3] E. V. Koonin, V. V. Dolja, M. Krupovic, A. Varsani, Y. I. Wolf, N. Yutin, F. M. Zerbini, J. H. Kuhn, Global organization and proposed megataxonomy of the virus world. Microbiol. Mol. Biol. Rev. 84, e00061-19 (2020). 32132243 10.1128/MMBR.00061-19 PMC7062200

[4] L. F. Camarillo-Guerrero, A. Almeida, G. Rangel-Pineros, R. D. Finn, T. D. Lawley, Massive expansion of human gut bacteriophage diversity. Cell 184, 1098–1109.e9 (2021). 33606979 10.1016/j.cell.2021.01.029 PMC7895897

[5] J. R. Brum, J. C. Ignacio-Espinoza, S. Roux, G. Doulcier, S. G. Acinas, A. Alberti, S. Chaffron, C. Cruaud, C. de Vargas, J. M. Gasol, G. Gorsky, A. C. Gregory, L. Guidi, P. Hingamp, D. Iudicone, F. Not, H. Ogata, S. Pesant, B. T. Poulos, S. M. Schwenck, S. Speich, C. Dimier, S. Kandels-Lewis, M. Picheral, S. Searson, P. Bork, C. Bowler, S. Sunagawa, P. Wincker, E. Karsenti, M. B. Sullivan, Ocean plankton. Patterns and ecological drivers of ocean viral communities. Science 348, 1261498 (2015). 25999515 10.1126/science.1261498

[6] M. R. Clokie, A. D. Millard, A. V. Letarov, S. Heaphy, Phages in nature. Bacteriophage 1, 31–45 (2011). 21687533 10.4161/bact.1.1.14942 PMC3109452

[7] M. J. Roossinck, The good viruses: Viral mutualistic symbioses. Nat. Rev. Microbiol. 9, 99–108 (2011). 21200397 10.1038/nrmicro2491

[8] M. Voysey, S. A. C. Clemens, S. A. Madhi, L. Y. Weckx, P. M. Folegatti, P. K. Aley, B. Angus, V. L. Baillie, S. L. Barnabas, Q. E. Bhorat, S. Bibi, C. Briner, P. Cicconi, A. M. Collins, R. Colin-Jones, C. L. Cutland, T. C. Darton, K. Dheda, C. J. A. Duncan, K. R. W. Emary, K. J. Ewer, L. Fairlie, S. N. Faust, S. Feng, D. M. Ferreira, A. Finn, A. L. Goodman, C. M. Green, C. A. Green, P. T. Heath, C. Hill, H. Hill, I. Hirsch, S. H. C. Hodgson, A. Izu, S. Jackson, D. Jenkin, C. C. D. Joe, S. Kerridge, A. Koen, G. Kwatra, R. Lazarus, A. M. Lawrie, A. Lelliott, V. Libri, P. J. Lillie, R. Mallory, A. V. A. Mendes, E. P. Milan, A. M. Minassian, A. M. Gregor, H. Morrison, Y. F. Mujadidi, A. Nana, P. J. O’Reilly, S. D. Padayachee, A. Pittella, E. Plested, K. M. Pollock, M. N. Ramasamy, S. Rhead, A. V. Schwarzbold, N. Singh, A. Smith, R. Song, M. D. Snape, E. Sprinz, R. K. Sutherland, R. Tarrant, E. C. Thomson, M. E. Török, M. Toshner, D. P. J. Turner, J. Vekemans, T. L. Villafana, M. E. E. Watson, C. J. Williams, A. D. Douglas, A. V. S. Hill, T. Lambe, S. C. Gilbert, A. J. Pollard, Oxford COVID Vaccine Trial Group, Safety and efficacy of the ChAdOx1 nCoV-19 vaccine (AZD1222) against SARS-CoV-2: An interim analysis of four randomised controlled trials in Brazil, South Africa, and the UK. Lancet 397, 99–111 (2021). 33306989 10.1016/S0140-6736(20)32661-1 PMC7723445

[9] J. Wagemans, D. Holtappels, E. Vainio, M. Rabiey, C. Marzachi, S. Herrero, M. Ravanbakhsh, C. C. Tebbe, M. Ogliastro, M. A. Ayllon, M. Turina, Going viral: Virus-based biological control agents for plant protection. Annu. Rev. Phytopathol. 60, 21–42 (2022). 35300520 10.1146/annurev-phyto-021621-114208

[10] M. M. van Oers, G. P. Pijlman, J. M. Vlak, Thirty years of baculovirus-insect cell protein expression: From dark horse to mainstream technology. J. Gen. Virol. 96, 6–23 (2015). 25246703 10.1099/vir.0.067108-0

[11] M. L. Pidre, P. N. Arrias, L. C. Amoros Morales, V. Romanowski, The magic staff: A comprehensive overview of baculovirus-based technologies applied to human and animal health. Viruses 15, 80 (2022). 36680120 10.3390/v15010080 PMC9863858

[12] M. D. Summers, “Milestones leading to the genetic engineering of baculoviruses as expression vector systems and viral pesticides” in *Insect Viruses: Biotechnological Applications*, Advances in Virus Research (Elsevier, 2006), pp. 3–73. 10.1016/S0065-3527(06)68001-9 16997008

[13] M. M. van Oers, E. A. Herniou, J. A. Jehle, P. J. Krell, A. M. M. Abd-Alla, B. M. Ribeiro, D. A. Theilmann, Z. Hu, R. L. Harrison, Developments in the classification and nomenclature of arthropod-infecting large DNA viruses that contain pif genes. Arch. Virol. 168, 182 (2023). 37322175 10.1007/s00705-023-05793-8 PMC10271883

[14] International Committee on Taxonomy of Viruses Executive, The new scope of virus taxonomy: Partitioning the virosphere into 15 hierarchical ranks. Nat. Microbiol. 5, 668–674 (2020). 32341570 10.1038/s41564-020-0709-x PMC7186216

[15] M. Krupovic, V. V. Dolja, E. V. Koonin, The virome of the last eukaryotic common ancestor and eukaryogenesis. Nat. Microbiol. 8, 1008–1017 (2023). 37127702 10.1038/s41564-023-01378-y PMC11130978

[16] J. Iranzo, M. Krupovic, E. V. Koonin, The double-stranded DNA virosphere as a modular hierarchical network of gene sharing. mBio 7, e00978-16 (2016). 27486193 10.1128/mBio.00978-16 PMC4981718

[17] G. W. Blissard, D. A. Theilmann, Baculovirus entry and egress from insect cells. Annu. Rev. Virol. 5, 113–139 (2018). 30004832 10.1146/annurev-virology-092917-043356

[18] G. F. Rohrmann, *Baculovirus Molecular Biology* (National Center for Biotechnology Information, ed. 4, 2019). 31294936

[19] F. Coulibaly, E. Chiu, S. Gutmann, C. Rajendran, P. W. Haebel, K. Ikeda, H. Mori, V. K. Ward, C. Schulze-Briese, P. Metcalf, The atomic structure of baculovirus polyhedra reveals the independent emergence of infectious crystals in DNA and RNA viruses. Proc. Natl. Acad. Sci. U.S.A. 106, 22205–22210 (2009). 20007786 10.1073/pnas.0910686106 PMC2799703

[20] E. D. Goley, T. Ohkawa, J. Mancuso, J. B. Woodruff, J. A. D'Alessio, W. Z. Cande, L. E. Volkman, M. D. Welch, Dynamic nuclear actin assembly by Arp2/3 complex and a baculovirus WASP-like protein. Science 314, 464–467 (2006). 17053146 10.1126/science.1133348

[21] F. M. C. Benning, S. Jenni, C. Y. Garcia, T. H. Nguyen, X. Zhang, L. H. Chao, Helical reconstruction of VP39 reveals principles for baculovirus nucleocapsid assembly. Nat. Commun. 15, 250 (2024). 38177118 10.1038/s41467-023-44596-y PMC10767040

[22] X. Jia, Y. Gao, Y. Huang, L. Sun, S. Li, H. Li, X. Zhang, Y. Li, J. He, W. Wu, H. Venkannagari, K. Yang, M. L. Baker, Q. Zhang, Architecture of the baculovirus nucleocapsid revealed by cryo-EM. Nat. Commun. 14, 7481 (2023). 37980340 10.1038/s41467-023-43284-1 PMC10657434

[23] Q. Wang, B. J. Bosch, J. M. Vlak, M. M. van Oers, P. J. Rottier, J. W. M. van Lent, Budded baculovirus particle structure revisited. J. Invertebr. Pathol. 134, 15–22 (2016). 26743500 10.1016/j.jip.2015.12.001 PMC7127228

[24] J. Jumper, R. Evans, A. Pritzel, T. Green, M. Figurnov, O. Ronneberger, K. Tunyasuvunakool, R. Bates, A. Žídek, A. Potapenko, A. Bridgland, C. Meyer, S. A. A. Kohl, A. J. Ballard, A. Cowie, B. Romera-Paredes, S. Nikolov, R. Jain, J. Adler, T. Back, S. Petersen, D. Reiman, E. Clancy, M. Zielinski, M. Steinegger, M. Pacholska, T. Berghammer, S. Bodenstein, D. Silver, O. Vinyals, A. W. Senior, K. Kavukcuoglu, P. Kohli, D. Hassabis, Highly accurate protein structure prediction with AlphaFold. Nature 596, 583–589 (2021). 34265844 10.1038/s41586-021-03819-2 PMC8371605

[25] F. Wang, V. Cvirkaite-Krupovic, M. Vos, L. C. Beltran, M. A. B. Kreutzberger, J. M. Winter, Z. Su, J. Liu, S. Schouten, M. Krupovic, E. H. Egelman, Spindle-shaped archaeal viruses evolved from rod-shaped ancestors to package a larger genome. Cell 185, 1297–1307.e11 (2022). 35325592 10.1016/j.cell.2022.02.019 PMC9018610

[26] D. Ptchelkine, A. Gillum, T. Mochizuki, S. Lucas-Staat, Y. Liu, M. Krupovic, S. E. V. Phillips, D. Prangishvili, J. T. Huiskonen, Unique architecture of thermophilic archaeal virus APBV1 and its genome packaging. Nat. Commun. 8, 1436 (2017). 29127347 10.1038/s41467-017-01668-0 PMC5681674

[27] A. Villalta, A. Schmitt, L. F. Estrozi, E. R. J. Quemin, J. M. Alempic, A. Lartigue, V. Pražák, L. Belmudes, D. Vasishtan, A. M. G. Colmant, F. A. Honoré, Y. Couté, K. Grünewald, C. Abergel, The giant mimivirus 1.2 Mb genome is elegantly organized into a 30-nm diameter helical protein shield. eLife 11, e77607 (2022). 35900198 10.7554/eLife.77607 PMC9512402

[28] H. J. Huang, S. L. Tang, Y. C. Chang, H. C. Wang, T. H. Ng, R. F. Garmann, Y. W. Chen, J. Y. Huang, R. Kumar, S. H. Chang, S. R. Wu, C. Y. Chao, K. Matoba, I. Kenji, W. M. Gelbart, T. P. Ko, H. A. Wang, C. F. Lo, L. L. Chen, H. C. Wang, Multiple nucleocapsid structural forms of shrimp White Spot Syndrome Virus suggests a novel viral morphogenetic pathway. Int. J. Mol. Sci. 24, 7525 (2023). 37108688 10.3390/ijms24087525 PMC10140842

[29] M. Sun, M. Liu, H. Shan, K. Li, P. Wang, H. Guo, Y. Zhao, R. Wang, Y. Tao, L. Yang, Y. Zhang, X. Su, Y. Liu, C. Li, J. Lin, X. L. Chen, Y. Z. Zhang, Q. T. Shen, Ring-stacked capsids of white spot syndrome virus and structural transitions with genome ejection. Sci. Adv. 9, eadd2796 (2023). 36812312 10.1126/sciadv.add2796 PMC9946344

[30] D. Bhella, F. J. Rixon, D. J. Dargan, Cryomicroscopy of human cytomegalovirus virions reveals more densely packed genomic DNA than in herpes simplex virus type 1. J. Mol. Biol. 295, 155–161 (2000). 10623515 10.1006/jmbi.1999.3344

[31] M. Krupovic, E. V. Koonin, Homologous capsid proteins testify to the common ancestry of retroviruses, caulimoviruses, pseudoviruses, and metaviruses. J. Virol. 91, e00210–17 (2017). 28356531 10.1128/JVI.00210-17 PMC5446648

[32] W. R. Wikoff, L. Liljas, R. L. Duda, H. Tsuruta, R. W. Hendrix, J. E. Johnson, Topologically linked protein rings in the bacteriophage HK97 capsid. Science 289, 2129–2133 (2000). 11000116 10.1126/science.289.5487.2129

[33] V. D'Souza, M. F. Summers, Structural basis for packaging the dimeric genome of Moloney murine leukaemia virus. Nature 431, 586–590 (2004). 15457265 10.1038/nature02944

[34] A. M. Olland, J. Jane-Valbuena, L. A. Schiff, M. L. Nibert, S. C. Harrison, Structure of the reovirus outer capsid and dsRNA-binding protein σ3 at 1.8 Å resolution. EMBO J. 20, 979–989 (2001). 11230122 10.1093/emboj/20.5.979 PMC145474

[35] Y. Nie, M. Fang, D. A. Theilmann, Autographa californica multiple nucleopolyhedrovirus core gene ac92 (p33) is required for efficient budded virus production. Virology 409, 38–45 (2011). 20965540 10.1016/j.virol.2010.09.023

[36] H. Zhang, W. Kuang, C. Fu, J. Li, M. Wang, Z. Hu, AC81 is a putative disulfide isomerase involved in baculoviral disulfide bond formation. J. Virol. 96, e0116722 (2022). 36468861 10.1128/jvi.01167-22 PMC9769380

[37] H. Zhang, W. Kuang, C. Chen, Y. Shang, X. Ma, F. Deng, H. Wang, M. Wang, Z. Hu, Per os infectivity factor 5 identified as a substrate of P33 in the baculoviral disulfide bond formation pathway. J. Virol. 94, e00615–20 (2020). 32434885 10.1128/JVI.00615-20 PMC7375385

[38] W. Wu, A. L. Passarelli, Autographa californica multiple nucleopolyhedrovirus Ac92 (ORF92, P33) is required for budded virus production and multiply enveloped occlusion-derived virus formation. J. Virol. 84, 12351–12361 (2010). 20861245 10.1128/JVI.01598-10 PMC2976406

[39] R. Twarock, A. Luque, Structural puzzles in virology solved with an overarching icosahedral design principle. Nat. Commun. 10, 4414 (2019). 31562316 10.1038/s41467-019-12367-3 PMC6765026

[40] D. L. Caspar, A. Klug, Physical principles in the construction of regular viruses. Cold Spring Harb. Symp. Quant. Biol. 27, 1–24 (1962). 14019094 10.1101/sqb.1962.027.001.005

[41] T. Chen, X. Duan, H. Hu, Y. Shang, Y. Hu, F. Deng, H. Wang, M. Wang, Z. Hu, Systematic analysis of 42 Autographa californica multiple nucleopolyhedrovirus genes identifies an additional six genes involved in the production of infectious budded virus. Virol. Sin. 36, 762–773 (2021). 33683665 10.1007/s12250-021-00355-1 PMC8379328

[42] M. Marek, O. W. Merten, F. Francis-Devaraj, M. M. Oers, Essential C-terminal region of the baculovirus minor capsid protein VP80 binds DNA. J. Virol. 86, 1728–1738 (2012). 22090126 10.1128/JVI.05600-11 PMC3264343

[43] C. B. McCarthy, X. Dai, C. Donly, D. A. Theilmann, Autographa californica multiple nucleopolyhedrovirus ac142, a core gene that is essential for BV production and ODV envelopment. Virology 372, 325–339 (2008). 18045640 10.1016/j.virol.2007.10.019

[44] Y. Chen, H. Wu, J. Li, Z. Hu, M. Wang, H. Zhang, Cysteines 128 and 250 are essential for the functions of the baculovirus core gene ac109. Virology 587, 109857 (2023). 37562288 10.1016/j.virol.2023.109857

[45] C. J. Lehiy, W. Wu, M. F. Berretta, A. L. Passarelli, Autographa californica M nucleopolyhedrovirus open reading frame 109 affects infectious budded virus production and nucleocapsid envelopment in the nucleus of cells. Virology 435, 442–452 (2013). 23149091 10.1016/j.virol.2012.10.015

[46] M. Fang, Y. Nie, D. A. Theilmann, Deletion of the AcMNPV core gene ac109 results in budded virions that are non-infectious. Virology 389, 66–74 (2009). 19411088 10.1016/j.virol.2009.04.003

[47] Y. T. Liu, J. Jih, X. Dai, G. Q. Bi, Z. H. Zhou, Cryo-EM structures of herpes simplex virus type 1 portal vertex and packaged genome. Nature 570, 257–261 (2019). 31142842 10.1038/s41586-019-1248-6 PMC6732574

[48] Q. Lai, W. Wu, A. Li, W. Wang, M. Yuan, K. Yang, The 38K-mediated specific dephosphorylation of the viral core protein P6.9 plays an important role in the nucleocapsid assembly of Autographa californica multiple nucleopolyhedrovirus. J. Virol. 92, e01989-17 (2018). 29444944 10.1128/JVI.01989-17 PMC5899202

[49] Y. Zhang, X. Hu, J. Mu, Y. Hu, Y. Zhou, H. Zhao, C. Wu, R. Pei, J. Chen, X. Chen, Y. Wang, Ac102 participates in nuclear actin polymerization by modulating BV/ODV-C42 ubiquitination during Autographa californica multiple nucleopolyhedrovirus infection. J. Virol. 92, e00005–18 (2018). 29618641 10.1128/JVI.00005-18 PMC5974488

[50] S. E. Hepp, G. M. Borgo, S. Ticau, T. Ohkawa, M. D. Welch, Baculovirus AC102 is a nucleocapsid protein that is crucial for nuclear actin polymerization and nucleocapsid morphogenesis. J. Virol. 92, e00111–18 (2018). 29540600 10.1128/JVI.00111-18 PMC5952165

[51] Y. Huang, H. Sun, S. Wei, L. Cai, L. Liu, Y. Jiang, J. Xin, Z. Chen, Y. Que, Z. Kong, T. Li, H. Yu, J. Zhang, Y. Gu, Q. Zheng, S. Li, R. Zhang, N. Xia, Structure and proposed DNA delivery mechanism of a marine roseophage. Nat. Commun. 14, 3609 (2023). 37330604 10.1038/s41467-023-39220-y PMC10276861

[52] V. Zila, E. Margiotta, B. Turoňová, T. G. Müller, C. E. Zimmerli, S. Mattei, M. Allegretti, K. Börner, J. Rada, B. Müller, M. Lusic, H. G. Kräusslich, M. Beck, Cone-shaped HIV-1 capsids are transported through intact nuclear pores. Cell 184, 1032–1046.e18 (2021). 33571428 10.1016/j.cell.2021.01.025 PMC7895898

[53] P. J. Walker, S. G. Siddell, E. J. Lefkowitz, A. R. Mushegian, E. M. Adriaenssens, P. Alfenas-Zerbini, A. J. Davison, D. M. Dempsey, B. E. Dutilh, M. L. García, B. Harrach, R. L. Harrison, R. C. Hendrickson, S. Junglen, N. J. Knowles, M. Krupovic, J. H. Kuhn, A. J. Lambert, M. Łobocka, M. L. Nibert, H. M. Oksanen, R. J. Orton, D. L. Robertson, L. Rubino, S. Sabanadzovic, P. Simmonds, D. B. Smith, N. Suzuki, K. Van Dooerslaer, A. M. Vandamme, A. Varsani, F. M. Zerbini, Changes to virus taxonomy and to the International Code of Virus Classification and Nomenclature ratified by the International Committee on Taxonomy of Viruses (2021). Arch. Virol. 166, 2633–2648 (2021). 34231026 10.1007/s00705-021-05156-1

[54] F. O. Aylward, M. Moniruzzaman, ViralRecall—A flexible command-line tool for the detection of giant virus signatures in 'Omic Data. Viruses 13, 150 (2021). 33498458 10.3390/v13020150 PMC7909515

[55] O. Y. Lung, M. Cruz-Alvarez, G. W. Blissard, Ac23, an envelope fusion protein homolog in the baculovirus Autographa californica multicapsid nucleopolyhedrovirus, is a viral pathogenicity factor. J. Virol. 77, 328–339 (2003). 12477838 10.1128/JVI.77.1.328-339.2003 PMC140606

[56] J. Kadlec, S. Loureiro, N. G. Abrescia, D. I. Stuart, I. M. Jones, The postfusion structure of baculovirus gp64 supports a unified view of viral fusion machines. Nat. Struct. Mol. Biol. 15, 1024–1030 (2008). 18776902 10.1038/nsmb.1484

[57] M. C. Vaney, F. A. Rey, Class II enveloped viruses. Cell. Microbiol. 13, 1451–1459 (2011). 21790946 10.1111/j.1462-5822.2011.01653.x

[58] J. A. Jehle, A. M. Abd-Alla, Y. Wang, Phylogeny and evolution of Hytrosaviridae. J. Invertebr. Pathol. 112, S62–S67 (2013). 22841640 10.1016/j.jip.2012.07.015

[59] T. G. Senkevich, C. L. White, E. V. Koonin, B. Moss, A viral member of the ERV1/ALR protein family participates in a cytoplasmic pathway of disulfide bond formation. Proc. Natl. Acad. Sci. U.S.A. 97, 12068–12073 (2000). 11035794 10.1073/pnas.210397997 PMC17295

[60] Y. Hou, Q. Xia, Y. A. Yuan, Crystal structure of Bombyx mori nucleopolyhedrovirus ORF75 reveals a pseudo-dimer of thiol oxidase domains with a putative substrate-binding pocket. J. Gen. Virol. 93, 2142–2151 (2012). 22764321 10.1099/vir.0.042747-0

[61] M. Hakim, A. Mandelbaum, D. Fass, Structure of a baculovirus sulfhydryl oxidase, a highly divergent member of the Erv flavoenzyme family. J. Virol. 85, 9406–9413 (2011). 21752922 10.1128/JVI.05149-11 PMC3165737

[62] T. B. Machado, A. C. R. Picorelli, B. L. de Azevedo, I. L. M. de Aquino, V. F. Queiroz, R. A. L. Rodrigues, J. P. Araújo Jr., L. S. Ullmann, T. M. D. Santos, R. E. Marques, S. L. Guimarães, A. C. S. P. Andrade, J. S. Gularte, M. Demoliner, M. Filippi, V. M. A. G. Pereira, F. R. Spilki, M. Krupovic, F. O. Aylward, L.-E. Del-Bem, J. S. Abrahão, Gene duplication as a major force driving the genome expansion in some giant viruses. J. Virol. 97, e01309–23 (2023). 38092658 10.1128/jvi.01309-23 PMC10734413

[63] N. C. Elde, S. J. Child, M. T. Eickbush, J. O. Kitzman, K. S. Rogers, J. Shendure, A. P. Geballe, H. S. Malik, Poxviruses deploy genomic accordions to adapt rapidly against host antiviral defenses. Cell 150, 831–841 (2012). 22901812 10.1016/j.cell.2012.05.049 PMC3499626

[64] X. Wang, Y. Shang, C. Chen, S. Liu, M. Chang, N. Zhang, H. Hu, F. Zhang, T. Zhang, Z. Wang, X. Liu, Z. Lin, F. Deng, H. Wang, Z. Zou, J. M. Vlak, M. Wang, Z. Hu, Baculovirus per os infectivity factor complex: Components and assembly. J. Virol. 93, e02053-18 (2019). 30602603 10.1128/JVI.02053-18 PMC6401453

[65] K. Peng, M. M. van Oers, Z. Hu, J. W. van Lent, J. M. Vlak, Baculovirus per os infectivity factors form a complex on the surface of occlusion-derived virus. J. Virol. 84, 9497–9504 (2010). 20610731 10.1128/JVI.00812-10 PMC2937639

[66] M. Krupovic, E. V. Koonin, Multiple origins of viral capsid proteins from cellular ancestors. Proc. Natl. Acad. Sci. U.S.A. 114, E2401–E2410 (2017). 28265094 10.1073/pnas.1621061114 PMC5373398

[67] E. V. Koonin, V. V. Dolja, M. Krupovic, The logic of virus evolution. Cell Host Microbe 30, 917–929 (2022). 35834963 10.1016/j.chom.2022.06.008

[68] J. T. Wennmann, J. Keilwagen, J. A. Jehle, Baculovirus Kimura two-parameter species demarcation criterion is confirmed by the distances of 38 core gene nucleotide sequences. J. Gen. Virol. 99, 1307–1320 (2018). 30045782 10.1099/jgv.0.001100

[69] A. Bézier, M. Annaheim, J. Herbinière, C. Wetterwald, G. Gyapay, S. Bernard-Samain, P. Wincker, I. Roditi, M. Heller, M. Belghazi, R. Pfister-Wilhem, G. Periquet, C. Dupuy, E. Huguet, A. N. Volkoff, B. Lanzrein, J. M. Drezen, Polydnaviruses of braconid wasps derive from an ancestral nudivirus. Science 323, 926–930 (2009). 19213916 10.1126/science.1166788

[70] N. Murphy, J. C. Banks, J. B. Whitfield, A. D. Austin, Phylogeny of the parasitic microgastroid subfamilies (Hymenoptera: Braconidae) based on sequence data from seven genes, with an improved time estimate of the origin of the lineage. Mol. Phylogenet. Evol. 47, 378–395 (2008). 18325792 10.1016/j.ympev.2008.01.022

[71] F. Coulibaly, E. Chiu, K. Ikeda, S. Gutmann, P. W. Haebel, C. Schulze-Briese, H. Mori, P. Metcalf, The molecular organization of cypovirus polyhedra. Nature 446, 97–101 (2007). 17330045 10.1038/nature05628

[72] J. Hutchings, G. Zanetti, Fine details in complex environments: The power of cryo-electron tomography. Biochem. Soc. Trans. 46, 807–816 (2018). 29934301 10.1042/BST20170351 PMC6103461

[73] S. Q. Zheng, E. Palovcak, J. P. Armache, K. A. Verba, Y. Cheng, D. A. Agard, MotionCor2: Anisotropic correction of beam-induced motion for improved cryo-electron microscopy. Nat. Methods 14, 331–332 (2017). 28250466 10.1038/nmeth.4193 PMC5494038

[74] D. N. Mastronarde, S. R. Held, Automated tilt series alignment and tomographic reconstruction in IMOD. J. Struct. Biol. 197, 102–113 (2017). 27444392 10.1016/j.jsb.2016.07.011 PMC5247408

[75] M. Radermacher, T. Wagenknecht, A. Verschoor, J. Frank, A new 3-D reconstruction scheme applied to the 50S ribosomal subunit of E. coli. J. Microsc. 141, RP1–RP2 (1986). 3514918 10.1111/j.1365-2818.1986.tb02693.x

[76] E. F. Pettersen, T. D. Goddard, C. C. Huang, G. S. Couch, D. M. Greenblatt, E. C. Meng, T. E. Ferrin, UCSF Chimera—A visualization system for exploratory research and analysis. J. Comput. Chem. 25, 1605–1612 (2004). 15264254 10.1002/jcc.20084

[77] C. A. Schneider, W. S. Rasband, K. W. Eliceiri, NIH Image to ImageJ: 25 years of image analysis. Nat. Methods 9, 671–675 (2012). 22930834 10.1038/nmeth.2089 PMC5554542

[78] D. Castano-Diez, M. Kudryashev, H. Stahlberg, Dynamo Catalogue: Geometrical tools and data management for particle picking in subtomogram averaging of cryo-electron tomograms. J. Struct. Biol. 197, 135–144 (2017). 27288866 10.1016/j.jsb.2016.06.005

[79] D. Kimanius, B. O. Forsberg, S. H. W. Scheres, E. Lindahl, Accelerated cryo-EM structure determination with parallelisation using GPUs in RELION-2. eLife 5, e18722 (2016). 27845625 10.7554/eLife.18722 PMC5310839

[80] A. Desfosses, R. Ciuffa, I. Gutsche, C. Sachse, SPRING—An image processing package for single-particle based helical reconstruction from electron cryomicrographs. J. Struct. Biol. 185, 15–26 (2014). 24269218 10.1016/j.jsb.2013.11.003

[81] K. Zhang, Gctf: Real-time CTF determination and correction. J. Struct. Biol. 193, 1–12 (2016). 26592709 10.1016/j.jsb.2015.11.003 PMC4711343

[82] G. Tang, L. Peng, P. R. Baldwin, D. S. Mann, W. Jiang, I. Rees, S. J. Ludtke, EMAN2: An extensible image processing suite for electron microscopy. J. Struct. Biol. 157, 38–46 (2007). 16859925 10.1016/j.jsb.2006.05.009

[83] A. Punjani, J. L. Rubinstein, D. J. Fleet, M. A. Brubaker, cryoSPARC: Algorithms for rapid unsupervised cryo-EM structure determination. Nat. Methods 14, 290–296 (2017). 28165473 10.1038/nmeth.4169

[84] T. D. Goddard, C. C. Huang, E. C. Meng, E. F. Pettersen, G. S. Couch, J. H. Morris, T. E. Ferrin, UCSF ChimeraX: Meeting modern challenges in visualization and analysis. Protein Sci. 27, 14–25 (2018). 28710774 10.1002/pro.3235 PMC5734306

[85] E. F. Pettersen, T. D. Goddard, C. C. Huang, E. C. Meng, G. S. Couch, T. I. Croll, J. H. Morris, T. E. Ferrin, UCSF ChimeraX: Structure visualization for researchers, educators, and developers. Protein Sci. 30, 70–82 (2021). 32881101 10.1002/pro.3943 PMC7737788

[86] T. Bepler, A. Morin, M. Rapp, J. Brasch, L. Shapiro, A. J. Noble, B. Berger, Positive-unlabeled convolutional neural networks for particle picking in cryo-electron micrographs. Nat. Methods 16, 1153–1160 (2019). 31591578 10.1038/s41592-019-0575-8 PMC6858545

[87] G. D. Pintilie, J. Zhang, T. D. Goddard, W. Chiu, D. C. Gossard, Quantitative analysis of cryo-EM density map segmentation by watershed and scale-space filtering, and fitting of structures by alignment to regions. J. Struct. Biol. 170, 427–438 (2010). 20338243 10.1016/j.jsb.2010.03.007 PMC2874196

[88] A. Punjani, H. Zhang, D. J. Fleet, Non-uniform refinement: Adaptive regularization improves single-particle cryo-EM reconstruction. Nat. Methods 17, 1214–1221 (2020). 33257830 10.1038/s41592-020-00990-8

[89] P. K. Purohit, M. M. Inamdar, P. D. Grayson, T. M. Squires, J. Kondev, R. Phillips, Forces during bacteriophage DNA packaging and ejection. Biophys. J. 88, 851–866 (2005). 15556983 10.1529/biophysj.104.047134 PMC1305160

[90] W. H. Roos, K. Radtke, E. Kniesmeijer, H. Geertsema, B. Sodeik, G. J. Wuite, Scaffold expulsion and genome packaging trigger stabilization of herpes simplex virus capsids. Proc. Natl. Acad. Sci. U.S.A. 106, 9673–9678 (2009). 19487681 10.1073/pnas.0901514106 PMC2700990

[91] P. D. Adams, P. V. Afonine, G. Bunkóczi, V. B. Chen, I. W. Davis, N. Echols, J. J. Headd, L. W. Hung, G. J. Kapral, R. W. Grosse-Kunstleve, A. J. McCoy, N. W. Moriarty, R. Oeffner, R. J. Read, D. C. Richardson, J. S. Richardson, T. C. Terwilliger, P. H. Zwart, PHENIX: A comprehensive Python-based system for macromolecular structure solution. Acta Crystallogr. D Biol. Crystallogr. 66, 213–221 (2010). 20124702 10.1107/S0907444909052925 PMC2815670

[92] P. Emsley, B. Lohkamp, W. G. Scott, K. Cowtan, Features and development of Coot. Acta Crystallogr. D Biol. Crystallogr. 66, 486–501 (2010). 20383002 10.1107/S0907444910007493 PMC2852313

[93] C. J. Williams, J. J. Headd, N. W. Moriarty, M. G. Prisant, L. L. Videau, L. N. Deis, V. Verma, D. A. Keedy, B. J. Hintze, V. B. Chen, S. Jain, S. M. Lewis, W. B. Arendall III, J. Snoeyink, P. D. Adams, S. C. Lovell, J. S. Richardson, D. C. Richardson, MolProbity: More and better reference data for improved all-atom structure validation. Protein Sci. 27, 293–315 (2018). 29067766 10.1002/pro.3330 PMC5734394

[94] T. I. Croll, ISOLDE: A physically realistic environment for model building into low-resolution electron-density maps. Acta Crystallogr. D Struct. Biol. 74, 519–530 (2018). 29872003 10.1107/S2059798318002425 PMC6096486

[95] M. Mirdita, K. Schütze, Y. Moriwaki, L. Heo, S. Ovchinnikov, M. Steinegger, ColabFold: Making protein folding accessible to all. Nat. Methods 19, 679–682 (2022). 35637307 10.1038/s41592-022-01488-1 PMC9184281

[96] E. Krissinel, K. Henrick, Inference of macromolecular assemblies from crystalline state. J. Mol. Biol. 372, 774–797 (2007). 17681537 10.1016/j.jmb.2007.05.022

[97] H. Ashkenazy, S. Abadi, E. Martz, O. Chay, I. Mayrose, T. Pupko, N. Ben-Tal, ConSurf 2016: An improved methodology to estimate and visualize evolutionary conservation in macromolecules. Nucleic Acids Res. 44, W344–W350 (2016). 27166375 10.1093/nar/gkw408 PMC4987940

[98] M. M. Suhanovsky, C. M. Teschke, Nature's favorite building block: Deciphering folding and capsid assembly of proteins with the HK97-fold. Virology 479-480, 487–497 (2015). 25864106 10.1016/j.virol.2015.02.055 PMC4424165

[99] B. Boogaard, M. M. van Oers, J. W. M. van Lent, An advanced view on baculovirus per os infectivity factors. Insects 9, 84 (2018). 30018247 10.3390/insects9030084 PMC6164829

[100] X. Wang, X. Liu, G. A. Makalliwa, J. Li, H. Wang, Z. Hu, M. Wang, Per os infectivity factors: A complicated and evolutionarily conserved entry machinery of baculovirus. Sci. China Life Sci. 60, 806–815 (2017). 28755302 10.1007/s11427-017-9127-1

[101] D. Kazlauskas, M. Krupovic, J. Guglielmini, P. Forterre, Č. Venclovas, Diversity and evolution of B-family DNA polymerases. Nucleic Acids Res. 48, 10142–10156 (2020). 32976577 10.1093/nar/gkaa760 PMC7544198

[102] U. Rosani, M. Gaia, T. O. Delmont, M. Krupovic, Tracing the invertebrate herpesviruses in the global sequence datasets. Front. Mar. Sci. 10, 1159754 (2023).

[103] S. Shuman, What messenger RNA capping tells us about eukaryotic evolution. Nat. Rev. Mol. Cell Biol. 3, 619–625 (2002). 12154373 10.1038/nrm880

[104] O. J. Kyrieleis, J. Chang, M. de la Peña, S. Shuman, S. Cusack, Crystal structure of vaccinia virus mRNA capping enzyme provides insights into the mechanism and evolution of the capping apparatus. Structure 22, 452–465 (2014). 24607143 10.1016/j.str.2013.12.014 PMC4010090

[105] M. Krupovic, V. V. Dolja, E. V. Koonin, Origin of viruses: Primordial replicators recruiting capsids from hosts. Nat. Rev. Microbiol. 17, 449–458 (2019). 31142823 10.1038/s41579-019-0205-6

[106] L. M. Iyer, E. V. Koonin, D. D. Leipe, L. Aravind, Origin and evolution of the archaeo-eukaryotic primase superfamily and related palm-domain proteins: Structural insights and new members. Nucleic Acids Res. 33, 3875–3896 (2005). 16027112 10.1093/nar/gki702 PMC1176014

[107] J. T. Evans, D. J. Leisy, G. F. Rohrmann, Characterization of the interaction between the baculovirus replication factors LEF-1 and LEF-2. J. Virol. 71, 3114–3119 (1997). 9060674 10.1128/jvi.71.4.3114-3119.1997 PMC191443

