# Supplementary material for: The nucleocapsid architecture and structural atlas of the prototype baculovirus define the hallmarks of a new viral realm
Source: Sci Adv. 2024 Dec 18;10(51):eado2631. doi: 10.1126/sciadv.ado2631 (PMC11654684; doi:10.1126/sciadv.ado2631)
Supplement: Supplementary file 1 — Supplementary Text Figs. S1 to S15 Tables S1 to S4 Legends for data S1 and S2 References [file sciadv.ado2631_sm.pdf]

Science Advances
AAAS

Supplementary Materials for

The nucleocapsid architecture and structural atlas of the prototype baculovirus define the hallmarks of a new viral realm

Bronte A. Johnstone *et al.*

Corresponding author: Fasséli Coulibaly, fasseli.coulibaly@monash.edu

Sci. Adv. **10**, eado2631 (2024)
DOI: 10.1126/sciadv.ado2631

The PDF file includes:

Supplementary Text
Figs. S1 to S15
Tables S1 to S4
Legends for data S1 and S2
References

Other Supplementary Material for this manuscript includes the following:

Data S1 and S2

Supplementary text:

The baculovirus of MCP is not a structural homologue of the HK97-fold MCP

Sequence and structural similarity searches did not identify significant homologues of the baculovirus MCP as described in the main text (Data S1). However, Jia et al (*22*) propose that HK97-fold MCP is similar to the baculovirus MCP prompting for a detailed comparison of the two folds.

On one hand, the HK97-fold is characterized by four structural elements: (1) an extended N-arm with alpha-helical content; (2) an E-loop consisting of a two-stranded beta-sheet; (3) a P-domain characterized by a long beta-sheet in continuation of the E-loop and a parallel spine helix; (4) an A-domain with a beta-hinge domain decorated by short helices (*98*).

On the other hand, the baculovirus MCP fold is characterized by an N-terminal zinc finger motif, interlocking pin/lasso structural elements and crosslinking cysteines. These features are predicted to be conserved across all baculovirus MCP (27), and AlphaFold2 modelling suggests their presence in the MCP of nudivirus (*Nudiviridae* family within the same *Naldaviricetes* class as baculovirus) (Fig. S7).

The pin in the baculovirus MCP is a two-stranded beta-sheet that was compared to the E-loop of HK97-like MCPs by Jia et al (*22*). We find that the beta-sheet has a flipped orientation compared to the HK97 E-loop, with beta-1 instead of beta-2 exposed on the protein surface. In addition, this beta-sheet is not followed by a long beta-strand parallel to the spine helix, which is a signature of the HK97 fold. Instead, it is followed by a helical domain that does contain a 5-turn helix but in a different context than the HK97 spine, notably with the absence of an adjacent long beta-sheet. The extended N-arm and wedge-shaped A-domain of the HK97 fold are not present in the baculovirus MCP. Instead, the N-terminus of MCP contains the conserved zinc-finger motif and the remaining of the protein forms a compact core that differs in topology from the HK97 A-domain.

Importantly, no similarity exists in the functional roles of the different signature elements in assembled particles. HK97-like icosahedral capsids are composed of MCP pentons and hexons. The A-domain forms the core of these capsomers, which are further stabilized by inter-molecular interaction between the E-loop and the extended N-terminal arm. By contrast, the baculovirus nucleocapsid is composed of MCP dimers. The pin/lasso form the core of these capsomers, while the N-terminus interacts with the genomic DNA on the inner face of the particle rather than mediating inter-molecular contacts (Fig. S12). Given its unique fold and assembly mode, we conclude that the baculovirus MCP has no detectable homologue in structurally-characterized viruses.

Core genome-wide analysis supports the independent emergence of baculo-like viruses

Baculoviruses belong to the *Naldaviricetes* class, which comprises four viral families: the *Baculoviridae*, the *Nudiviridae*, the *Hytrosaviridae* and the *Nimaviridae*. We extended the evolutionary analysis to the core *Naldaviricetes* proteins involved in virulence and information processing (Fig. 4a and Table S3). The Virulence module includes nine *per os* infectivity factors (PIF), PIF-0 to PIF-8 conserved in baculoviruses, and an auxiliary PIF (PIF-9 or AC108)(*64*). This module is unique to *Naldaviricetes* (Table S3) and PIF proteins have no identifiable homologues using sequence- and structure-based searches. Interestingly, profile-

profile comparisons revealed that PIF-1, PIF-2 and PIF-3 are homologues of each other (Fig. S14a), suggesting that two of the three genes emerged through duplication. A individual PIF-5 subunit and a multi-protein complex of PIF-0 to -9 (excluding PIF-5) are both required for the infectivity of ODV, but not BV. Indeed, PIF-0 to -5 are conserved across the four families of *Naldaviricetes* except for PIF-4, which is missing in *Hytrosaviridae (13)*. We modelled PIF-0 to -6 and PIF-8 individually (Fig. S9), and various combination of complexes between AcMNPV PIF-0 to -4 (Fig. S14b). We find that PIF-1 to -4 forms a compact core complex (Fig. S14c) as shown previously by biochemical studies (*65, 99, 100*). The topology of these components proposed to project out from the viral membrane is analogous to viral complexes involved in viral attachment and entry and compatible with their role in entry (*99, 100*). Adding further components was not possible with available computational resources and an experimental structure will be required to determine the complete structure and stoichiometry of the spike. However, a convincing interface is predicted between the ectodomains of PIF-0 and the PIF-2/PIF-3 complex (Fig. S14b). This sub-complex allowed us to build a proposed composite model of the PIF-0 to -4 spike (Fig. S14d). Interestingly, the PIF-1 to -3 complex and PIF-0 are predicted to adopt a similar molecular organization in the highly divergent nudiviruses showing conservation within the *Naldaviricetes* class (Fig. S14e,f). The orientation of the ectodomain with respect to the membrane is not predicted with high confidence, so it is unclear if the spike could lay flat on the viral surface as in flaviviruses or project out as in coronaviruses for example. No homodimeric interface was predicted with confidence for PIF-0 to -4, but it is possible that further copies of these PIFs are incorporated in the native complex as well as PIF-6 to PIF-8, as suggested from biochemical data (*64*).

Sequence and structure searches did not identify similar proteins (Data S1, Table S3). Since the PIFs are specific of ODVs, we reviewed other viral (sub)families producing occluded virions, the *Entomopoxvirinae* (enveloped dsDNA viruses) and *Reoviridae* (cypovirus genus, non-enveloped dsRNA viruses). Cypoviruses are non-enveloped and only have 10-12 proteins. Like baculoviruses, entomopoxviruses are large dsDNA viruses (*Varidnaviria*) but they replicate and become occluded in the cytoplasm. No homologues were detected in these viruses by sequence searches or in proteins of known structure supporting the proposal that the PIF complex is a hallmark of the proposed *Telodnaviria* realm (Table S3, Data S1 and Fig. S14).

Unlike in the case of the morphogenetic and virulence modules, the information processing module has clear, but highly divergent, homologues in both eukaryotes and eukaryotic viruses with large DNA genomes. The module provides functions responsible for viral DNA replication, transcription, mRNA capping, etc. The presence of cellular and viral homologues is not surprising given that most eukaryotic large DNA viruses encode replication and transcription machineries which are largely homologous to those of eukaryotes, even if they can be highly divergent. Previous detailed phylogenetic analysis of family B DNA polymerases revealed a complex evolutionary history of these enzymes within *Naldaviricetes*, *Nucleocytoviricota* and *Herpesvirales*. DNA polymerases encoded by viruses within each of the three assemblages are not monophyletic but rather display phylogenetic affinities to different groups of eukaryotic DNA polymerases, either Delta or Epsilon, signifying horizontal transfer and replacement events in the history of each virus group (*101*). DNA polymerases of baculoviruses clustered with Epsilon-like polymerases and formed a distinct monophyletic group, which was only distantly related to the homologues from poxviruses,

malacoherpesviruses and alloherpesviruses (*102*). Consistently, in structure-based comparisons, Epsilon DNA polymerases were identified as the closest structural homologues of the baculovirus protein. Similarly complex evolutionary history is observed with the largest RNA polymerase subunits encoded by baculovirus genes LEF8 and LEF9. At the sequence level, the two baculovirus proteins are not recognizably similar to any viral (outside of the *Naldaviricetes*) or cellular protein. However, in structure-based searches, cellular RNA polymerases were identified as the closest homologues (Table S3, Data S1). Regardless, in the absence of proper phylogenetic analysis (precluded by the extreme sequence divergence of baculovirus LEF8 and LEF9 sequences), the provenance of baculovirus RNA polymerases remains uncertain beyond their closer similarity to homologues from eukaryotes and their viruses. Another component of the RNA polymerase with both eukaryotic and viral homologues is LEF4, an enzyme participating in the synthesis of the mRNA cap structure. It has been previously demonstrated that LEF4 represents a fusion of the N-terminal metal-dependent 5'-triphosphatase domain and the C-terminal guanylyltransferase domain (*103*). Unlike certain *Nucleocytoviricota* viruses, LEF4 does not contain the C-terminal cap methyltransferase domain and in its domain organization is most similar to the capping enzyme of *Entamoeba histolytica (103)*. In our structure-based searches, the triphosphatase domain was most similar to the Cet1 from *Trypanosoma cruzi* (PDB ID: 6l7v), whereas the guanylyltransferase domain was more similar to the archaeal DNA ligase than to structurally related mRNA guanylyltransferases of eukaryotes or viruses. Notably, the structures of capping enzymes from *Nucleocytoviricota* viruses, such as vaccinia virus, phycodnavirus PBCV-1 and mimivirus (*104*), were available in the databases, indicating genuine distinctiveness of the baculovirus proteins. The informational module of baculoviruses also includes helicase Ac95, which is sometimes considered to be related to the D5 primase-helicase of vaccinia virus, a core gene in *Nucleocytoviricota*. However, unlike D5-like proteins, the N-terminal region of Ac95 is unrelated to primases and structural models of this domain do not yield meaningful hits in either DALI or FoldSeek searches. The similarity to D5 proteins is limited to the C-terminal superfamily 3 helicase domain, one of the hallmark virus proteins widespread across viruses with RNA and DNA genomes from all three domains of life (*105*). Indeed, in profile-profile searches the helicase domain of Ac95 is only marginally more similar to the D5 homologues from *Nucleocytoviricota* than to homologues from bacteriophages, with sequence identity between the helicase domains in both cases being ~10% (Table S3, Data S1). Thus, the baculovirus helicase appears to be homologous rather than orthologous to the D5-like primases-helicases of *Nucleocytoviricota* and hence does not provide support for the ancestral relationship between the two virus groups. Instead, baculoviruses encode a genuine eukaryotic-like heterodimeric primase. LEF-1 corresponds to the catalytic DNA primase subunit showing structural similarity to cellular primases of eukaryotes and archaea rather than viral homologues, as noted previously (*106*). Our structure-based searches showed that LEF-2, previously shown to be a cofactor of the LEF-1 primase (*107*), is homologous to the non-catalytic large subunit of archaeo-eukaryotic primases (Table S3). Overall, the information module of baculo-like viruses has unique structural features absent from their closest homologues in eukaryotic organisms. Together with the morphogenetic and virulence modules, it sets the proposed *Telodnaviria* realm apart from other viral realms.

Detailed description of Hub3

Hub3 is the only hub component that is not strictly conserved across all baculoviruses (*41*). Dimers of the Hub3 C-terminal domain sit in a groove between the last two MCP rings of the nucleocapsid (Fig. S8D,G). Their location on the outside of both ends of the tube is compatible with the known role of Hub3 in nuclear egress hijacking actin-myosin complexes (*42*). In addition, a single Hub3 subunit decorates the outerward facing side of the last MCP ring. This distal Hub3 subunit has a structural role as an attachment point with the "spike" of the hub through an alpha-helix that flips out compared to the Hub3 dimer. The N-terminal domains of Hub3 beyond this anchoring alpha-helix have a dispensable role in morphogenesis (*42*) and are not visible in the C14 or localized reconstructions.

The plug displays two conformations

We identified two discrete conformations of the plug, which were not observed in the ODV structure (Fig. S9). One of these conformations closely resembles the plug structure in ODV virions, requiring a rotation of only 2.5° between the plug and the hub (*22*). However, in the second conformation, the plug is rotated halfway between two equivalent plug positions. These two populations of particles indicate that the hub has a stable conformation of almost perfect C14 symmetry (all-atom RMSD of 2.16 Å for non-equivalent $[Hub1/Hub2]_2$ dimers) accommodating the two possible conformations equally well.

Supplementary Figures:

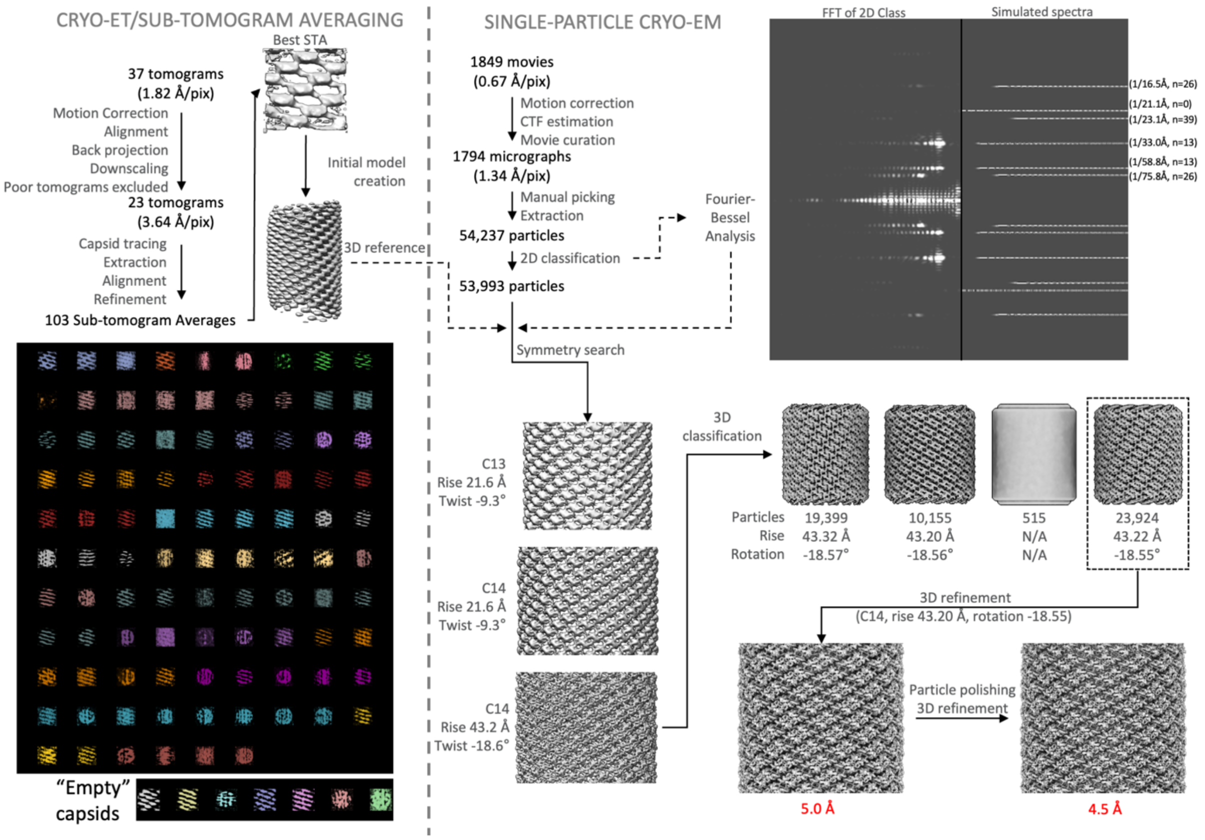

Fig. S1: Cryo-ET and SPA data processing to determine the C14 AcMNPV helical nucleocapsid body.

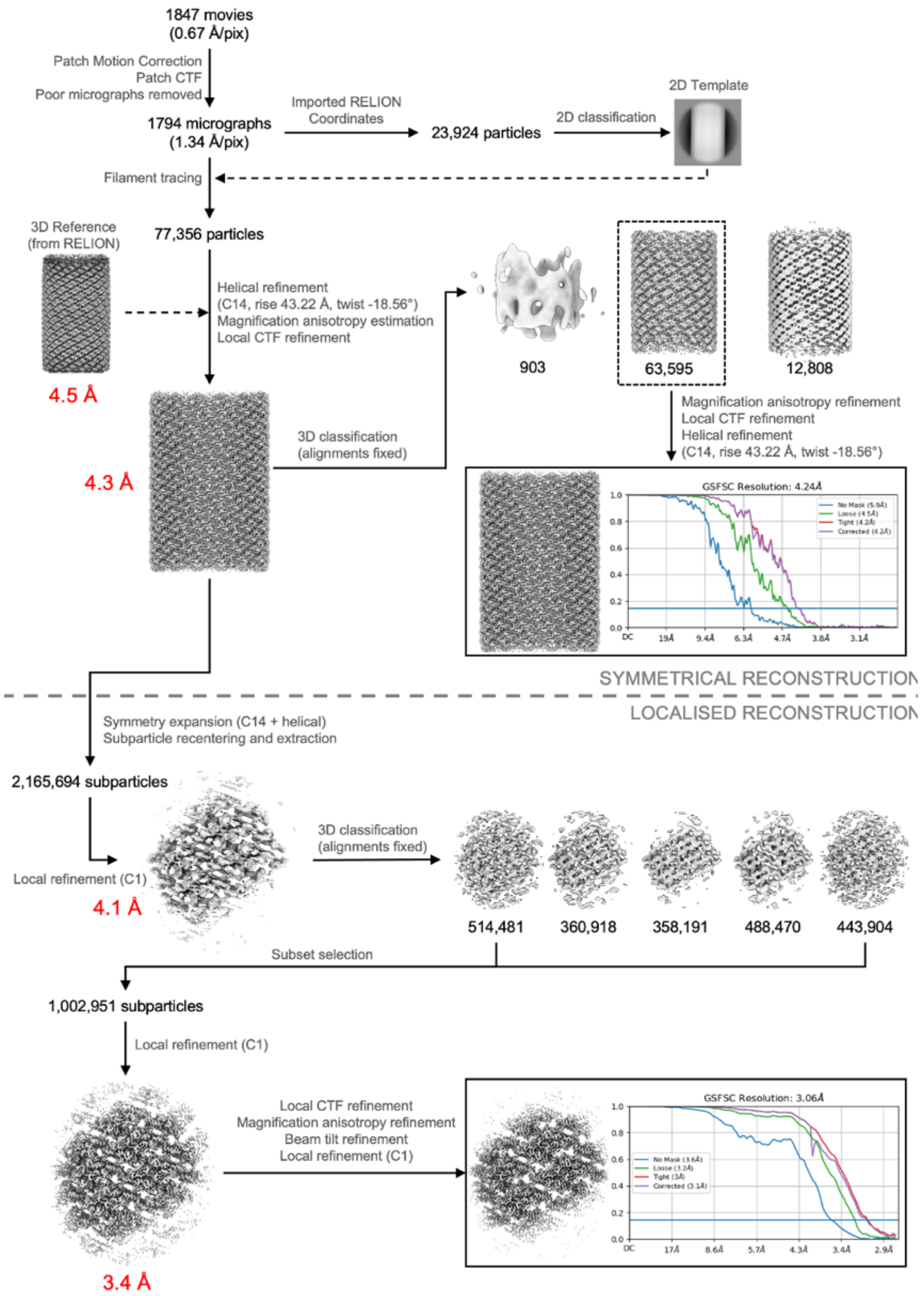

Fig. S2: Cryo-EM data processing workflow to determine the C14 AcMNPV helical nucleocapsid body. The flow chart for the cryo-EM processing of the AcMNPV nucleocapsid body, generating a consensus C14 helical reconstruction (4.24 Å resolution) and focused refinement reconstruction (3.06 Å resolution). FSC curves for each of the final reconstructions are shown and represent gold standard FSC curves (GSFSC) where the 0.143 GSFSC cutoff if indicated by the blue horizontal line.

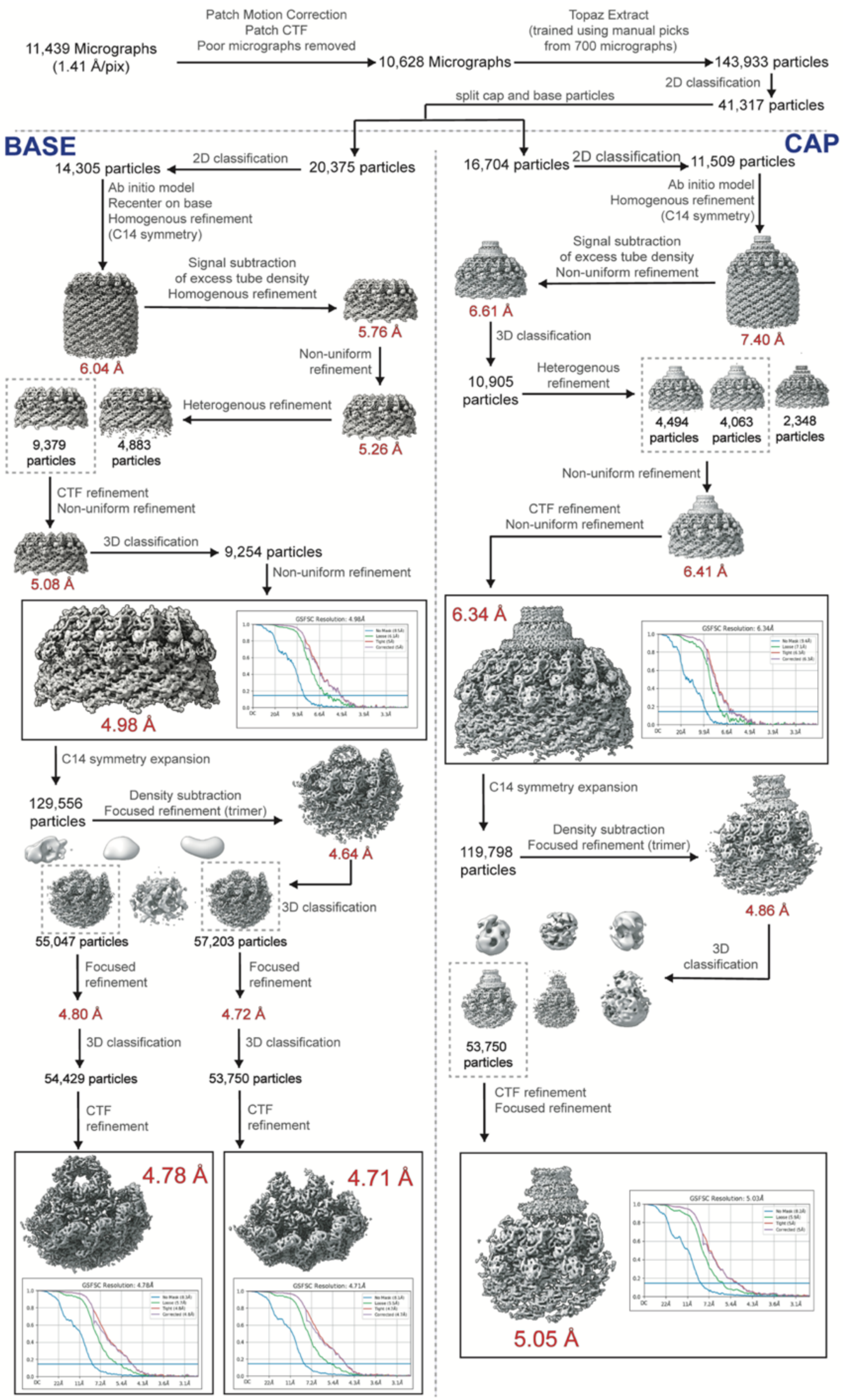

11,439 Micrographs
(1.41 Å/pix)
Patch Motion Correction
Patch CTF
Poor micrographs removed
10,628 Micrographs
Topaz Extract
(trained using manual picks
from 700 micrographs)
143,933 particles
2D classification
41,317 particles
split cap and base particles
BASE
20,375 particles
2D classification
14,305 particles
Ab initio model
Recenter on base
Homogenous refinement
(C14 symmetry)
Signal subtraction
of excess tube density
Homogenous refinement
6.04 Å
5.76 Å
Non-uniform
refinement
5.26 Å
Heterogenous refinement
9,379
particles
4,883
particles
CTF refinement
Non-uniform refinement
5.08 Å
3D classification
9,254 particles
Non-uniform refinement
4.98 Å
GSFSC Resolution: 4.98Å
C14 symmetry expansion
129,556
particles
Density subtraction
Focused refinement (trimer)
4.64 Å
3D classification
55,047 particles
57,203 particles
Focused
refinement
4.80 Å
4.72 Å
3D classification
54,429 particles
53,750 particles
CTF
refinement
4.78 Å
GSFSC Resolution: 4.78Å
4.71 Å
GSFSC Resolution: 4.71Å
CAP
16,704 particles
2D classification
11,509 particles
Ab initio model
Homogenous refinement
(C14 symmetry)
Signal subtraction
of excess tube density
Non-uniform refinement
6.61 Å
7.40 Å
3D classification
10,905
particles
Heterogenous
refinement
4,494
particles
4,063
particles
2,348
particles
Non-uniform refinement
CTF refinement
Non-uniform refinement
6.41 Å
6.34 Å
GSFSC Resolution: 6.34Å
C14 symmetry expansion
119,798
particles
Density subtraction
Focused refinement (trimer)
4.86 Å
3D
classification
53,750
particles
CTF refinement
Focused refinement
GSFSC Resolution: 5.03Å
5.05 Å

Fig. S3. Cryo-EM data processing workflow for the AcMNPV base and cap.

The flow chart for the cryo-EM processing of the AcMNPV cap, generating consensus C14 reconstructions of the base (4.98 Å resolution) and cap (6.34 Å resolution) and focused refinement reconstructions of three subunits for base (4.78 Å and 4.71 Å resolution) and cap (5.05 Å resolution). FSC curves for each of the final reconstructions are shown and represent gold standard FSC curves (GSFSC) where the 0.143 GSFSC cutoff if indicated by the blue horizontal line.

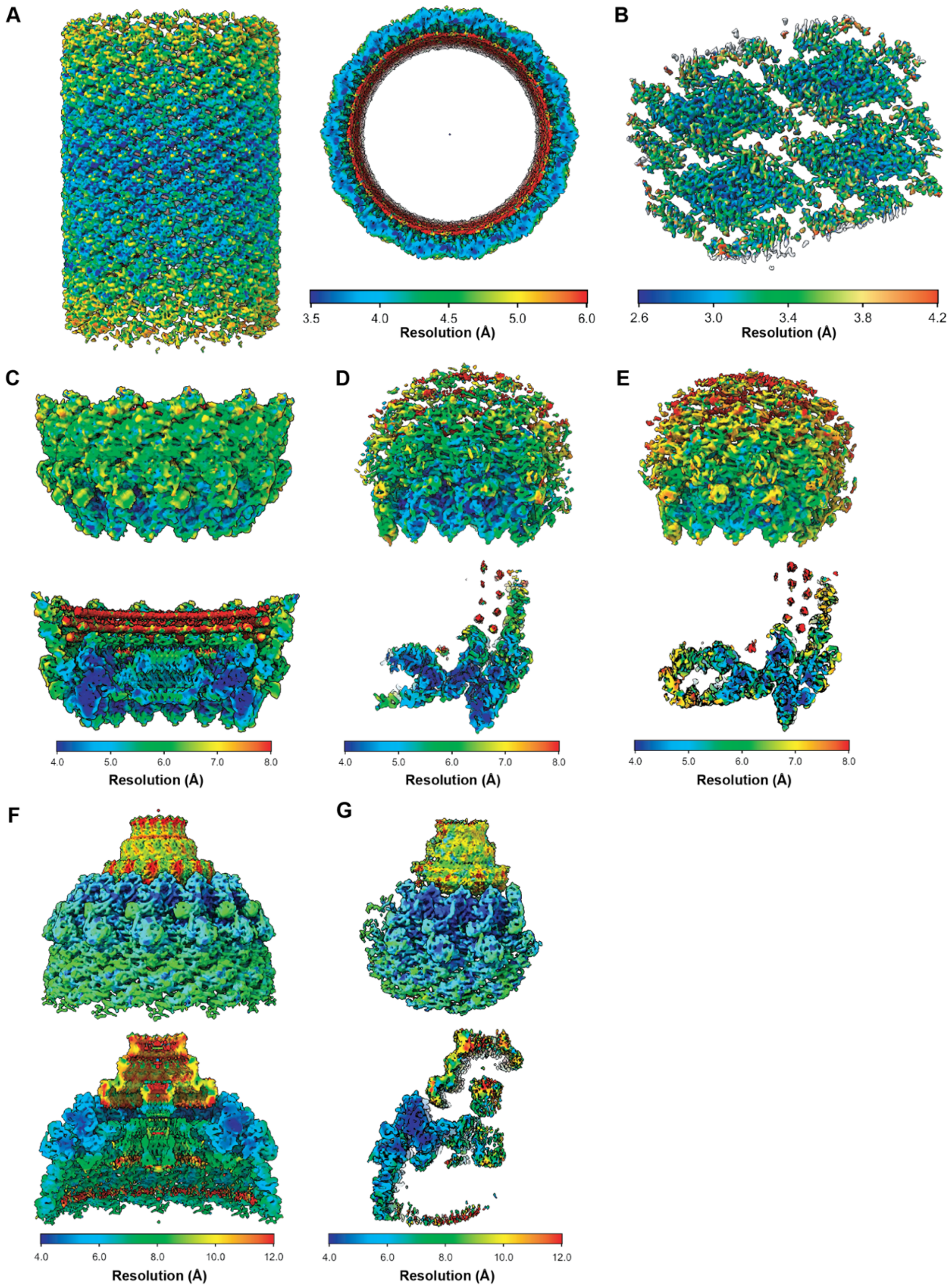

Fig. S4. Local resolution estimates for cryo-EM reconstructions.
Estimated local resolution maps, generated by cryoSPARC, for the consensus refinement of the helical nucleocapsid **(A)**, focused refinement of the helical nucleocapsid **(B)**, consensus refinement of the base **(C)**, focused refinement of the base (**D-E**), consensus refinement of the cap (**F**) and focused refinement of the cap (**G**). Individual reconstructions are coloured based on estimated local resolution as indicated by the associated legend.

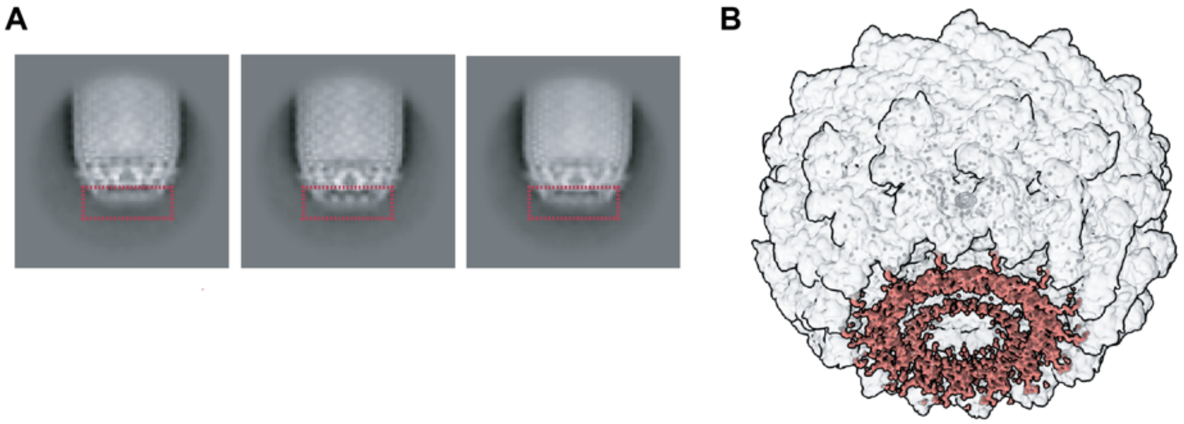

Fig. S5: Example of weak electron density away from the base.
A. Exemplar 2D class averages of the AcMNPV Base reveals the presence of a weak density at the apex of the base as highlighted by the red box. **B.** The C14 consensus reconstruction of the AcMNPV base shown at low contour (contour level of 0.20) showing unstructured, poor density present at the apex of the base (coloured red) that could not be assigned to a particular nucleocapsid protein.

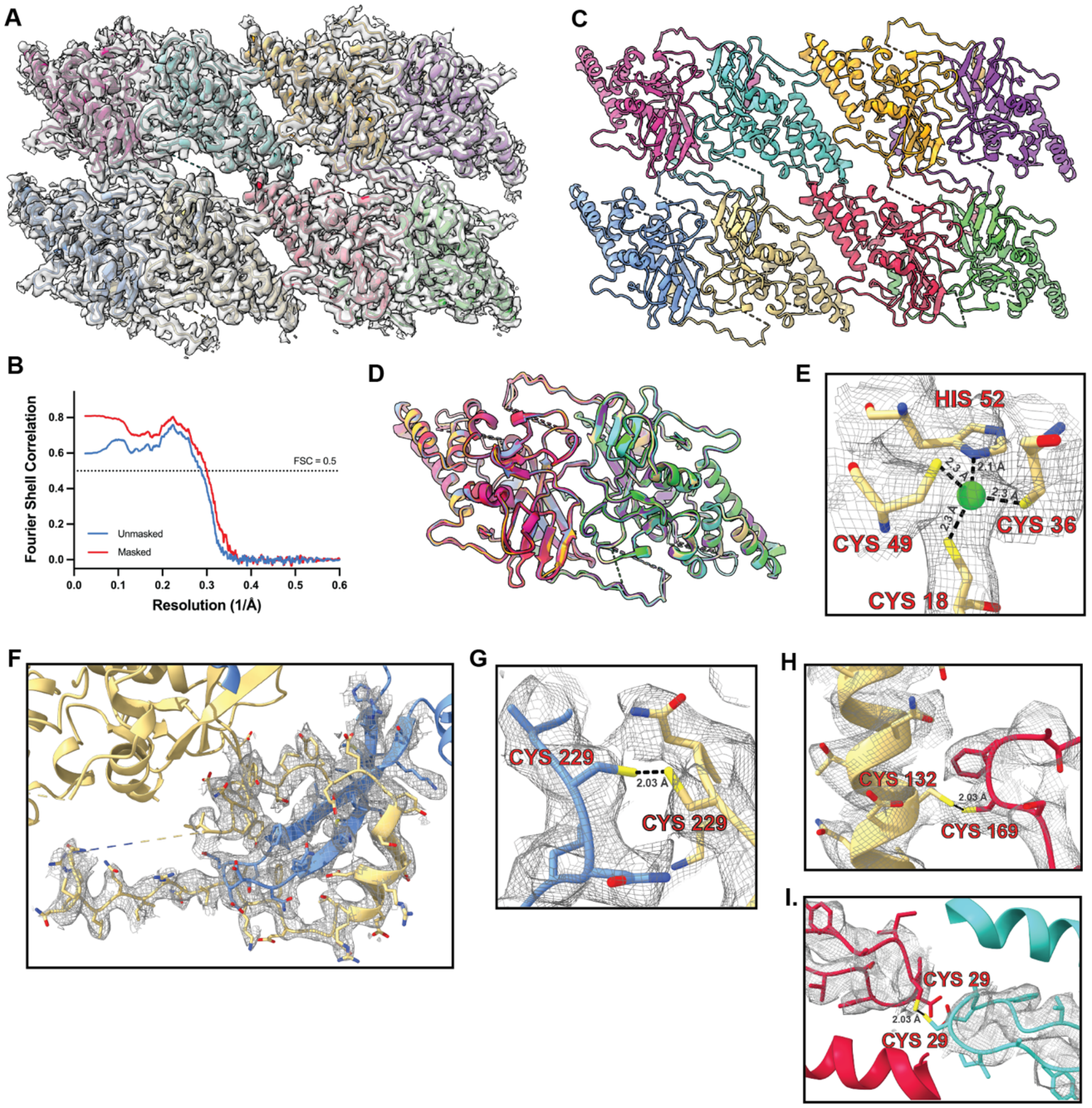

Fig. S6: Model to map fit for the AcMNPV nucleocapsid body.

A. Fit of the refined model (coloured cartoon representation) to the focused refinement map (grey surface representation). **B.** Model-to-map FSC curve calculated with mask (red) and without mask (blue) between the cryo-EM density map and the structural model. **C.** The asymmetric unit of the refined model. **D.** Overlay of each of the four MCP dimers from the refined model. **E.** EM density and structural model for zinc-finger motif (chain A). **F.** EM density and structural model for pin (blue, chain B) and lasso (yellow, chain A) motif. **G.** EM density and structural model for a putative intradimer disulfide (Cys229 – Cys229) between chain A (yellow) and chain B (blue). **H.** EM density and structural model for a putative interdimer disulfide between Cys132 (yellow, chain A) and Cys169 (red, chain G). **I.** EM density and structural model for the potential interstrand disulfide between Cys29 (red, chain G) and Cys29 (red, chain M).

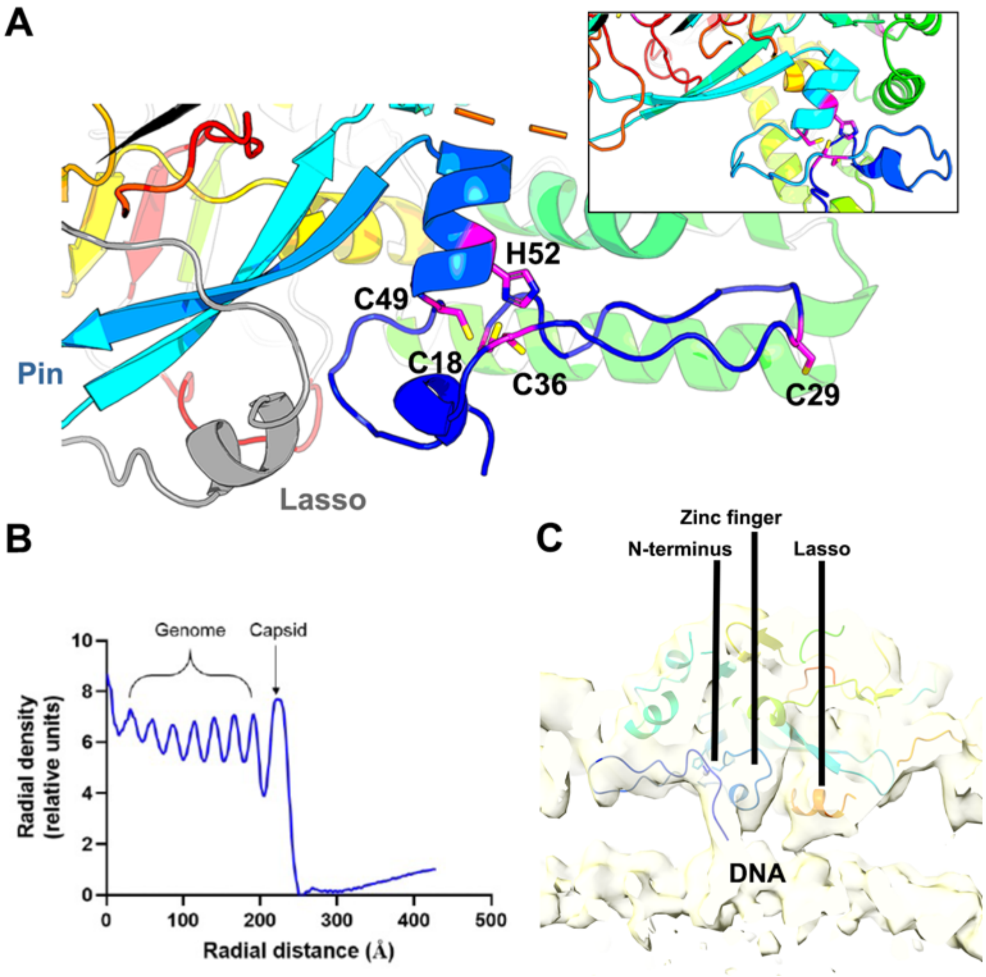

Fig. S7: The MCP zinc-finger and contacts between the genomic DNA and the MCP. **A.** Zinc finger in the MCP protein (residues in magenta). The CCCH motif coordinating the putative Zn^{2+} molecule consists of two amino terminal cysteines (Cys18, Cys36) separated by a two-strand extension placing Cys29 next to a neighboring dimer. The other two zinc ligands are at the N-terminus of a two-turn helix (Cys49, His52). Inset: same view of the Alphafold2 model of the *Tipula oleracea* nudivirus MCP. **B.** Radial density in a rotationally averaged map of the tube as a function of the radial distance from the virion centre. **C.** A dimer of the MCP is shown in a cartoon representation colored blue-to-red from the N- to the C-termini. The electron density map is shown as a semi-transparent surface colored in brown. Key points of contacts are labelled.

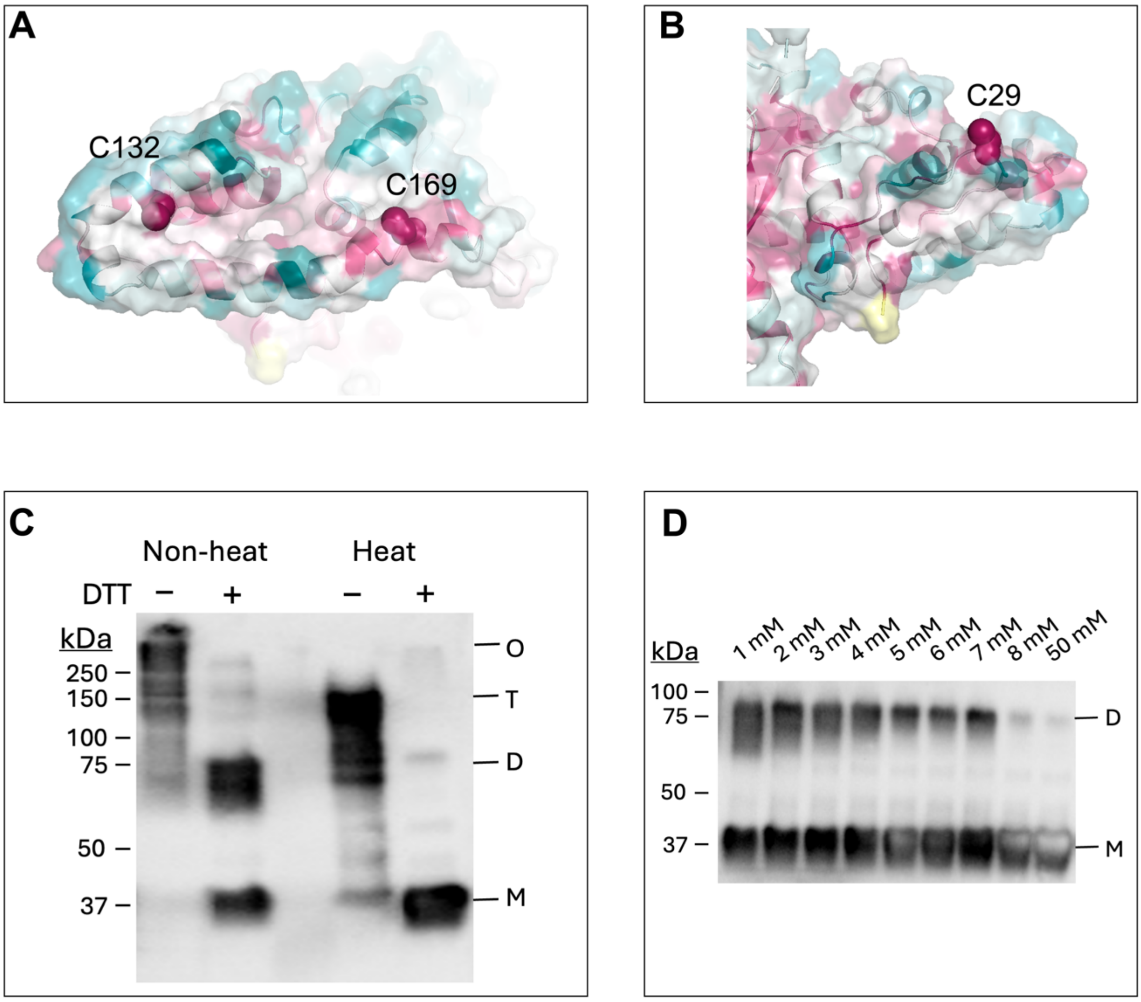

Fig. S8: The nucleocapsid contains inter-molecular disulfide bonds crosslinking the MCP subunits.

A, B. Sequence conservation mapped onto the structure of the MCP as estimated by Consurf for 55 non-redundant sequences covering the *Baculoviridae* family. The views are centered on Cys132/Cys169 (A) and Cys29 (B), which contribute to intra- and inter-strand contacts, respectively (cf. Fig 2). The cyan-white-magenta color scheme indicates residues from the least to the most conserved. **C.** Disassembly of the AcMNPV BV particles was analyzed by Western blot with and without DTT and heat treatment. Oligomeric MCPs larger than the tetramer were detected in absence of DTT and heat treatment. In reducing conditions, only monomeric and/or dimeric MCPs were detected. **D.** Western blot analysis of DTT-dependent disassembly of AcMNPV nucleocapsid. Purified BV nucleocapsids were incubated in increasing amount of DTT prior to sample preparation in the SDS-PAGE loading dye (no additional heat or reducing agent applied). M = monomers, D = Dimers, T = Tetramers, and O = Oligomers. Antibody: monoclonal anti-VP39 (kindly supplied by Dr Taro Ohkawa, University of California Berkeley).

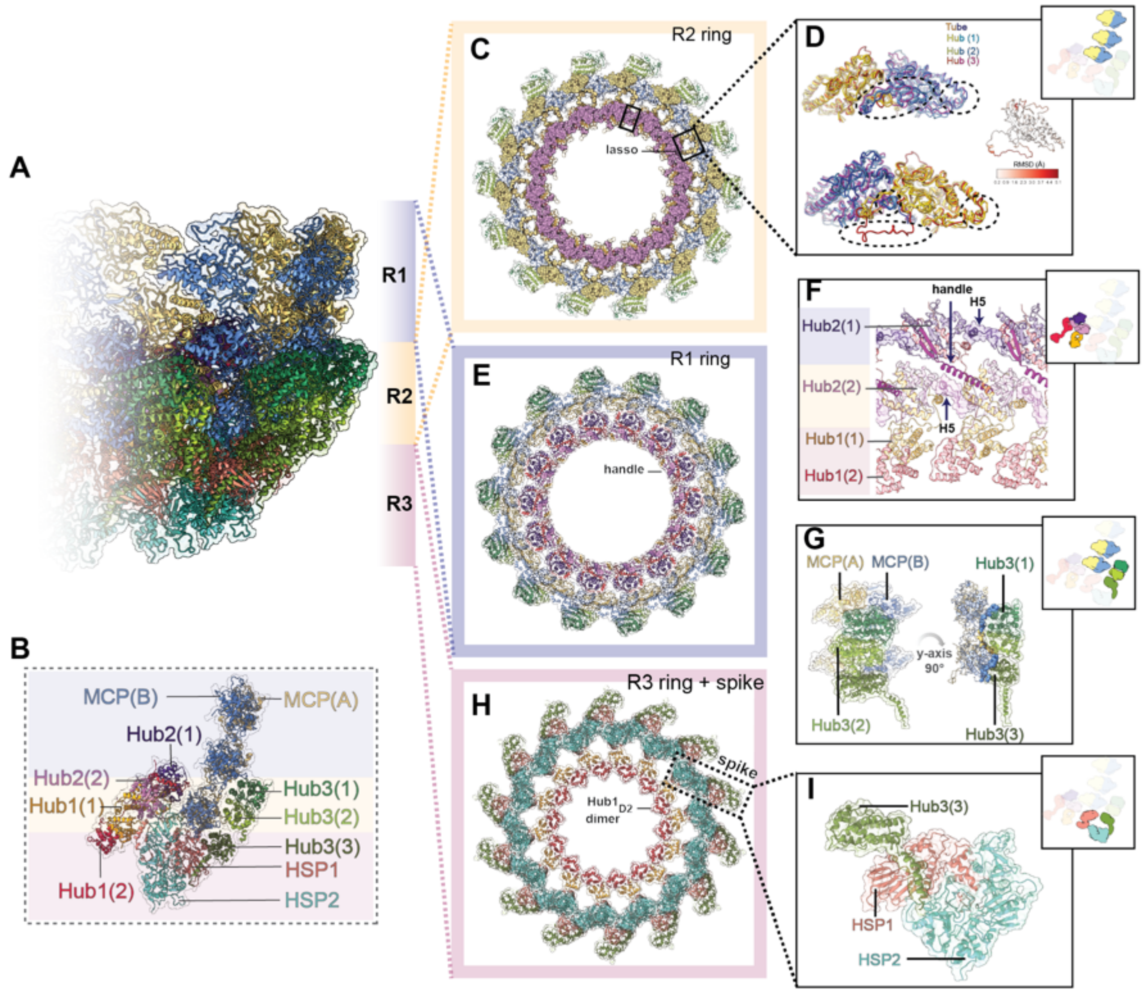

Fig. S9: A 126-protein hub forms the core of the viral base and cap.

A. Model of the hub, built into the cryo-EM reconstruction of the base, shown as cartoon representation and coloured per component. The three rings of Hub1/Hub2 (C42/EC27) are labelled as R1 (blue), R2 (yellow) and R3 (maroon). **B.** Asymmetric unit of the hub. **C.** Clipped view of the R2 ring of the hub, coloured as in panel b. A single MCP subunit (shown as yellow spheres) has a flipped lasso which creates the interaction with Hub2 (shown as pink spheres) that anchors the tube to the hub. **D.** Quasi-equivalence of the MCP. Left, overlay of the modelled MCP dimers from the tube (orange, blue) and rings of the hub (1 - yellow, sky blue; 2 - light green, blue; 3 - red, purple) reveals loops that differ significantly, as highlighted by dashed circles. Right, single molecule of MCP (chain A, tube model) coloured by RMSD (α-carbon) of all MCP chains. **E.** Clipped view of the R1 ring of the hub, coloured as per b. **F.** The [Hub1/Hub2]$_2$ heterotetramer has an asymmetrical organisation based on stacked Hub1$_{D3}$/Hub2 complexes forming R1 and R2, and a Hub1$_{D2}$ homodimer forming R3. The Hub2^{R2} handle and H5 helices in both Hub2^{R1} and Hub2^{R2} that form domain-swapped interactions within the hub are labelled. **G.** Arrangement of the three copies of Hub3 (VP80) in the asymmetric unit that interact with the last two MCP rings. A single Hub3 molecule, Hub3 (3), has a unique flipped α-helix that provides attachment for the spike. **H.** Clipped view of the R3 ring and spike of the hub, coloured as per b. **I.** The asymmetric unit of the distal spike of the hub, formed by Hub3 (3) and a tight heterodimer of HSP1 (AC109) and HSP2 (AC142).

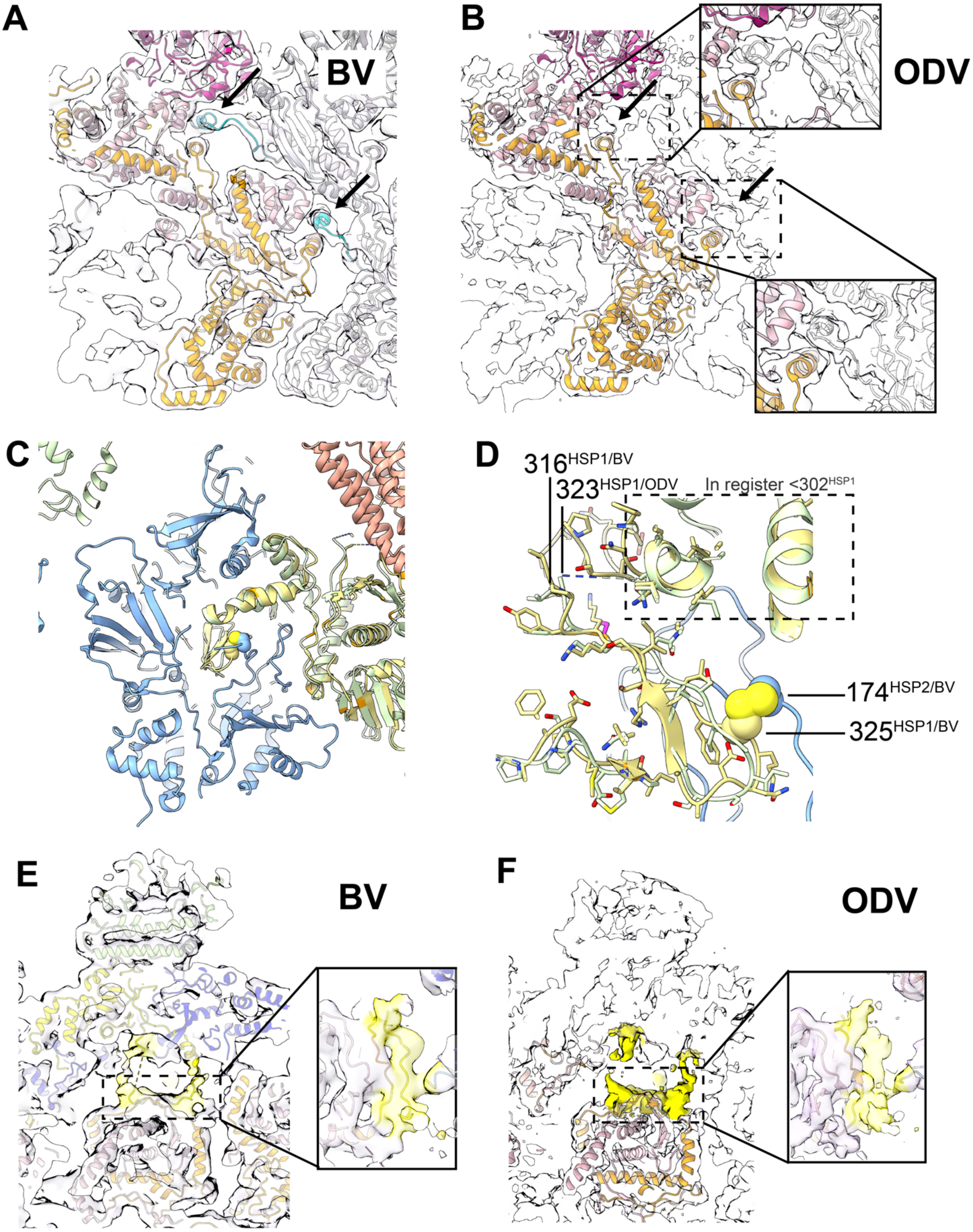

Fig. S10: Features of the BV nucleocapsid differing from the ODV model of the hub.

A, B The Hub1-Hub2 domain swap was not modelled in the ODV structure (PDB ID: 8i8b) but is likely to be present in both the BV (A) and ODV (B) hubs. A heterotetramer of [Hub1-Hub2]$_2$ is shown as ribbon colored in orange, pink and magenta for Hub1, Hub2 and BCP. The neighboring tetramer is colored in white except for the domain swap of Hub2's helix H5 shown

in cyan (arrows). The semi-transparent surface corresponds to the focused reconstructions of the base for the BV infectious particle (this study) and ODV nucleocapsid (EMDB 35246). The insets show zoomed views of the published reconstruction of ODV with the ribbon of the neighboring BV Hub 2 in white. **C** Ribbon representation of the base zoomed on the HSP1 (yellow/green for BV and ODV, respectively) and HSP2. In BV, Cys325^{HSP1} is near Cys174^{HSP2} (both residues shown as spheres). **D,** HSP1 and HSP2 are shown as ribbon with sticks for the side chains of HSP1 and colored as in C. Our modelling of the BV HSP2 differs from the published ODV HSP1 (PDB ID: 8i8b) due to a register shift between residues 316-350. In both reconstructions, the density is poor in this area so that differences could be real or due to a modelling mistake. **E,F** View of the base focused on the lasso anchoring the MCP layer to the Hub1-Hub2 ring. The color scheme is the same as Fig. 1. The semi-transparent surface corresponds to the focused reconstructions of the BV (E) and ODV (F) bases as in panels A,B. The lasso anchors the MCP onto the hub in both the BV and ODV. The electron density map is colored in yellow within 8 Å of the BV lasso in both the E and F panels. No model was built in this density in the ODV structure (PDB ID: 8i8b). Insets: zoomed view from the top compared to the main panel (90º rotation).

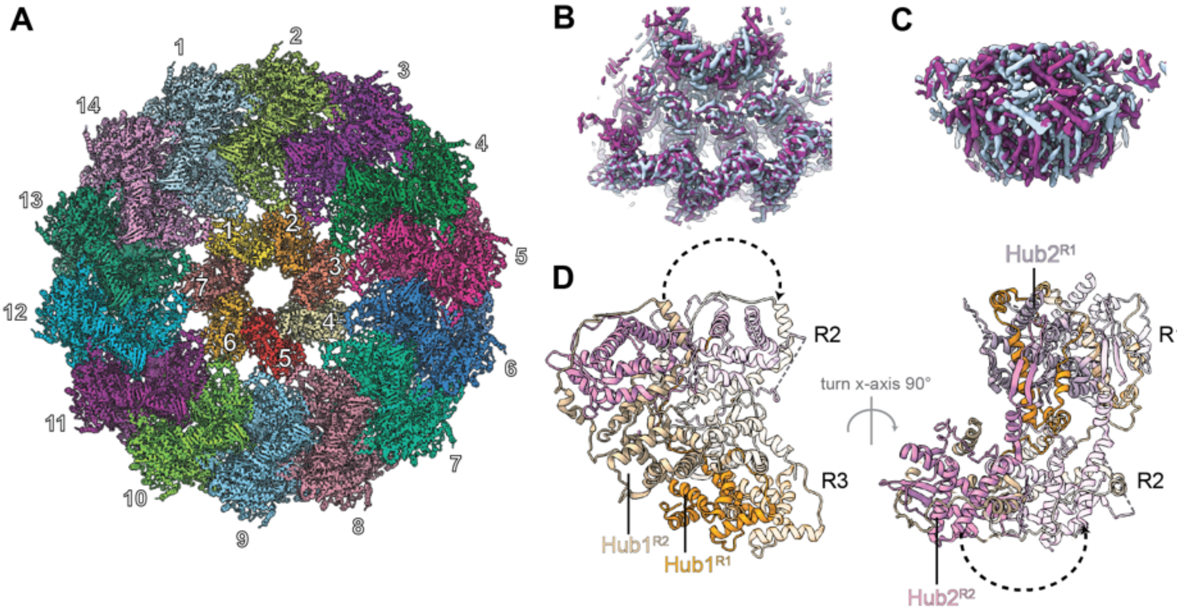

Figure S11: Symmetry mismatch and rotation between the hub and the plug in the base. **A.** Bottom view of the complete model of the AcMNPV base, colored per protomeric unit of the hub (exterior ring, C14 symmetry) and the plug (interior ring, C7 symmetry). **B.** Bottom superimposition of the two electron density maps resulting from focused reconstructions of different classes of the base (blue and purple). These reconstructions comprise 3 asymmetric units of the hub (3 copies of MCP_6-$[Hub1/Hub2]_2$-$Hub3_3$-HSP1/HSP2-BCP) and of the plug ($[Hub1/Hub2]_2$). The reconstructions reveal consistent placement of the hub but distinctly different rotations of the plug with respect to the hub. **C.** As in b, but side-view of the plug inner rings for the two reconstructions. **D.** Orthogonal views of superimposed models for the Hub1 (orange and beige) and Hub2 (pink and light pink) components of the plug built into the two different focused reconstructions of the base (cf. supplementary text).

A.

	Protein complex	CAPRI measures			DockQ
		Fnat	iRMS	LRMS	
Acceptable	Hub1 + BANPF	0.968	7.582	27.85	0.364
Incorrect	BANR + BANPF	0	12.017	58.258	0.012
Medium	Hub1D1+ BANPR	0.925	1.755	9.667	0.594
Medium	Hub1D1+ N-ter BANPF	0.971	0.867	12.729	0.676

Criteria	Reference: 2016_PLos One_DockQ: A Quality Measure for Protein-Protein Docking Models
DockQ < 0.23	Incorrect (Fnat < 0.1 or (LRMS > 10 and iRMS > 4.0))
DockQ < 0.5	Acceptable ((Fnat ≥ 0.1 and Fnat < 0.3) and (LRMS ≤ 10.0 or iRMS ≤ 4.0) or (Fnat ≥ 0.3 and LRMS > 5.0 and iRMS > 2.0)
DockQ < 0.75	Medium ((Fnat ≥ 0.3 and Fnat < 0.5) and (LRMS ≤ 5.0 or iRMS ≤ 2.0) or (Fnat ≥ 0.5 and LRMS > 1.0 and iRMS > 1.0))
DockQ > 0.75	High (Fnat ≥ 0.5 and (LRMS ≤ 1.0 or iRMS ≤ 1.0))

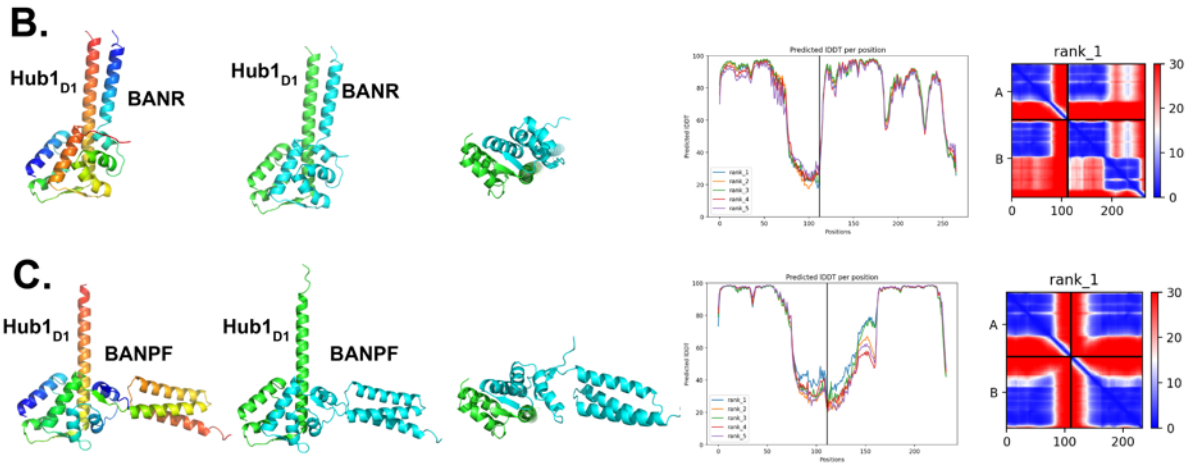

Figure S12: Modelling of the base components involved in actin-tail polymerisation.
A. The Hub1 (C42), BANPF (p78/83) and BANR (AC102) proteins were modelled as a complex using AlphaFold2 and subjected to analysis with DockQ for validation. **B, C.** Cartoon representations of the putative $Hub1_{D1}$-BANR (B) and $Hub1_{D1}$-BANPF (N-terminal domain) (C) complexes shown in a blue-red spectrum from N- to C-termini or as orthogonal views colored per molecule as labelled. The per-residue pLDDT and PAE plots support a complex of the globular domains in keeping with panel A.

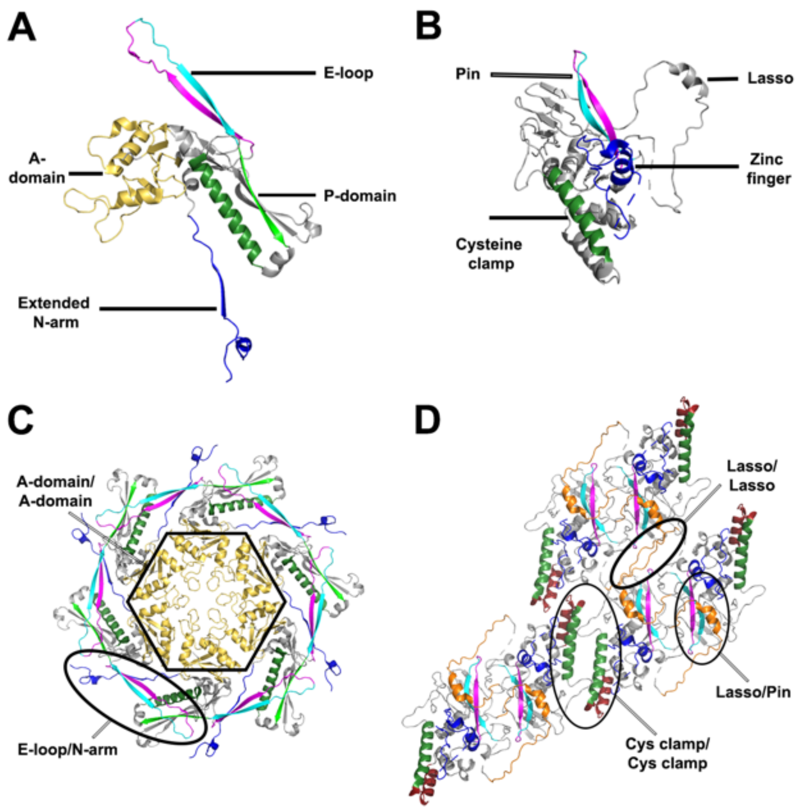

Figure S13: The MCP is a new fold, not homologous to the HK97 fold.
Cartoon representation of the HK97 main capsid protein (PDB 1OHG) (A) and baculovirus MCP proteins (B). The structures were aligned manually to superpose the HK97 E-loop (magenta/cyan) with the MCP Pin, and the spine helix of the P-domain in HK97 with the first helix of the cysteine clamp in the MCP. **(A)** The HK97-fold is characterised by four structural elements: (1) an extended N-arm with alpha-helical content (blue); (2) an E-loop consisting of a two-stranded beta-sheet (magenta/cyan); (3) a P-domain characterised by a long beta-sheet (bright green) in continuation of the E-loop and a parallel spine helix (dark green); (4) an A-domain with a beta-hinge domain decorated by short helices (yellow). **(B)** The baculovirus MCP has a two-stranded beta-sheet (cyan/magenta) and a parallel 5-turn helix (dark green) that were compared to the E-loop and the spine helix by Jia et al (22). We find that the beta-strand differs from the HK97 E-loop in its flipped orientation, with beta1 instead of beta2 exposed on the protein surface, and is not extended by a central beta-strand running along the length of the protein as in HK97. We also find that the 5-turn helix is in a structural context that does match the HK97 P-domain due to the lack of a flanking, long beta-sheet. The extended N-arm and wedge-shaped A-domain are absent in MCP. Instead, the N-terminus of MCP (blue) contains a conserved Zinc-finger motif and the remaining of the protein forms a compact core lacking the beta hinge-like topology of the A-domain. **(C-D)** Representation of assembled capsids. An hexon of the icosahedral capsid is represented for the HK97-fold MCP (c). For the baculovirus MCP, a central dimer (right) and its two neigbouring dimers within the helical tube show the inter- (top right) and intra-strand (bottom left dimer) interfaces (d). Key interactions are labelled. The color scheme is the same the (A-B) panels except for the lasso (orange) and the cysteine clamp (green/red).

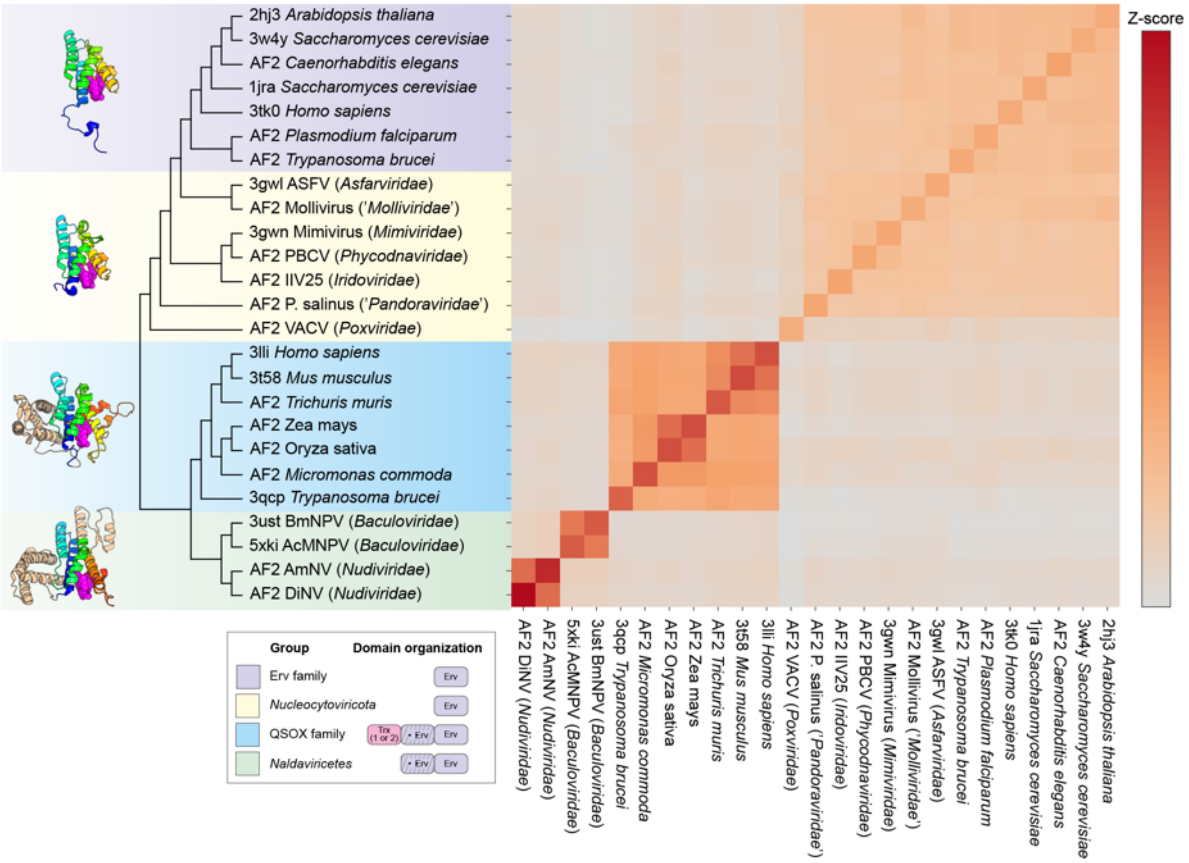

Figure S14: Structural relationships between cellular and viral sulfhydryl oxidases. Matrix and cluster dendrogram were constructed based on the pairwise Z-score comparisons calculated using DALI. Different protein groups are highlighted with different background colors on the dendrogram: Erv family proteins from cellular proteins, pink; sulfhydryl oxidases from *Nucleocytoviricota*, yellow; Quiescin family sulfhydryl oxidases (QSOX) from cellular organisms, blue; sulfhydryl oxidases from baculo-like viruses, green. Inset shows the domain organizations for proteins in the four highlighted groups of sulfhydryl oxidases. The color scale on the right indicates the corresponding Z-scores. Abbreviations: ASFV, African swine fever virus; PBCV, *Paramecium bursaria* Chlorella virus FR483 (YP_001426300); IIV25, invertebrate iridovirus 25 (YP_009010549); *P. salinus*, pandoravirus salinus (YP_008437137); Mollivirus, *Mollivirus sibericum* (YP_009165401); VACV, vaccinia virus; BmNPV, *Bombyx mori* nuclear polyhedrosis virus; AcMNPV, *Autographa californica* multiple nucleopolyhedrosis virus; AmNV, *Apis mellifera* nudivirus (WOJ45379); DiNV, *Drosophila innubila* nudivirus (YP_009553151); AF2, AlphaFold2 model. For experimentally determined structures, the corresponding PDB accession numbers are indicated next to the corresponding species names. Representative structures (PDB ID 3TK0, 3GWN, 311I, 3PK0) are shown on the left with the Erv domain colored as a rainbow and unique domains in the baculovirus and QSOX proteins in light brown. The FAD molecule is shown as magenta spheres.

A

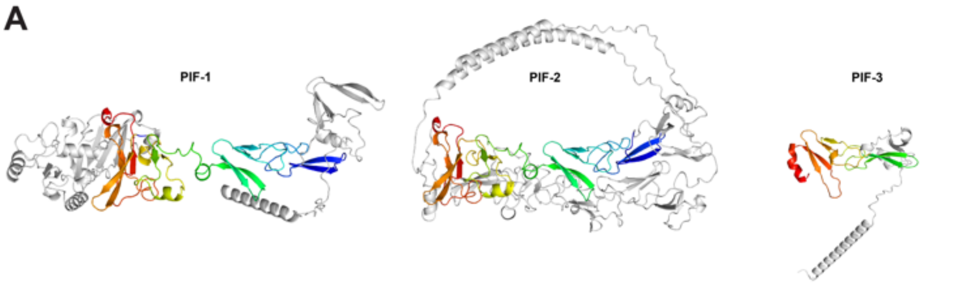

B

	Protein complex	CAPRI measures			DockQ
		Fnat	iRMS	LRMS	
Acceptable	PIF1 + PIF2	0.884	5.626	11.968	0.429
Acceptable	PIF1-2 + PIF3	0.713	14.078	10.715	0.333
Acceptable	PIF1-2-3 + PIF4	0.682	8.536	19.782	0.289
Acceptable	PIF0 + PIF1-2	0.742	8.111	6.87	0.46

Criteria	Reference: 2016_PLos One_DockQ: A Quality Measure for Protein-Protein Docking Models
DockQ < 0.23	Incorrect (Fnat < 0.1 or (LRMS > 10 and iRMS > 4.0))
DockQ < 0.5	Acceptable ((Fnat ≥ 0.1 and Fnat < 0.3) and (LRMS ≤ 10.0 or iRMS ≤ 4.0) or (Fnat ≥ 0.3 and LRMS > 5.0 and iRMS > 2.0)
DockQ < 0.75	Medium ((Fnat ≥ 0.3 and Fnat < 0.5) and (LRMS ≤ 5.0 or iRMS ≤ 2.0) or (Fnat ≥ 0.5 and LRMS > 1.0 and iRMS > 1.0))
DockQ > 0.75	High (Fnat ≥ 0.5 and (LRMS ≤ 1.0 or iRMS ≤ 1.0))

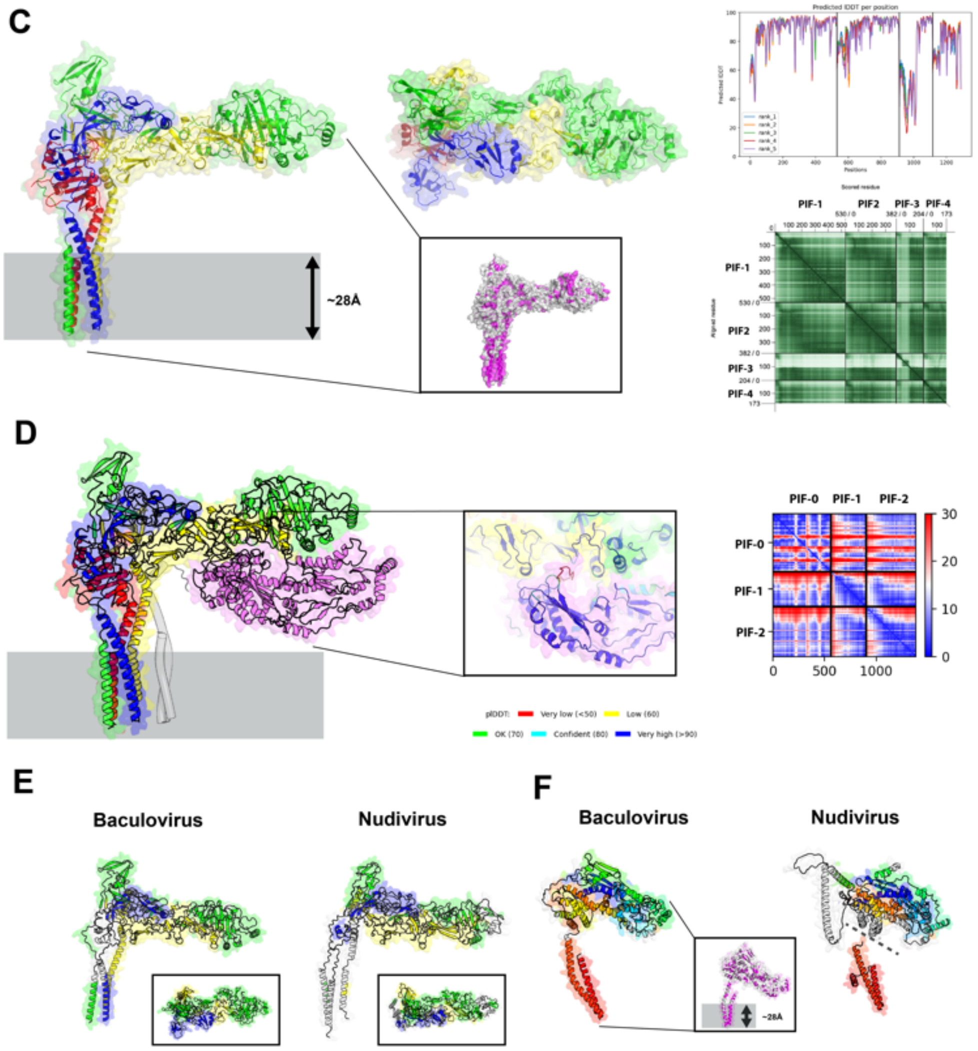

Figure S15: The virulence module is characterised by a complex of the PIF proteins on the surface of ODVs.

A, HHPred profile analysis identifies PIF proteins 1, 2 and 3 as homologous, which suggests they emerged by duplication of an ancestral gene. Structural homology is confirmed by Foldseek as shown in the cartoon representation of the PIF proteins. Domains with significant structural similarity are highlighted in a blue-red spectrum from N- to C-termini. **B.** The PIF-0, PIF-1, PIF-2, PIF-3 and PIF-4 proteins were modelled as complexes using AlphaFold2 in various combinations and subjected to analysis with DockQ for validation. **C. Left and middle:** Orthogonal cartoon representations of the putative PIF-1,2,3,4 complex (green, yellow, blue and red, respectively). The N-terminal alpha-helices of the four proteins are likely to be embedded in the viral membrane represented in grey. The inset shows hydrophobic regions (magenta) in the putative complex which is represented in grey. **Right:** The per-residue pLDDT and PAE plots support the existence of the quaternary complex in keeping with panel B. **D. Left:** The entire complex could not be modelled due to computational limitations. This panel presents a composite model of PIF-1,2,3,4 as in c and PIF-0 as modelled by AlphaFold2 in a PIF-0,1,2 complex (interacting domains only). The position of the PIF-0 C-terminal helices is schematised by grey cylinders to indicate their consistent location in modelling attempts despite very low reliability score. The inset shows the interface colored by pLDDT score. **Right:** PAE plot. **E.** The AcMNPV (left) and *Tipula oleracea* nudivirus (right) PIF-1,2,3 complex are shown with the same color scheme as panel c and a top view in the insets. **F.** The AcMNPV (left) and Tipula oleracea nudivirus (right) PIF-0. The C-terminal helices (orange, 675-763) were aligned with the AcMNPV PIF-0 independently from the rest of the protein. The orientation between the two domains is poorly defined in AcMPNV and the nudivirus as assessed from the PAE plots. The inset shows the molecular surface of AcMNPV PIF-0 (grey) with hydrophobic regions highlighted in magenta.

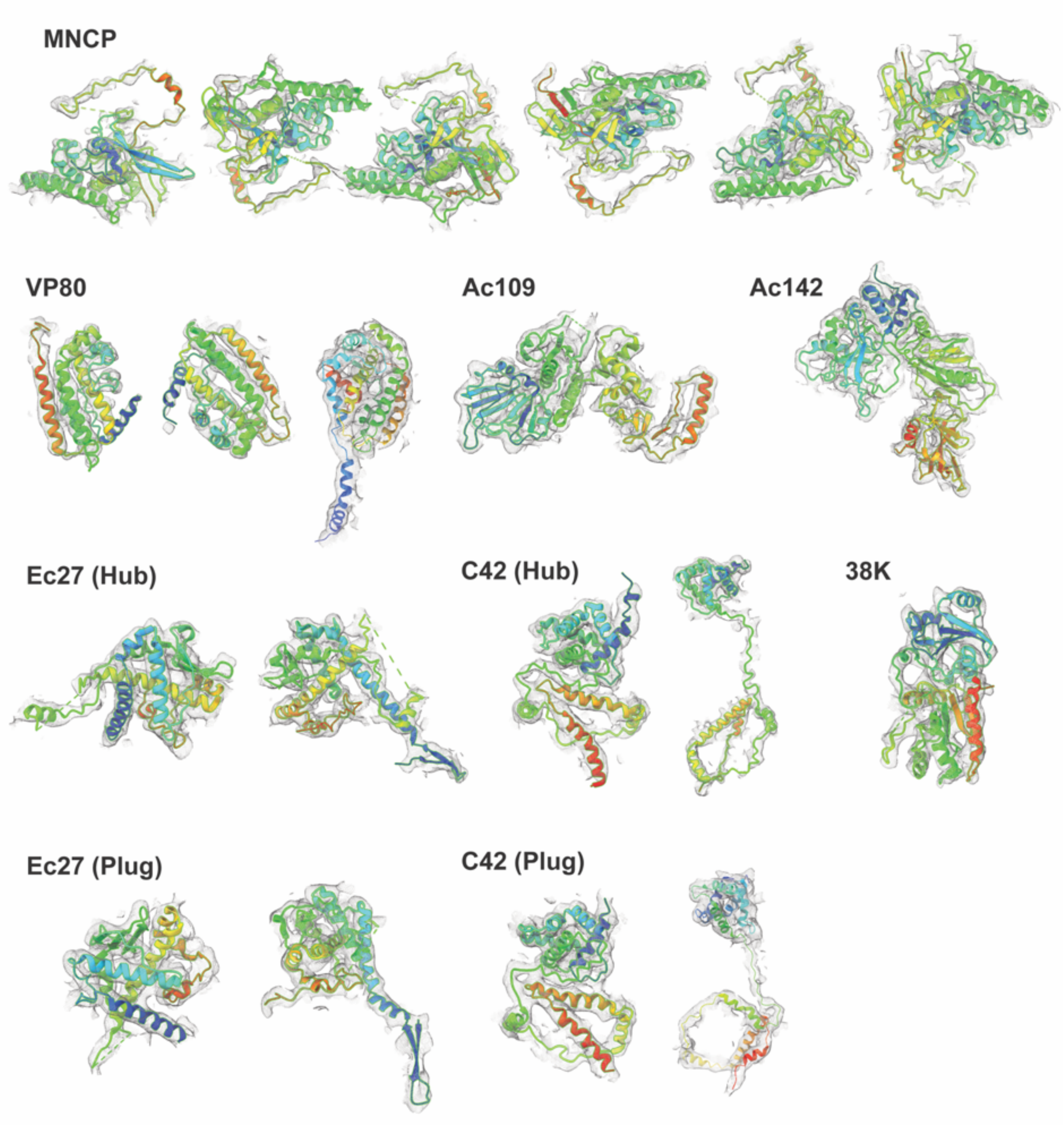

Fig. S16: Placement of assigned AcMNPV assigned components in electron density of Base reconstruction.

Superimposition of the refined models built into the focused reconstruction of the base, shown in cartoon representation and rainbow coloured from N-terminus (blue) to C-terminus (red), and corresponding map density, shown as grey mesh.

Table S1: Cryo-EM data collection, refinement and validation statistics for helical nucleocapsid body

	Tomography	Single particle	
		Body	Body (Local reconstruction)
**Data collection and image processing**			
Microscope	FEI Titan Krios	FEI Titan Krios	
Camera	Gatan K2	Gatan K2	
Magnification	81,000	105,000	
Electron energy (kV)	300	300	
Total dose ($e^-/Å^2$)	98.4	47.3	
Exposure time (s)	1 (per tilt)	14	
Frames	5 (per tilt)	70	
Tilt range	± 60	-	
Tilt increment	3°	-	
Defocus range (µm)	2.0 – 3.5	0.4 – 1.2	
Pixel size (Å)	1.82	0.705	
Total number of micrographs		1849	
Symmetry imposed		C14 + helical	C1
Helical twist		-18.56°	-
Helical rise (Å)		43.22	-
Initial number of particles		77,356	2,165,694
Final number of particles		63,595	1,002,951
Map resolution (Å)		4.24	3.06
FSC threshold		0.143	0.143
EMDB ID		EMD-43585	EMD-43586
**Model building and refinement**			
Initial model used			VP39 AF2 model
No. non-hydrogen atoms			18,890
Protein residues			2329
Ligands			ZN: 8
Model-to-map fit			
CC (mask)			0.75
Mean CC ligands			0.69
R.m.s deviations			
Bond lengths (Å)			0.002
Bond angles (°)			0.437
Molprobity score			1.86
Clashscore			10.58
Ramachandran plot			
Favoured (%)			95.49
Allowed (%)			4.51
Outliers (%)			0.0
Rotamer outliers (%)			0.05
Cβ outliers (%)			0.00
PDB ID			8VWH

Table S2: Cryo-EM data collection, refinement and validation statistics for nucleocapsid base and cap complexes

	Base	Base (Local reconstruction) (1)	Base (Local reconstruction) (2)	Cap	Cap (Local Reconstruction)
**Data collection and image processing**					
**Microscope**			FEI Titan Krios		
**Camera**			Gatan K3		
**Magnification**			64,000		
**Electron energy (kV)**			300		
**Total dose (e^-/Å^2)**			50.0		
**Exposure time (s)**			8.44		
**Frames**			50		
**Defocus range (µm)**			0.8 – 1.4		
**Pixel size (Å)**			1.41		
**Total number of micrographs**			11,439		
**Symmetry imposed**	C14	C1	C1	C14	C1
**Initial number of particles**	20,375	129,556	129,556	16,704	119,798
**Final number of particles**	9,254	53,750	54,429	8,557	53,750
**Map resolution (Å)**	4.98	4.71	4.78	6.34	5.05
**FSC threshold**	0.143	0.143	0.143	0.143	0.143
**EMDB ID**	EMD-43587	EMD-43588	EMD-43589	EMD-43590	EMD-43591
**Model building and refinement**					
**Initial model used**		MCP refined model, AF2 models of Hub1, Hub2, Hub3, HSP1, HSP2, BCP			
**No. non-hydrogen atoms**		156,195	156,195		
**Protein residues**		9,602	9,602		
**Ligands**		ZN: 12	ZN: 12		
**Model resolution (Å) (0.143/0.5)**		4.8/7.0	4.7/7.0		
**Model-to-map fit**					
**CC (mask)**		0.61	0.64		
**CC (volume)**		0.65	0.67		
**CC (main chain)**		0.67	0.69		
**Mean CC ligands**		0.57	0.61		
**R.m.s deviations**					
**Bond lengths (Å)**		0.003	0.005		
**Bond angles (°)**		0.751	0.893		
**Molprobity score**		1.82	2.40		
**Clashscore**		5.14	7.97		
**Ramachandran plot**					
**Favoured (%)**		90.13	89.55		
**Allowed (%)**		9.80	10.28		
**Outliers (%)**		0.06	0.17		
**Rotamer outliers (%)**		0.00	3.36		
**Cβ outliers (%)**		0.00	0.00		
**PDB ID**		8VWI	8VWJ		

Table S3 – Sequence and structure-based analysis of AcMNPV nucleocapsid and hallmark proteins. The table summarizes data presented in the extended data spreadsheet (Data S1). Rows colored in green do not have detectable homologues. Rows colored in brown, cyan, yellow, pink or grey correspond to proteins where the closest homologue is of eukaryotic, prokaryotic, varidnaviria, duplodnaviria or riboviria origin, respectively. The use of two colors indicates when the origin could not be resolved unambiguously.

[#] Gene conservation. A: all Naldaviricetes, +++: all but nimaviridae; ++: baculo- and nudiviridae; *: baculovirus; - : not a baculovirus core gene.

BANPF: baculovirus actin nucleation promoting factor; BANR: baculovirus actin nucleation regulator; BCP: baculovirus CTD phosphatase; PLP: protamine-like protein; Hub: hub proteins; HSP: hub spike proteins; MCP: major capsid protein; BSOX: baculovirus sulfhydryl oxidase; LEF: late expression factor; PIF: *per os* infectivity factor.

Green: no significant homologue outside of Naldaviricetes
Brown: closest homologue in eukarya
Cyan: closest homologue in prokarya
yellow: closest homologue in varidnaviria
pink: closest homologue in duplodnaviria
blank: other viruses (Riboviria)

Gene	Protein	Conservation[#]	Baculovirus	Closest Homologue	
**Structure**					
**Ac9**	BANPF (P78/83)	-			
**Ac23**	F protein (Fusion)	-	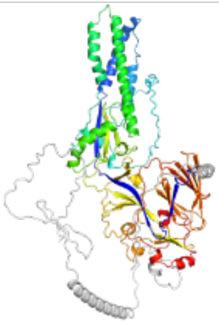	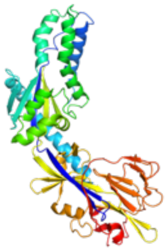	F-glycoprotein from Human Metapneumovirus (PDB code: 7UR4)
**Ac54**	VP1054	*			
**Ac77**	Very late expression factor 1 (VLF-1)	++	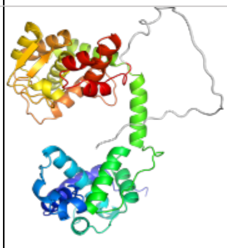	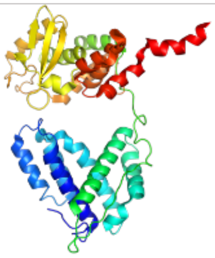	Recombinase from *Helicobacter pylori* (PDB code: 5JJV)
**Ac89**	MCP (VP39)	++			
**Ac98**	BCP (38K)	++	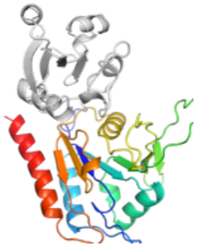	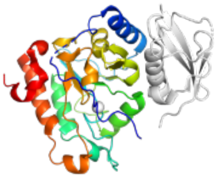	UBLCP1 from *Drosophila melanogaster* (PDB code: 3SHQ)
**Ac100**	PLP (p6.9)	++			
**Ac101**	Hub1 (ODV-C42)	*			
**Ac102**	BANR (p12)	-			
**Ac104**	Hub 3 (VP80)	-			
**Ac109**	HSP1 (AC109)	*			
**Ac128**	GP64 fusion protein (GP67)	-	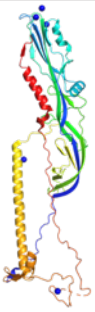	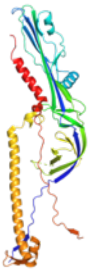	Envelop glycoprotein from Thogotovirus (PDB code: 5XEA)
**Ac142**	HSP2	*			
**Ac143**	ODV-E18	*			
**Ac144**	Hub 2 (ODV-EC27)	*			

**Morphogenesis**					
**Ac53**	AC53	*			
**Ac66**	AC66	*			
**Ac78**	AC78	*			
**Ac80**	GP41	*			
**Ac92**	BSOX (p33)	A	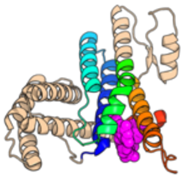	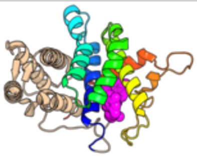	Human sulfhydryl oxidase (PDB code: 3LLI)
**Ac93**	AC93	*			
**Ac94**	Virus envelope protein E25	*	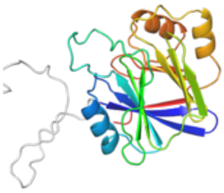	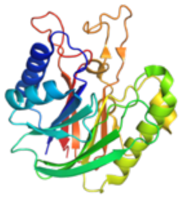	Tyrosyl-DNA phosphodiesterase II from *Mus musculus* (PDB code: 5J3Z)
**Ac103**	p48	*			
**Virulence**					
**Ac138 P74**	Per os infectivity factor 0 (PIF-0)	A			
**PIF-1**	PIF-1	A			
**PIF-2**	PIF-2 (ORF 8/6)	A			
**Ac115 PIF-3**	PIF-3	A			
**Ac96 PIF-4**	PIF-4	+++			
**Ac148**	PIF-5 (ODV-E56)	A			
**Ac68**	PIF-6	++			
**Ac110 PIF-7**	PIF-7	+			
**Ac83**	PIF-8 (VP91, p95)	++			
**Others**					
**Ac81**	Baculovirus Disulfide Isomerase	+++			
**Ac133, AN**	Alkaline nuclease (AN)	*	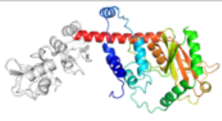	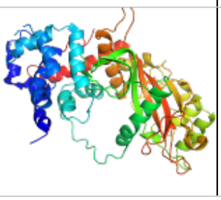	Alkaline nuclease from Human Herpesvirus 4 strain B95-8 (PDB code: 2W4B)

Information Processing

Ac6	Primase-associated factor, Late expression factor 2 (LEF-2)	+	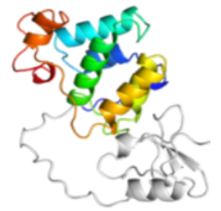	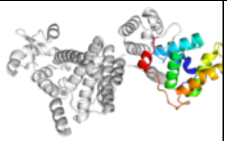	DNA polymerase from *Saccharomyces cerevisiae* (PDB code: 8FOC)
Ac14	DNA primase, (LEF-1)	+	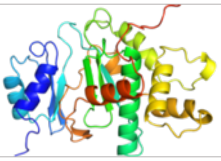	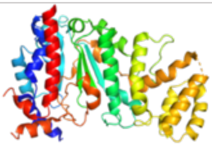	DNA polymerase from *Xenopus laevis* (PDB code: 8G9F)
Ac40	RNA polymerase subunit (p47)	++			
Ac50	DNA-directed RNA polymerase catalytic subunit (LEF-8)	+++	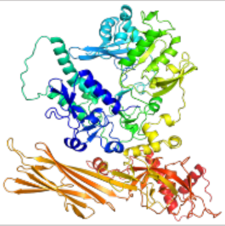	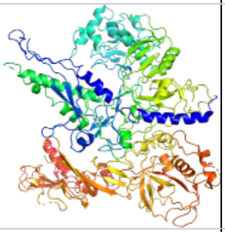	RNA polymerase from *Arabidopsis thaliana* (PDB code: 7EU1)
Ac62, 62l5	DNA-directed RNA polymerase subunit (LEF-9)	+++	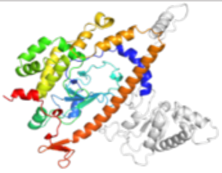	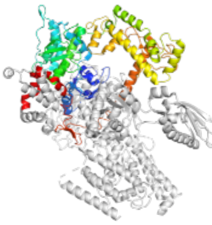	RNA polymerase I from *Schizosaccharo myces pombe* 972h- (PDB code: 7AOE)
Ac65	DNA polymerase	A	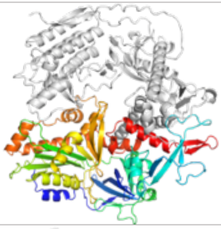	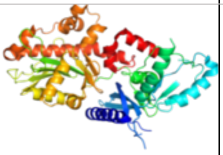	DNA polymerase epsilon from *Saccharomyces cerevisiae* S288C (PDB code: 6S1C)
Ac90	RNA polymerase subunit (LEF-4)	+++	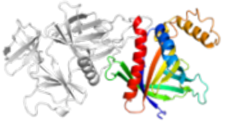	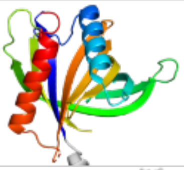	RNA triphosphatase from *T. cruzi* strain BL Brener (PDB code: 6L7V)
Ac95	DNA replication helicase (P143)	+++	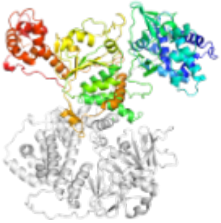	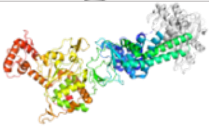	Putative primase C962R from African swine fever virus BA71V (PDB code: 8IQI)
Ac99	LEF-5	+++			

Table S4 – Dynamo alignment parameters used for the baculovirus capsid.

Parameter	Round 1	Round 2	Description
Cone range (°)	8	3	Search range for the first two Euler angles
Cone sampling (°)	2	1	Sampling increment for cone search
In-plane range (pix.)	2	2	Search range for third Euler angle around the new vertical axis
In-plane sampling (pix.)	1	1	Sampling increment for in-plane search
Threshold	0.8	0.8	Particles when compared to the average that have a cross-correlation value lower than this value are excluded
High-pass filter	2 (80 Å)	2 (80 Å)	Bandpass filter to remove low frequencies. Resolution (Å) = (Pix. Size * Box Size) / Voxels
Low-pass filter	7 (23 Å)	10 (16 Å)	Bandpass filter to remove high frequencies. Resolution (Å) = (Pix. Size * Box Size) / Voxels
Area search [x,y,z]	[12,12,8]	[12,12,8]	Translation limits from center of box
Symmetry	C1	C1	Symmetry for averaging

Supplementary Data:

Data S1: Sequence and structure-based analysis of AcMNPV nucleocapsid and hallmark proteins. Excel spreadsheet presenting the detailed analysis of the core baculovirus proteins and their evolutionary link with viral and cellular homologues, as summarized in Table S3 and Fig. 6A.

Data S2: 3D atlas of all baculovirus core proteins. Zip archive of AlphaFold2 models in PDB coordinate format, with pLDDT in the B-factor column.

REFERENCES AND NOTES

1. M. Shi, X. D. Lin, J. H. Tian, L. J. Chen, X. Chen, C. X. Li, X. C. Qin, J. Li, J. P. Cao, J. S. Eden, J. Buchmann, W. Wang, J. Xu, E. C. Holmes, Y. Z. Zhang, Redefining the invertebrate RNA virosphere. *Nature* **540**, 539–543 (2016).

2. Y. I. Wolf, S. Silas, Y. Wang, S. Wu, M. Bocek, D. Kazlauskas, M. Krupovic, A. Fire, V. V. Dolja, E. V. Koonin, Doubling of the known set of RNA viruses by metagenomic analysis of an aquatic virome. *Nat. Microbiol.* **5**, 1262–1270 (2020).

3. E. V. Koonin, V. V. Dolja, M. Krupovic, A. Varsani, Y. I. Wolf, N. Yutin, F. M. Zerbini, J. H. Kuhn, Global organization and proposed megataxonomy of the virus world. *Microbiol. Mol. Biol. Rev.* **84**, e00061-19 (2020).

4. L. F. Camarillo-Guerrero, A. Almeida, G. Rangel-Pineros, R. D. Finn, T. D. Lawley, Massive expansion of human gut bacteriophage diversity. *Cell* **184**, 1098–1109.e9 (2021).

5. J. R. Brum, J. C. Ignacio-Espinoza, S. Roux, G. Doulcier, S. G. Acinas, A. Alberti, S. Chaffron, C. Cruaud, C. de Vargas, J. M. Gasol, G. Gorsky, A. C. Gregory, L. Guidi, P. Hingamp, D. Iudicone, F. Not, H. Ogata, S. Pesant, B. T. Poulos, S. M. Schwenck, S. Speich, C. Dimier, S. Kandels-Lewis, M. Picheral, S. Searson, P. Bork, C. Bowler, S. Sunagawa, P. Wincker, E. Karsenti, M. B. Sullivan, Ocean plankton. Patterns and ecological drivers of ocean viral communities. *Science* **348**, 1261498 (2015).

6. M. R. Clokie, A. D. Millard, A. V. Letarov, S. Heaphy, Phages in nature. *Bacteriophage* **1**, 31–45 (2011).

7. M. J. Roossinck, The good viruses: Viral mutualistic symbioses. *Nat. Rev. Microbiol.* **9**, 99–108 (2011).

8. M. Voysey, S. A. C. Clemens, S. A. Madhi, L. Y. Weckx, P. M. Folegatti, P. K. Aley, B. Angus, V. L. Baillie, S. L. Barnabas, Q. E. Bhorat, S. Bibi, C. Briner, P. Cicconi, A. M. Collins, R. Colin-Jones, C. L. Cutland, T. C. Darton, K. Dheda, C. J. A. Duncan, K. R. W. Emary, K. J. Ewer, L. Fairlie, S. N. Faust, S. Feng, D. M. Ferreira, A. Finn, A. L. Goodman, C. M. Green, C. A. Green, P. T. Heath, C. Hill, H. Hill, I. Hirsch, S. H. C. Hodgson, A. Izu, S. Jackson, D.

Jenkin, C. C. D. Joe, S. Kerridge, A. Koen, G. Kwatra, R. Lazarus, A. M. Lawrie, A. Lelliott, V. Libri, P. J. Lillie, R. Mallory, A. V. A. Mendes, E. P. Milan, A. M. Minassian, A. M. Gregor, H. Morrison, Y. F. Mujadidi, A. Nana, P. J. O'Reilly, S. D. Padayachee, A. Pittella, E. Plested, K. M. Pollock, M. N. Ramasamy, S. Rhead, A. V. Schwarzbold, N. Singh, A. Smith, R. Song, M. D. Snape, E. Sprinz, R. K. Sutherland, R. Tarrant, E. C. Thomson, M. E. Török, M. Toshner, D. P. J. Turner, J. Vekemans, T. L. Villafana, M. E. E. Watson, C. J. Williams, A. D. Douglas, A. V. S. Hill, T. Lambe, S. C. Gilbert, A. J. Pollard, Oxford COVID Vaccine Trial Group, Safety and efficacy of the ChAdOx1 nCoV-19 vaccine (AZD1222) against SARS-CoV-2: An interim analysis of four randomised controlled trials in Brazil, South Africa, and the UK. *Lancet* **397**, 99–111 (2021).

9. J. Wagemans, D. Holtappels, E. Vainio, M. Rabiey, C. Marzachi, S. Herrero, M. Ravanbakhsh, C. C. Tebbe, M. Ogliastro, M. A. Ayllon, M. Turina, Going viral: Virus-based biological control agents for plant protection. *Annu. Rev. Phytopathol.* **60**, 21–42 (2022).

10. M. M. van Oers, G. P. Pijlman, J. M. Vlak, Thirty years of baculovirus-insect cell protein expression: From dark horse to mainstream technology. *J. Gen. Virol.* **96**, 6–23 (2015).

11. M. L. Pidre, P. N. Arrias, L. C. Amoros Morales, V. Romanowski, The magic staff: A comprehensive overview of baculovirus-based technologies applied to human and animal health. *Viruses* **15**, 80 (2022).

12. M. D. Summers, "Milestones leading to the genetic engineering of baculoviruses as expression vector systems and viral pesticides" in *Insect Viruses: Biotechnological Applications*, Advances in Virus Research (Elsevier, 2006), pp. 3–73.

13. M. M. van Oers, E. A. Herniou, J. A. Jehle, P. J. Krell, A. M. M. Abd-Alla, B. M. Ribeiro, D. A. Theilmann, Z. Hu, R. L. Harrison, Developments in the classification and nomenclature of arthropod-infecting large DNA viruses that contain pif genes. *Arch. Virol.* **168**, 182 (2023).

14. International Committee on Taxonomy of Viruses Executive, The new scope of virus taxonomy: Partitioning the virosphere into 15 hierarchical ranks. *Nat. Microbiol.* **5**, 668–674 (2020).

15. M. Krupovic, V. V. Dolja, E. V. Koonin, The virome of the last eukaryotic common ancestor and eukaryogenesis. *Nat. Microbiol.* **8**, 1008–1017 (2023).

16. J. Iranzo, M. Krupovic, E. V. Koonin, The double-stranded DNA virosphere as a modular hierarchical network of gene sharing. *mBio* **7**, e00978-16 (2016).

17. G. W. Blissard, D. A. Theilmann, Baculovirus entry and egress from insect cells. *Annu. Rev. Virol.* **5**, 113–139 (2018).

18. G. F. Rohrmann, *Baculovirus Molecular Biology* (National Center for Biotechnology Information, ed. 4, 2019).

19. F. Coulibaly, E. Chiu, S. Gutmann, C. Rajendran, P. W. Haebel, K. Ikeda, H. Mori, V. K. Ward, C. Schulze-Briese, P. Metcalf, The atomic structure of baculovirus polyhedra reveals the independent emergence of infectious crystals in DNA and RNA viruses. *Proc. Natl. Acad. Sci. U.S.A.* **106**, 22205–22210 (2009).

20. E. D. Goley, T. Ohkawa, J. Mancuso, J. B. Woodruff, J. A. D'Alessio, W. Z. Cande, L. E. Volkman, M. D. Welch, Dynamic nuclear actin assembly by Arp2/3 complex and a baculovirus WASP-like protein. *Science* **314**, 464–467 (2006).

21. F. M. C. Benning, S. Jenni, C. Y. Garcia, T. H. Nguyen, X. Zhang, L. H. Chao, Helical reconstruction of VP39 reveals principles for baculovirus nucleocapsid assembly. *Nat. Commun.* **15**, 250 (2024).

22. X. Jia, Y. Gao, Y. Huang, L. Sun, S. Li, H. Li, X. Zhang, Y. Li, J. He, W. Wu, H. Venkannagari, K. Yang, M. L. Baker, Q. Zhang, Architecture of the baculovirus nucleocapsid revealed by cryo-EM. *Nat. Commun.* **14**, 7481 (2023).

23. Q. Wang, B. J. Bosch, J. M. Vlak, M. M. van Oers, P. J. Rottier, J. W. M. van Lent, Budded baculovirus particle structure revisited. *J. Invertebr. Pathol.* **134**, 15–22 (2016).

24. J. Jumper, R. Evans, A. Pritzel, T. Green, M. Figurnov, O. Ronneberger, K. Tunyasuvunakool, R. Bates, A. Žídek, A. Potapenko, A. Bridgland, C. Meyer, S. A. A. Kohl, A. J. Ballard, A. Cowie, B. Romera-Paredes, S. Nikolov, R. Jain, J. Adler, T. Back, S.

Petersen, D. Reiman, E. Clancy, M. Zielinski, M. Steinegger, M. Pacholska, T. Berghammer, S. Bodenstein, D. Silver, O. Vinyals, A. W. Senior, K. Kavukcuoglu, P. Kohli, D. Hassabis, Highly accurate protein structure prediction with AlphaFold. *Nature* **596**, 583–589 (2021).

25. F. Wang, V. Cvirkaite-Krupovic, M. Vos, L. C. Beltran, M. A. B. Kreutzberger, J. M. Winter, Z. Su, J. Liu, S. Schouten, M. Krupovic, E. H. Egelman, Spindle-shaped archaeal viruses evolved from rod-shaped ancestors to package a larger genome. *Cell* **185**, 1297–1307.e11 (2022).

26. D. Ptchelkine, A. Gillum, T. Mochizuki, S. Lucas-Staat, Y. Liu, M. Krupovic, S. E. V. Phillips, D. Prangishvili, J. T. Huiskonen, Unique architecture of thermophilic archaeal virus APBV1 and its genome packaging. *Nat. Commun.* **8**, 1436 (2017).

27. A. Villalta, A. Schmitt, L. F. Estrozi, E. R. J. Quemin, J. M. Alempic, A. Lartigue, V. Pražák, L. Belmudes, D. Vasishtan, A. M. G. Colmant, F. A. Honoré, Y. Couté, K. Grünewald, C. Abergel, The giant mimivirus 1.2 Mb genome is elegantly organized into a 30-nm diameter helical protein shield. *eLife* **11**, e77607 (2022).

28. H. J. Huang, S. L. Tang, Y. C. Chang, H. C. Wang, T. H. Ng, R. F. Garmann, Y. W. Chen, J. Y. Huang, R. Kumar, S. H. Chang, S. R. Wu, C. Y. Chao, K. Matoba, I. Kenji, W. M. Gelbart, T. P. Ko, H. A. Wang, C. F. Lo, L. L. Chen, H. C. Wang, Multiple nucleocapsid structural forms of shrimp White Spot Syndrome Virus suggests a novel viral morphogenetic pathway. *Int. J. Mol. Sci.* **24**, 7525 (2023).

29. M. Sun, M. Liu, H. Shan, K. Li, P. Wang, H. Guo, Y. Zhao, R. Wang, Y. Tao, L. Yang, Y. Zhang, X. Su, Y. Liu, C. Li, J. Lin, X. L. Chen, Y. Z. Zhang, Q. T. Shen, Ring-stacked capsids of white spot syndrome virus and structural transitions with genome ejection. *Sci. Adv.* **9**, eadd2796 (2023).

30. D. Bhella, F. J. Rixon, D. J. Dargan, Cryomicroscopy of human cytomegalovirus virions reveals more densely packed genomic DNA than in herpes simplex virus type 1. *J. Mol. Biol.* **295**, 155–161 (2000).

31. M. Krupovic, E. V. Koonin, Homologous capsid proteins testify to the common ancestry of retroviruses, caulimoviruses, pseudoviruses, and metaviruses. *J. Virol.* **91**, e00210–17 (2017).

32. W. R. Wikoff, L. Liljas, R. L. Duda, H. Tsuruta, R. W. Hendrix, J. E. Johnson, Topologically linked protein rings in the bacteriophage HK97 capsid. *Science* **289**, 2129–2133 (2000).

33. V. D'Souza, M. F. Summers, Structural basis for packaging the dimeric genome of Moloney murine leukaemia virus. *Nature* **431**, 586–590 (2004).

34. A. M. Olland, J. Jane-Valbuena, L. A. Schiff, M. L. Nibert, S. C. Harrison, Structure of the reovirus outer capsid and dsRNA-binding protein σ3 at 1.8 Å resolution. *EMBO J.* **20**, 979–989 (2001).

35. Y. Nie, M. Fang, D. A. Theilmann, Autographa californica multiple nucleopolyhedrovirus core gene ac92 (p33) is required for efficient budded virus production. *Virology* **409**, 38–45 (2011).

36. H. Zhang, W. Kuang, C. Fu, J. Li, M. Wang, Z. Hu, AC81 is a putative disulfide isomerase involved in baculoviral disulfide bond formation. *J. Virol.* **96**, e0116722 (2022).

37. H. Zhang, W. Kuang, C. Chen, Y. Shang, X. Ma, F. Deng, H. Wang, M. Wang, Z. Hu, Per os infectivity factor 5 identified as a substrate of P33 in the baculoviral disulfide bond formation pathway. *J. Virol.* **94**, e00615–20 (2020).

38. W. Wu, A. L. Passarelli, Autographa californica multiple nucleopolyhedrovirus Ac92 (ORF92, P33) is required for budded virus production and multiply enveloped occlusion-derived virus formation. *J. Virol.* **84**, 12351–12361 (2010).

39. R. Twarock, A. Luque, Structural puzzles in virology solved with an overarching icosahedral design principle. *Nat. Commun.* **10**, 4414 (2019).

40. D. L. Caspar, A. Klug, Physical principles in the construction of regular viruses. *Cold Spring Harb. Symp. Quant. Biol.* **27**, 1–24 (1962).

41. T. Chen, X. Duan, H. Hu, Y. Shang, Y. Hu, F. Deng, H. Wang, M. Wang, Z. Hu, Systematic analysis of 42 Autographa californica multiple nucleopolyhedrovirus genes identifies an additional six genes involved in the production of infectious budded virus. *Virol. Sin.* **36**, 762–773 (2021).

42. M. Marek, O. W. Merten, F. Francis-Devaraj, M. M. Oers, Essential C-terminal region of the baculovirus minor capsid protein VP80 binds DNA. *J. Virol.* **86**, 1728–1738 (2012).

43. C. B. McCarthy, X. Dai, C. Donly, D. A. Theilmann, Autographa californica multiple nucleopolyhedrovirus ac142, a core gene that is essential for BV production and ODV envelopment. *Virology* **372**, 325–339 (2008).

44. Y. Chen, H. Wu, J. Li, Z. Hu, M. Wang, H. Zhang, Cysteines 128 and 250 are essential for the functions of the baculovirus core gene ac109. *Virology* **587**, 109857 (2023).

45. C. J. Lehiy, W. Wu, M. F. Berretta, A. L. Passarelli, Autographa californica M nucleopolyhedrovirus open reading frame 109 affects infectious budded virus production and nucleocapsid envelopment in the nucleus of cells. *Virology* **435**, 442–452 (2013).

46. M. Fang, Y. Nie, D. A. Theilmann, Deletion of the AcMNPV core gene ac109 results in budded virions that are non-infectious. *Virology* **389**, 66–74 (2009).

47. Y. T. Liu, J. Jih, X. Dai, G. Q. Bi, Z. H. Zhou, Cryo-EM structures of herpes simplex virus type 1 portal vertex and packaged genome. *Nature* **570**, 257–261 (2019).

48. Q. Lai, W. Wu, A. Li, W. Wang, M. Yuan, K. Yang, The 38K-mediated specific dephosphorylation of the viral core protein P6.9 plays an important role in the nucleocapsid assembly of Autographa californica multiple nucleopolyhedrovirus. *J. Virol.* **92**, e01989-17 (2018).

49. Y. Zhang, X. Hu, J. Mu, Y. Hu, Y. Zhou, H. Zhao, C. Wu, R. Pei, J. Chen, X. Chen, Y. Wang, Ac102 participates in nuclear actin polymerization by modulating BV/ODV-C42 ubiquitination during Autographa californica multiple nucleopolyhedrovirus infection. *J. Virol.* **92**, e00005–18 (2018).

50. S. E. Hepp, G. M. Borgo, S. Ticau, T. Ohkawa, M. D. Welch, Baculovirus AC102 is a nucleocapsid protein that is crucial for nuclear actin polymerization and nucleocapsid morphogenesis. *J. Virol.* **92**, e00111–18 (2018).

51. Y. Huang, H. Sun, S. Wei, L. Cai, L. Liu, Y. Jiang, J. Xin, Z. Chen, Y. Que, Z. Kong, T. Li, H. Yu, J. Zhang, Y. Gu, Q. Zheng, S. Li, R. Zhang, N. Xia, Structure and proposed DNA delivery mechanism of a marine roseophage. *Nat. Commun.* **14**, 3609 (2023).

52. V. Zila, E. Margiotta, B. Turoňová, T. G. Müller, C. E. Zimmerli, S. Mattei, M. Allegretti, K. Börner, J. Rada, B. Müller, M. Lusic, H. G. Kräusslich, M. Beck, Cone-shaped HIV-1 capsids are transported through intact nuclear pores. *Cell* **184**, 1032–1046.e18 (2021).

53. P. J. Walker, S. G. Siddell, E. J. Lefkowitz, A. R. Mushegian, E. M. Adriaenssens, P. Alfenas-Zerbini, A. J. Davison, D. M. Dempsey, B. E. Dutilh, M. L. García, B. Harrach, R. L. Harrison, R. C. Hendrickson, S. Junglen, N. J. Knowles, M. Krupovic, J. H. Kuhn, A. J. Lambert, M. Łobocka, M. L. Nibert, H. M. Oksanen, R. J. Orton, D. L. Robertson, L. Rubino, S. Sabanadzovic, P. Simmonds, D. B. Smith, N. Suzuki, K. Van Dooerslaer, A. M. Vandamme, A. Varsani, F. M. Zerbini, Changes to virus taxonomy and to the International Code of Virus Classification and Nomenclature ratified by the International Committee on Taxonomy of Viruses (2021). *Arch. Virol.* **166**, 2633–2648 (2021).

54. F. O. Aylward, M. Moniruzzaman, ViralRecall—A flexible command-line tool for the detection of giant virus signatures in 'Omic Data. *Viruses* **13**, 150 (2021).

55. O. Y. Lung, M. Cruz-Alvarez, G. W. Blissard, Ac23, an envelope fusion protein homolog in the baculovirus Autographa californica multicapsid nucleopolyhedrovirus, is a viral pathogenicity factor. *J. Virol.* **77**, 328–339 (2003).

56. J. Kadlec, S. Loureiro, N. G. Abrescia, D. I. Stuart, I. M. Jones, The postfusion structure of baculovirus gp64 supports a unified view of viral fusion machines. *Nat. Struct. Mol. Biol.* **15**, 1024–1030 (2008).

57. M. C. Vaney, F. A. Rey, Class II enveloped viruses. *Cell. Microbiol.* **13**, 1451–1459 (2011).

58. J. A. Jehle, A. M. Abd-Alla, Y. Wang, Phylogeny and evolution of Hytrosaviridae. *J. Invertebr. Pathol.* **112**, S62–S67 (2013).

59. T. G. Senkevich, C. L. White, E. V. Koonin, B. Moss, A viral member of the ERV1/ALR protein family participates in a cytoplasmic pathway of disulfide bond formation. *Proc. Natl. Acad. Sci. U.S.A.* **97**, 12068–12073 (2000).

60. Y. Hou, Q. Xia, Y. A. Yuan, Crystal structure of Bombyx mori nucleopolyhedrovirus ORF75 reveals a pseudo-dimer of thiol oxidase domains with a putative substrate-binding pocket. *J. Gen. Virol.* **93**, 2142–2151 (2012).

61. M. Hakim, A. Mandelbaum, D. Fass, Structure of a baculovirus sulfhydryl oxidase, a highly divergent member of the Erv flavoenzyme family. *J. Virol.* **85**, 9406–9413 (2011).

62. T. B. Machado, A. C. R. Picorelli, B. L. de Azevedo, I. L. M. de Aquino, V. F. Queiroz, R. A. L. Rodrigues, J. P. Araújo Jr., L. S. Ullmann, T. M. D. Santos, R. E. Marques, S. L. Guimarães, A. C. S. P. Andrade, J. S. Gularte, M. Demoliner, M. Filippi, V. M. A. G. Pereira, F. R. Spilki, M. Krupovic, F. O. Aylward, L.-E. Del-Bem, J. S. Abrahão, Gene duplication as a major force driving the genome expansion in some giant viruses. *J. Virol.* **97**, e01309–23 (2023).

63. N. C. Elde, S. J. Child, M. T. Eickbush, J. O. Kitzman, K. S. Rogers, J. Shendure, A. P. Geballe, H. S. Malik, Poxviruses deploy genomic accordions to adapt rapidly against host antiviral defenses. *Cell* **150**, 831–841 (2012).

64. X. Wang, Y. Shang, C. Chen, S. Liu, M. Chang, N. Zhang, H. Hu, F. Zhang, T. Zhang, Z. Wang, X. Liu, Z. Lin, F. Deng, H. Wang, Z. Zou, J. M. Vlak, M. Wang, Z. Hu, Baculovirus per os infectivity factor complex: Components and assembly. *J. Virol.* **93**, e02053-18 (2019).

65. K. Peng, M. M. van Oers, Z. Hu, J. W. van Lent, J. M. Vlak, Baculovirus per os infectivity factors form a complex on the surface of occlusion-derived virus. *J. Virol.* **84**, 9497–9504 (2010).

66. M. Krupovic, E. V. Koonin, Multiple origins of viral capsid proteins from cellular ancestors. *Proc. Natl. Acad. Sci. U.S.A.* **114**, E2401–E2410 (2017).

67. E. V. Koonin, V. V. Dolja, M. Krupovic, The logic of virus evolution. *Cell Host Microbe* **30**, 917–929 (2022).

68. J. T. Wennmann, J. Keilwagen, J. A. Jehle, Baculovirus Kimura two-parameter species demarcation criterion is confirmed by the distances of 38 core gene nucleotide sequences. *J. Gen. Virol.* **99**, 1307–1320 (2018).

69. A. Bézier, M. Annaheim, J. Herbinière, C. Wetterwald, G. Gyapay, S. Bernard-Samain, P. Wincker, I. Roditi, M. Heller, M. Belghazi, R. Pfister-Wilhem, G. Periquet, C. Dupuy, E. Huguet, A. N. Volkoff, B. Lanzrein, J. M. Drezen, Polydnaviruses of braconid wasps derive from an ancestral nudivirus. *Science* **323**, 926–930 (2009).

70. N. Murphy, J. C. Banks, J. B. Whitfield, A. D. Austin, Phylogeny of the parasitic microgastroid subfamilies (Hymenoptera: Braconidae) based on sequence data from seven genes, with an improved time estimate of the origin of the lineage. *Mol. Phylogenet. Evol.* **47**, 378–395 (2008).

71. F. Coulibaly, E. Chiu, K. Ikeda, S. Gutmann, P. W. Haebel, C. Schulze-Briese, H. Mori, P. Metcalf, The molecular organization of cypovirus polyhedra. *Nature* **446**, 97–101 (2007).

72. J. Hutchings, G. Zanetti, Fine details in complex environments: The power of cryo-electron tomography. *Biochem. Soc. Trans.* **46**, 807–816 (2018).

73. S. Q. Zheng, E. Palovcak, J. P. Armache, K. A. Verba, Y. Cheng, D. A. Agard, MotionCor2: Anisotropic correction of beam-induced motion for improved cryo-electron microscopy. *Nat. Methods* **14**, 331–332 (2017).

74. D. N. Mastronarde, S. R. Held, Automated tilt series alignment and tomographic reconstruction in IMOD. *J. Struct. Biol.* **197**, 102–113 (2017).

75. M. Radermacher, T. Wagenknecht, A. Verschoor, J. Frank, A new 3-D reconstruction scheme applied to the 50S ribosomal subunit of E. coli. *J. Microsc.* **141**, RP1–RP2 (1986).

76. E. F. Pettersen, T. D. Goddard, C. C. Huang, G. S. Couch, D. M. Greenblatt, E. C. Meng, T. E. Ferrin, UCSF Chimera—A visualization system for exploratory research and analysis. *J. Comput. Chem.* **25**, 1605–1612 (2004).

77. C. A. Schneider, W. S. Rasband, K. W. Eliceiri, NIH Image to ImageJ: 25 years of image analysis. *Nat. Methods* **9**, 671–675 (2012).

78. D. Castano-Diez, M. Kudryashev, H. Stahlberg, Dynamo Catalogue: Geometrical tools and data management for particle picking in subtomogram averaging of cryo-electron tomograms. *J. Struct. Biol.* **197**, 135–144 (2017).

79. D. Kimanius, B. O. Forsberg, S. H. W. Scheres, E. Lindahl, Accelerated cryo-EM structure determination with parallelisation using GPUs in RELION-2. *eLife* **5**, e18722 (2016).

80. A. Desfosses, R. Ciuffa, I. Gutsche, C. Sachse, SPRING—An image processing package for single-particle based helical reconstruction from electron cryomicrographs. *J. Struct. Biol.* **185**, 15–26 (2014).

81. K. Zhang, Gctf: Real-time CTF determination and correction. *J. Struct. Biol.* **193**, 1–12 (2016).

82. G. Tang, L. Peng, P. R. Baldwin, D. S. Mann, W. Jiang, I. Rees, S. J. Ludtke, EMAN2: An extensible image processing suite for electron microscopy. *J. Struct. Biol.* **157**, 38–46 (2007).

83. A. Punjani, J. L. Rubinstein, D. J. Fleet, M. A. Brubaker, cryoSPARC: Algorithms for rapid unsupervised cryo-EM structure determination. *Nat. Methods* **14**, 290–296 (2017).

84. T. D. Goddard, C. C. Huang, E. C. Meng, E. F. Pettersen, G. S. Couch, J. H. Morris, T. E. Ferrin, UCSF ChimeraX: Meeting modern challenges in visualization and analysis. *Protein Sci.* **27**, 14–25 (2018).

85. E. F. Pettersen, T. D. Goddard, C. C. Huang, E. C. Meng, G. S. Couch, T. I. Croll, J. H. Morris, T. E. Ferrin, UCSF ChimeraX: Structure visualization for researchers, educators, and developers. *Protein Sci.* **30**, 70–82 (2021).

86. T. Bepler, A. Morin, M. Rapp, J. Brasch, L. Shapiro, A. J. Noble, B. Berger, Positive-unlabeled convolutional neural networks for particle picking in cryo-electron micrographs. *Nat. Methods* **16**, 1153–1160 (2019).

87. G. D. Pintilie, J. Zhang, T. D. Goddard, W. Chiu, D. C. Gossard, Quantitative analysis of cryo-EM density map segmentation by watershed and scale-space filtering, and fitting of structures by alignment to regions. *J. Struct. Biol.* **170**, 427–438 (2010).

88. A. Punjani, H. Zhang, D. J. Fleet, Non-uniform refinement: Adaptive regularization improves single-particle cryo-EM reconstruction. *Nat. Methods* **17**, 1214–1221 (2020).

89. P. K. Purohit, M. M. Inamdar, P. D. Grayson, T. M. Squires, J. Kondev, R. Phillips, Forces during bacteriophage DNA packaging and ejection. *Biophys. J.* **88**, 851–866 (2005).

90. W. H. Roos, K. Radtke, E. Kniesmeijer, H. Geertsema, B. Sodeik, G. J. Wuite, Scaffold expulsion and genome packaging trigger stabilization of herpes simplex virus capsids. *Proc. Natl. Acad. Sci. U.S.A.* **106**, 9673–9678 (2009).

91. P. D. Adams, P. V. Afonine, G. Bunkóczi, V. B. Chen, I. W. Davis, N. Echols, J. J. Headd, L. W. Hung, G. J. Kapral, R. W. Grosse-Kunstleve, A. J. McCoy, N. W. Moriarty, R. Oeffner, R. J. Read, D. C. Richardson, J. S. Richardson, T. C. Terwilliger, P. H. Zwart, PHENIX: A comprehensive Python-based system for macromolecular structure solution. *Acta Crystallogr. D Biol. Crystallogr.* **66**, 213–221 (2010).

92. P. Emsley, B. Lohkamp, W. G. Scott, K. Cowtan, Features and development of Coot. *Acta Crystallogr. D Biol. Crystallogr.* **66**, 486–501 (2010).

93. C. J. Williams, J. J. Headd, N. W. Moriarty, M. G. Prisant, L. L. Videau, L. N. Deis, V. Verma, D. A. Keedy, B. J. Hintze, V. B. Chen, S. Jain, S. M. Lewis, W. B. Arendall III, J. Snoeyink, P. D. Adams, S. C. Lovell, J. S. Richardson, D. C. Richardson, MolProbity: More and better reference data for improved all-atom structure validation. *Protein Sci.* **27**, 293–315 (2018).

94. T. I. Croll, ISOLDE: A physically realistic environment for model building into low-resolution electron-density maps. *Acta Crystallogr. D Struct. Biol.* **74**, 519–530 (2018).

95. M. Mirdita, K. Schütze, Y. Moriwaki, L. Heo, S. Ovchinnikov, M. Steinegger, ColabFold: Making protein folding accessible to all. *Nat. Methods* **19**, 679–682 (2022).

96. E. Krissinel, K. Henrick, Inference of macromolecular assemblies from crystalline state. *J. Mol. Biol.* **372**, 774–797 (2007).

97. H. Ashkenazy, S. Abadi, E. Martz, O. Chay, I. Mayrose, T. Pupko, N. Ben-Tal, ConSurf 2016: An improved methodology to estimate and visualize evolutionary conservation in macromolecules. *Nucleic Acids Res.* **44**, W344–W350 (2016).

98. M. M. Suhanovsky, C. M. Teschke, Nature's favorite building block: Deciphering folding and capsid assembly of proteins with the HK97-fold. *Virology* **479-480**, 487–497 (2015).

99. B. Boogaard, M. M. van Oers, J. W. M. van Lent, An advanced view on baculovirus per os infectivity factors. *Insects* **9**, 84 (2018).

100. X. Wang, X. Liu, G. A. Makalliwa, J. Li, H. Wang, Z. Hu, M. Wang, Per os infectivity factors: A complicated and evolutionarily conserved entry machinery of baculovirus. *Sci. China Life Sci.* **60**, 806–815 (2017).

101. D. Kazlauskas, M. Krupovic, J. Guglielmini, P. Forterre, Č. Venclovas, Diversity and evolution of B-family DNA polymerases. *Nucleic Acids Res.* **48**, 10142–10156 (2020).

102. U. Rosani, M. Gaia, T. O. Delmont, M. Krupovic, Tracing the invertebrate herpesviruses in the global sequence datasets. *Front. Mar. Sci.* **10**, 1159754 (2023).

103. S. Shuman, What messenger RNA capping tells us about eukaryotic evolution. *Nat. Rev. Mol. Cell Biol.* **3**, 619–625 (2002).

104. O. J. Kyrieleis, J. Chang, M. de la Peña, S. Shuman, S. Cusack, Crystal structure of vaccinia virus mRNA capping enzyme provides insights into the mechanism and evolution of the capping apparatus. *Structure* **22**, 452–465 (2014).

105. M. Krupovic, V. V. Dolja, E. V. Koonin, Origin of viruses: Primordial replicators recruiting capsids from hosts. *Nat. Rev. Microbiol.* **17**, 449–458 (2019).

106. L. M. Iyer, E. V. Koonin, D. D. Leipe, L. Aravind, Origin and evolution of the archaeo-eukaryotic primase superfamily and related palm-domain proteins: Structural insights and new members. *Nucleic Acids Res.* **33**, 3875–3896 (2005).

107. J. T. Evans, D. J. Leisy, G. F. Rohrmann, Characterization of the interaction between the baculovirus replication factors LEF-1 and LEF-2. *J. Virol.* **71**, 3114–3119 (1997).
