# Supplementary material for: The nucleocapsid architecture and structural atlas of the prototype baculovirus define the hallmarks of a new viral realm
Source: Sci Adv. 2024 Dec 18;10(51):eado2631. doi: 10.1126/sciadv.ado2631 (PMC11654684; doi:10.1126/sciadv.ado2631)
Supplement: Supplementary file 2 — Data S1 and S2 [file sciadv.ado2631_data_s1_and_s2.zip › ado2631_data_s2.zip!/Others/AcMNPV_Ac146/Ac146.fasta]

>Ac146
MNVNLYCPNGEHDNNISFIMPSKTNAVIVYLFKLDIEHPPTYNVNAKTRLVSGYENSRPININLRSITPSLNGTRGAYVISCIRAPRLYRDLFAYNKYTAPLGFVVTRTHAELQVWHILSVRKTFEAKSTRSVTGMLAHTDNGPDKFYAKDLMIMSGNVSVHFINNLQKCRAHHKDIDIFKHLCPELQIDNSVVQLETRSS
